# Integrated rare variant-based risk gene prioritization in disease case-control sequencing studies

**DOI:** 10.1371/journal.pgen.1007142

**Published:** 2017-12-27

**Authors:** Jhih-Rong Lin, Quanwei Zhang, Ying Cai, Bernice E. Morrow, Zhengdong D. Zhang

**Affiliations:** Department of Genetics, Albert Einstein College of Medicine, Bronx, New York, United States of America; Case Western Reserve University, UNITED STATES

## Abstract

Rare variants of major effect play an important role in human complex diseases and can be discovered by sequencing-based genome-wide association studies. Here, we introduce an integrated approach that combines the rare variant association test with gene network and phenotype information to identify risk genes implicated by rare variants for human complex diseases. Our data integration method follows a 'discovery-driven' strategy without relying on prior knowledge about the disease and thus maintains the unbiased character of genome-wide association studies. Simulations reveal that our method can outperform a widely-used rare variant association test method by 2 to 3 times. In a case study of a small disease cohort, we uncovered putative risk genes and the corresponding rare variants that may act as genetic modifiers of congenital heart disease in 22q11.2 deletion syndrome patients. These variants were missed by a conventional approach that relied on the rare variant association test alone.

## Author summary

Case-control sequencing studies are a promising design to uncover risk genes of human complex diseases implicated by rare variants. The recent development of different types of rare variant association tests has improved the statistical power to identify disease genes that harbor risk rare variants. However, none of the recent sequencing-based genome-wide association studies identified robust disease association of rare variants or genes based on them. Due to limited sample sizes that can be feasibly achieved in real applications, current rare variant association tests can only generate marginal association signals for most risk genes. Here we proposed an integrated method that combined association signals with orthogonal biological evidence to uncover risk genes in sequencing studies. Designed to address the lack-of-power issue, our method was shown to effectively uncover risk genes with marginal association signals in data simulation. Indeed, in a real application demonstrated in our case study our method disclosed important risk genes of congenital heart disease in 22q11.2 deletion syndrome that were missed by the previous study.

## Introduction

The discovery of genes important to human complex diseases has recently involved association studies, which involve testing for allele frequency differences between cases and controls from a given population. Due to the design of the initial microarray-based genotyping method, such studies have hitherto mostly examined the disease association of common variants [1]. The 'missing heritability' of common traits suggests that rare variants may significantly contribute to the genetics of human complex diseases [2]. With the rapid increase in throughput and decrease in its cost, next-generation sequencing has been increasingly used for genotyping in human disease studies. In contrast to traditional genome-wide association studies (GWAS) designed for common variants, case-control whole genome or exome sequencing (WGS/WES) studies provide opportunities to uncover risk genes of complex diseases implicated by rare variants in a genome-wide and hypothesis-free fashion. Low minor allele frequencies preclude detection of disease association of individual rare variants. Instead, biologically relevant variants are aggregated into variant sets (often corresponding to genes [3] or biological pathways), and the aggregate frequency distribution is compared between cases and controls. Although several types of rare variant association tests have been developed to fit different genetic models [3, 4], these tests are underpowered–and will remain so for the foreseeable future–to detect disease association for rare variants due to limited sample sizes [5–7] that can be feasibly achieved. In addition to improving the statistical power of the rare variant association test, there is an urgent need for methods that can analyze rare variants in sequencing-based association studies to identify disease risk genes among the great majority of unrelated genes despite the insufficient discriminative power of genetic association signals.

To tackle this challenge, integration of diverse and yet complementary biological information will be of critical importance. Genes causing the same or similar diseases tend to lie close to one another in a protein-protein interaction network or a gene functional linkage network [8, 9]. Genes involved in the same biological process show similar loss-of-function phenotypes and thus are associated with the same or similar diseases. As a result, gene networks and phenotypes are particularly informative for disease studies and have been widely used to improve risk gene identification in sequencing studies [10–14]. Methods that use network and phenotype information to improve disease risk gene prediction exploit the 'guilt-by-association' network property of risk genes and utilize known connections between genes and disease phenotypes. Their integration with gene association signals, however, needs careful consideration. To predict or prioritize genes for disease association, most current methods with network and phenotype integration require prior knowledge about the disease under investigation–e.g., known risk genes and/or known disease phenotypes–as part of the data input [10, 12, 13, 15]. Devising an integration strategy that does not rely on such prior knowledge would have several potential advantages. First, the application would not be limited to well-studied diseases. Second, and more importantly, such a method would mitigate the limitation that the prior knowledge could predispose the risk gene prediction toward known disease genes and thus compromise the opportunities for novel unbiased biological discovery in genome-wide sequencing studies. In fact, the importance of not relying on prior disease knowledge has been recognized in studies of common variants and common diseases as well, as methods of risk gene prioritization in GWAS using such an unbiased approach have been recently developed [16, 17].

Here, we propose Integrated Gene Signal Processing (IGSP), a novel method to prioritize genes implicated by rare variants for disease risk in sequencing-based GWAS. The IGSP method scores genes by integrating their disease association signals using both gene network as well as phenotype information. In this approach, we proposed a gene scoring model that can improve upon disease association signals for prioritizing risk genes by leveraging the underlying network and phenotype properties of disease risk genes. Given observed disease association signals across the applied gene network and phenotypes, IGSP infers the likelihood of risk genes based on the proposed scoring model, in a stochastic process of sampling possible risk genes combinations. Using simulated data, we systematically evaluated and compared the performance of IGSP against a widely-used method based on the association test alone. As an example, we applied IGSP to the WES data from a case-control association study [18] of congenital heart disease (CHD) in 22q11.2 deletion syndrome (22q11.2DS; velo-cardio-facial syndrome/DiGeorge syndrome; MIM#192430; 188400). Despite a small cohort size and complex disease mechanisms, IGSP was able to uncover putative risk genes of CHD among individuals with 22q11.2DS.

## Results

**Fig 1:**
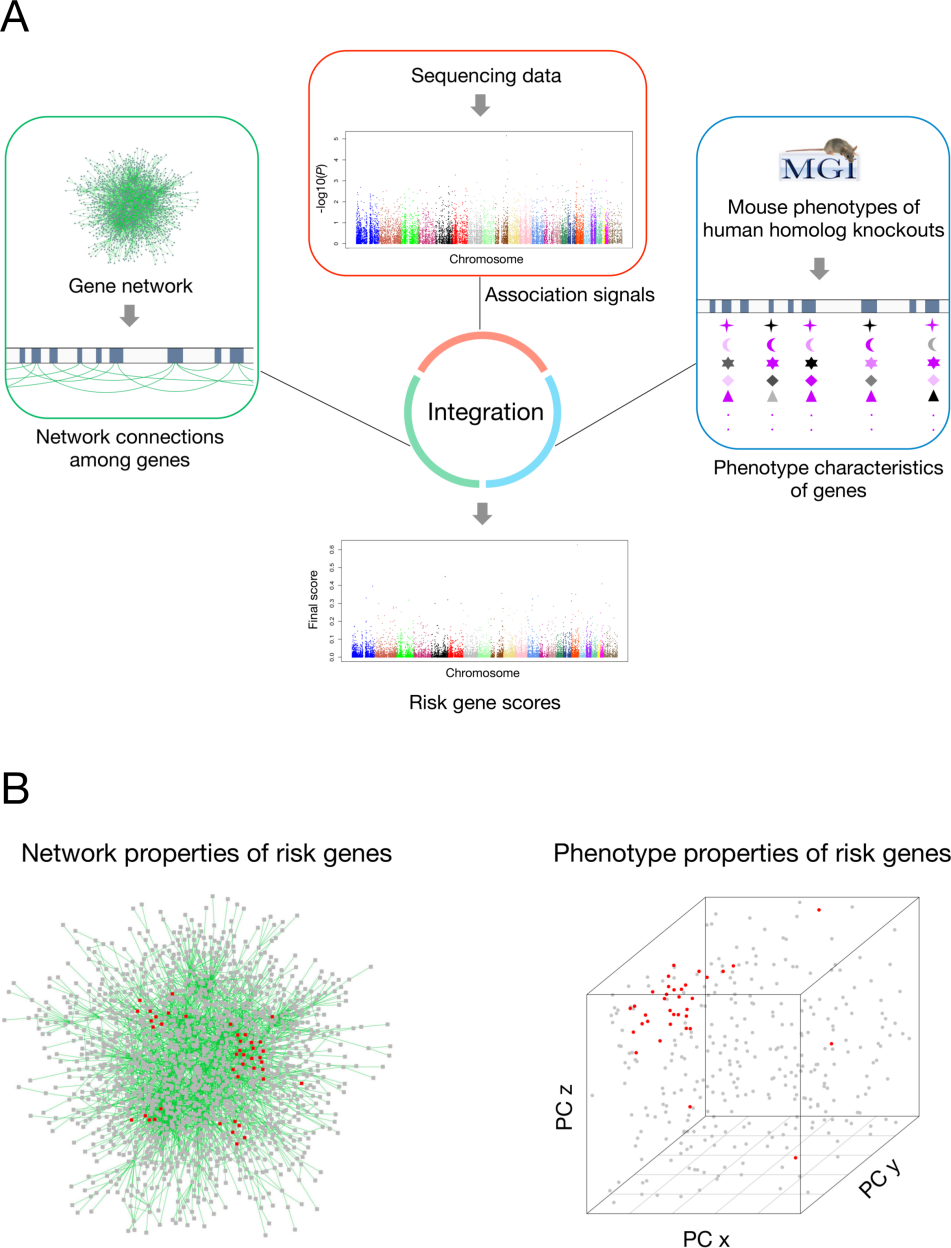
Overview of the IGSP strategy. (A) The schematic view of IGSP. IGSP scores genes by integrating initial gene association signals with available gene network and mouse knock-out phenotype information. Sequencing data represented the primary data for investigation. Gene network and phenotype data were used to support this data and were not specific to any disease. For phenotype characteristics of genes, different symbols represent different principal components from MGI phenotype annotation (see Methods), and the color represents the values of principal components; color of black to purple represents small to large values. (B) The gene network and phenotype properties of risk genes. We hypothesize that risk genes for the disease in the study cohort tend to lie close to each other in a co-function gene network [17] (see Methods) and their orthologs in mice tend to influence similar mouse knock-out phenotypes. PC denotes principal components; red dots represent risk genes while grey dots represent non-risk genes (see S2 Fig).

The strategy of IGSP is to score genes based on not only their association signals but also the joint evaluation of their network and phenotype characteristics (**Fig 1**). By taking advantage of the high-level network properties and phenotype characteristics of risk genes, we hypothesize this method can improve risk gene prediction without relying on explicit knowledge of the disease under investigation. The key premise of our algorithm is to supplement disease association signals with network and phenotype information to boost sensitivity of risk gene prediction.

To justify the hypothesized network and phenotype properties of risk genes in IGSP, we collected risk genes of 5 different diseases from the online Mendelian Inheritance in Man (OMIM) database [19] (**S1 Table**) and investigated the network and phenotype properties of risk genes. Consistent with our hypothesized network properties of risk genes, disease risk genes share significantly more network connections in the co-function network [17] (**S1 Fig**). On the other hand, analysis of top principal components of mouse knock-out phenotypes showed that the same disease risk genes tend to cluster on top principal components of Mammalian Phenotype (MP) phenotypes (**S2 Fig**). The first principal component was excluded from consideration since it mainly characterizes the number of associated MP terms of a gene (correlation coefficient = 0.918), which is not biological meaningful. The observed network and phenotype properties accounts for how network and phenotype-based scoring can measure the underlying connection of genes with disease risk.

IGSP assesses the likelihood of disease risk through jointly evaluating association scores of genes and their network and phenotype properties. It is important to know if there is any relationship between these scoring components due to potential biases: genes with higher network degrees are more likely to be connected in a network, while genes associated with more MP phenotypes are more likely to share phenotype similarities [20]. Using our CHD WES data set, we checked and confirmed that there is no relationship between the genotype-based scoring and the co-function network degree or the number of associated MP terms (**S3 Fig**).

### Method evaluation and comparison

First, we optimized IGSP by testing different values for parameters (see Methods)–the percentage of scoring genes being risk genes (*x*), parameters of scaling coefficient (*a* and *b*), and the number of top principal components from our Mouse Genome Informatics (MGI) phenotype annotation–by using in gene scoring and selecting parameter values that give the best results. IGSP assumes that *x*% of genes to be scored are risk genes. In our simulation, 147 (~1.64%) and 193 (~2.15%) genes were deemed CHD and schizophrenia risk genes (**S2** & **S3 Tables**), respectively. The simulation results showed that IGSP achieves the best performance when *x* is set at 1 or 2 (**S4 Fig**) for CHD and schizophrenia, respectively. As expected, the performance gradually decreases as *x* exceeds its real value. Although *x* is unknown in real applications, the simulation result showed that IGSP can largely maintain its performance if *x* is set within a reasonable range of its real value. Parameters *a* and *b* in Eq 1 define the scaling range in IGSP. Our simulation with different values of *a* and *b* implies that no single *a* and *b* values are optimal in all scenarios (**S5 & S6 Figs**). Nevertheless, when *a* and b are set to 0.1 and 1, respectively, IGSP can consistently achieve top performance. Principal components of MGI phenotype annotations used in phenotype scoring characterize the dimensions of phenotype features of risk genes. The second and third principal components were able to effectively capture the gene-phenotype association information and are therefore suited for phenotype scoring (**S7 Fig**).

**Fig 2:**
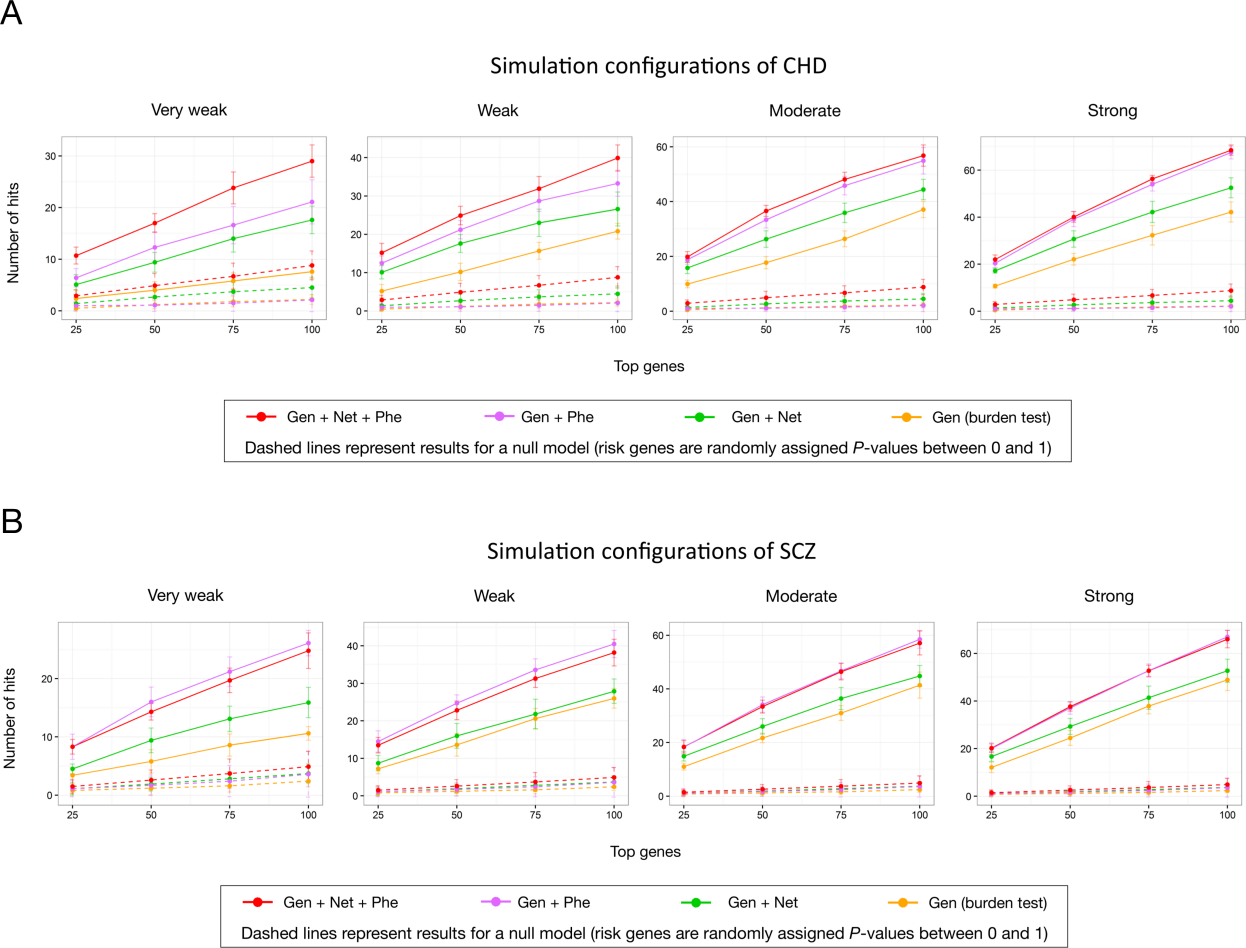
The simulation to evaluate IGSP. The performance of risk gene prioritization was evaluated based on the number of risk genes included in top scoring genes. The labels 'Strong', 'Moderate', 'Weak' and 'Very Weak' define simulation configurations of detected different association signal strength of risk genes on a relative rather than absolute basis (**S4 Table**). 'Gen + Net + Phe' represent IGSP with full integration of network and phenotype features. 'Gen + Net' and 'Gen + Phe' represent IGSP with only integration of network features and phenotype features, respectively. The parameters used in this simulation were as follows: *x* = 2, *a* = 0.1, *b* = 1, and principal components in phenotype scoring (PC 2 and 3). (A) CHD. 147 CHD genes from Sifrim et al [26] were used as the risk genes. (B) Schizophrenia. 193 putative schizophrenia genes from MalaCards [57] were used as the risk genes.

We used simulated association signals of genes for CHD to evaluate the performance of IGSP. CHD, involving the structure of the heart and great vessels is the most common serious birth defect, with a prevalence of 1% in newborns [21–23]. The causes of CHD are still mostly unknown but clearly have a genetic component [24, 25]. To start our data simulation, we first obtained 147 CHD risk genes [26] that can be scored by network and phenotype (**S2 Table**) as risk genes. The other 8,812 genes were designated as non-risk genes. We used IGSP to score the total of 8,959 genes in several simulation configurations: 147 CHD risk genes were randomly assigned a different proportion of low association *P*-values (**S4 Table**), while 8,812 non-risk genes were randomly assigned a *P*-value between 0 and 1. Compared with using disease association signals alone, gene scoring with data integration showed a significant improvement in risk gene prioritization. Judged by the correct identification of CHD risk genes among the top-scoring genes, IGSP outperformed the burden test by 2 to 3 times under different simulation configurations of rare variant association test results (**Fig 2A**). We also carried out simulation analysis with 193 risk genes (**S3 Table**) associated with schizophrenia, another complex human disease, and observed a consistent improvement in risk gene prioritization by IGSP (**Fig 2B**). In both cases, the improved performance resulted solely from the integration of the gene network and phenotype data, as the association signals generated by the burden test were the primary data input to IGSP. Depending on the disease, the network and phenotype components of IGSP may have different effects on risk gene prediction. When they complement each other, however, integrating both can significantly outperform using either one on its own.

In our scoring model, we used normalized scores to represent the relative strength of network and phenotype evidence on a scale from 0 to 1. This raw-score normalization is necessary since raw scores of network and phenotype do not have the same scale (**S8 Fig**). Furthermore, raw network and phenotype scores span within an unpredictable range which makes the control of a scaling range difficult. For example, the scaling range can be too small to make improvement using raw network and phenotype scores (**S9 Fig**). On the other hand, it is flexible to use different raw-score normalization methods. By using a min-max normalization method, we showed that IGSP made comparable improvement (**S10 Fig**). IGSP also allows flexibility for different types of connectivity propensity measured in network-based scoring (**S11 Fig**). By considering the degrees of possible risk genes, the implemented method using a transition matrix considers potential statistical artefacts in assessing guilt by association [20]. On the other hand, the method of measuring connectivity propensity based on connection counts also makes biological sense especially for uncovering risk genes involved in the same functional module associated with disease pathogenesis. Our result showed that IGSP using two methods exhibited similar improvement.

To further demonstrate that our current model effectively integrates network and phenotype features with association signals, we compared the performance of using the current integrated model (Eq 1) with that of using four alternatives by simulation (**S12 Fig**). In all four alternatives (**S12A Fig**), the weight of network and phenotype components was dependent on risk gene status. Model 1 and 2 failed as their power of prioritizing risk genes almost completely lost. It was because in the two alternative integrated models there were no lower bounds (The lower bound is 0) on the scaling coefficient to prevent the scoring being simply dominated by the network and phenotype features. While model 3 and 4 exhibited certain degree of performance improvement over the burden test, they were outperformed by the current integrated model. Model 4 performed better than model 3, as it maintained a minimum weight of the phenotype and network components which is thus less dependent on risk gene status. This suggested that the weight of network and phenotype components should be kept independent of risk gene status, which was already considered in phenotype and network scoring. In summary, the simulation results of using different alternative integrated models justify our current integration design (Eq 1) in that the integrated model needs to have a lower-bound on the scaling coefficient and a weight of the phenotype and network component independent of risk gene status.

To score genes, IGSP modulates their disease association signals by integrating network and phenotype information. Because such information is extrinsic to the data generation process of the association study, it is important to assess how much this information contributes to the final scoring of genes. To carry out this negative control experiment using simulation, we randomly re-assigned simulated association *P*-values of all genes, including risk and non-risk genes, to themselves and used IGSP to score genes with the randomized association signals. The results of this negative control test, based on the null model (**S13 Fig**), showed that when there is a strong tendency for risk genes to be more strongly associated than non-risk genes, IGSP can identify top genes with significantly higher scores compared to IGSP results from merely random association signals. This is because when there is a group of genes with stronger association signals which happens to share network and phenotype similarities, they will be scored higher if they are included in the analysis as integrated scores. This null model analysis shows that our data integration method uses first and foremost association signals to score genes for their disease connection.

### CHD in 22q11.2DS as a model to study rare variants in human complex diseases

CHD, involving the structure of the heart and great vessels, is the most common birth defects with a prevalence of 1% in newborns [21–23]. Its cause is still mostly unknown but has a clear genetic component [24, 25]. Support for a genetic basis of CHD also comes from studies of patients with chromosomal structural variant abnormalities such as 22q11.2DS. This disorder is the most common among microdeletion syndromes, found in 1/4,000 live births [27] and 1/1,000 fetuses [28]. It occurs as a *de novo* 1.5~3 million base pair (Mb) deletion in most individuals [29]. Approximately 60–70% of patients with 22q11.2DS have broadly defined CHD. Most have conotruncal heart defects affecting the cardiac outflow tract and/or aortic arch. Approximately half of these require surgery for survival. Some of the more severe conotruncal heart defects include tetralogy of Fallot and persistent truncus arteriosus. A subset of patients also has atrial septal defects. Conotruncal heart defects comprise a third of the CHD population [30]. Thus, 22q11.2DS can serve as a model to identify risk genes for this relatively common class of CHD. Further, compared to isolated sporadic CHD, syndromic CHD provides better opportunities to identify key risk factors in CHD pathogenesis. Since the frequency and type of conotruncal heart defects vary, it is likely that haploinsufficiency of genes in the 22q11.2 region as well as other genetic factors are responsible. Risk genes for syndromic CHD in 22q11.2DS were recently examined in a WES-based association study with 90 cases and 94 controls [18]. It focused on chromatin modifier genes for CHD risk as implicated in previous studies of the disease and analyzed mildly deleterious rare variants with the expectation that modifiers of CHD would not be extremely deleterious [18]. The study identified chromatin modification as an important risk factor for CHD in 22q11.2DS. However, other risk factors for CHD in 22q11.2DS are yet to be explored.

**Fig 3:**
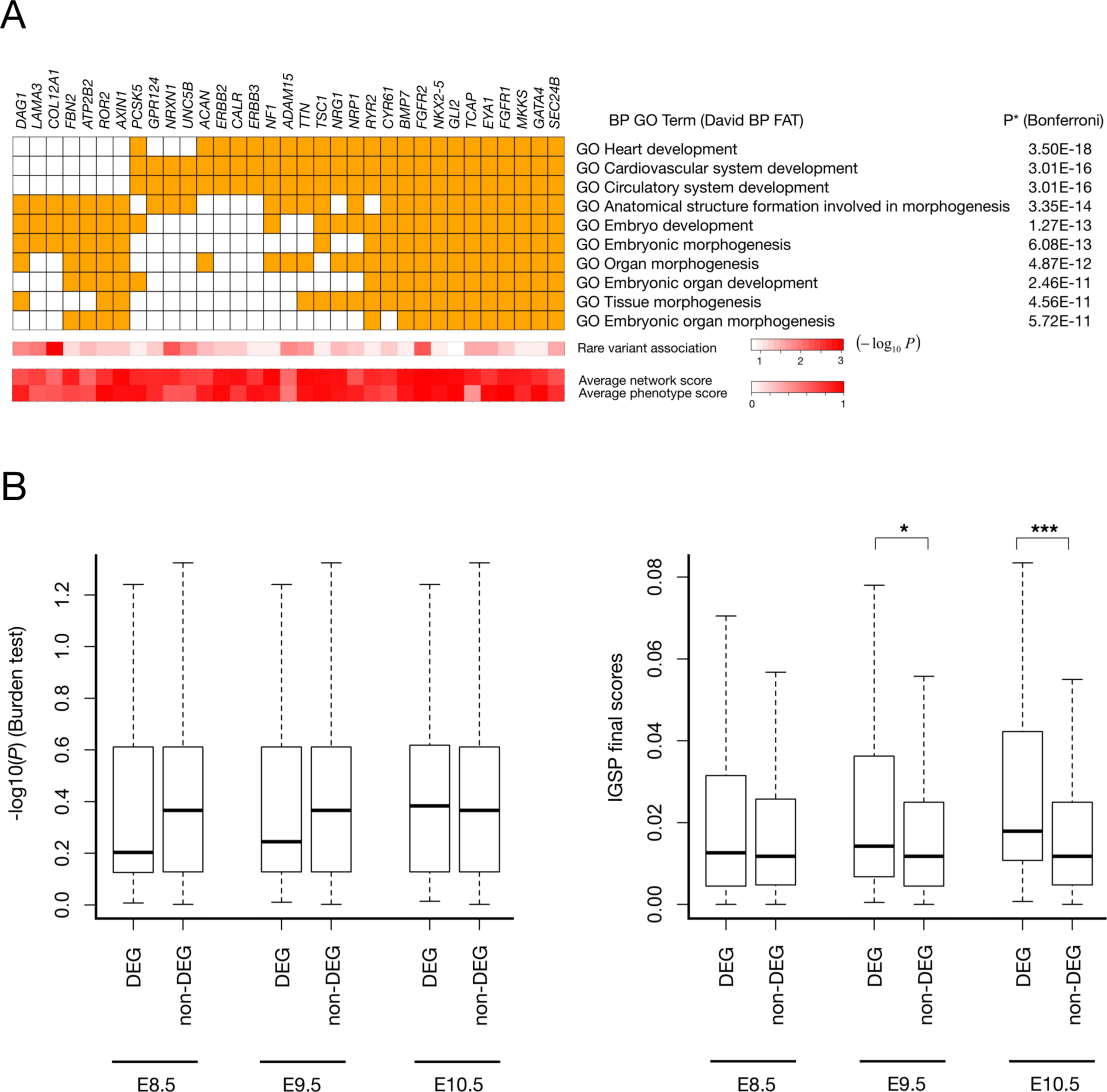
IGSP scoring reveals risk factors in CHD. (A) Top 50 prioritized genes are enriched with GO terms related to heart development. The figure shows top 10 enriched GO terms and the associated top 50 prioritized genes on the left. The complete list for high scoring genes and GO term enrichment result are shown in **S5** & **S6 Tables**. Average network and phenotype scores are calculated based on their average scores in iterations of algorithm. (B) The human homologs of differentially expressed genes (DEG) in *Tbx1* knock-out mice tend to have high IGSP scores. The human homologs of *Tbx1*-induced DEG tend to have high IGSP scores especially in E9.5 (*P* = 0.023) and E10.5 (*P* = 5.5E-4). The Wilcoxon rank-sum test was used to calculate *P*-values.

To serve as a test case for our new data integration method, we used a more stringent variant selection and applied IGSP to this small-sample data set to prioritize genes for CHD risk in an unbiased approach. The burden test had failed to identify genes with significant disease association. 12,196 genes were found to have at least one rare predicted deleterious single nucleotide variant (SNV) in the cohort according to our criteria and thus have an association signal. After optimization with *x* = 2, *a* = 0.1, *b* = 1, and using the second and third principal components used in phenotype scoring, we used IGSP to score 5,987 genes for which both network and phenotype information was available. Functional enrichment analysis showed that the top 50 high-scoring genes were significantly enriched with gene ontology (GO) terms for heart development (**Fig 3A** and **S5 & S6 Tables**), among which the GO term, 'anatomical structure formation' involved in morphogenesis, was the one and the only one implicated among top 50 genes with the strongest association test *P*-values (**S7 Table**). This result indicated that IGSP effectively expanded and uncovered biological signals hidden in the association signals of the CHD study cohort. Indeed, the result of negative control analysis (**S13E Fig**) provided strong evidence that this result of IGSP was derived from gene association signals from the genotype data in the cohort. Despite a very small sample size, IGSP successfully identified in this cohort several known key risk genes–*NKX2-5* (NK2 homeobox 5), *GATA4* (GATA binding protein 4), and *EYA1* (EYA transcriptional coactivator and phosphatase 1)–for CHD in humans or for heart development in animal models [31, 32]. Although these genes showed clear disease association signals, upon close inspection of their genotypes among cases and controls (**Table 1**) these genes all had individual *P*-values that were far from statistically significant because of the low frequencies of rare predicted deleterious alleles. Thus, if only the association test was used for risk gene prioritization, these genes would have been buried in noise and missed. For example, in both *NKX2-5* and in *GATA4* (**Table 1**), two well-known CHD risk genes, two cases but no controls had a rare predicted deleterious SNV, explaining the weak genotype evidence as reflected in the association *P*-values (0.119) of the two genes. IGSP was able to detect the marginal but bona fide signals of these two genes because the network and phenotype information was used. The high IGSP scores we found implied the association of these risk genes with other risk genes in this cohort which could be detected by IGSP using the gene network and phenotype data. On the other hand, *TBX5*, another well-known heart development and CHD risk gene [31], had a low final score (ranked at 1,209) due to lack of genotype evidence (*P* = 0.62) despite its strong relationship with heart development as reflected by its high network and phenotype scores.

**Table 1:** Top scoring IGSP and non-prioritized CHD genes in a WES case control study of rare variants in 22q11.2DS with and without CHD.

Gene						Variant					
Name	B	*P*	Net	Phe	Rank	Coordinate (hg19)	Ref:Alt	B	Functional Class	AAF	C
***NKX2-5***	2:0	0.119	0.974	0.933	27	chr5:172662026	C:G	2:0	Missense variant	0.0054:NA	26.4
***GATA4***	2:0	0.119	0.816	0.968	36	chr8:11612576	C:A	1:0	Synonymous variant	0.0027:NA	20.5
						chr8:11615955	G:A	1:0	Missense variant	0.0027:NA	25.1
***EYA1***	4:0	0.028	0.786	0.959	10	chr8:72267083	G:C	3:0	Missense variant	0.0082:0.001	20.6
						chr8:72267127	T:G	1:0	Missense variant	0.0027:NA	30.0
***TBX5***	1:2	0.62	0.893	0.761	1209	chr12:114837349	C:A	1:1	Missense variant	0.0054:0.006	34.0
						chr12:114841688	C:T	0:1	Missense variant	0.0027:NA	34.0

*NKX2-5*, *GATA4*, *EYA1* and *TBX5* are all genes with known association with heart development, and they were all scored high by network and phenotype. Among the four genes, *NKX2-5*, *GATA4*, and *EYA1* have at least a marginal association signal and thus been effectively prioritized. Variants of the three genes on the right are thus putative genetic modifiers for CHD in 22q11.2DS. B denotes the ratio of the counts of rare predicted deleterious alleles in cases to that in controls; *P* denotes *P*-value from the burden test; Net and Phe represent the average network and phenotype scores (in iterations before reaching convergence), respectively; AAF denotes alternative allele frequency (the cohort of sequencing data: 1000 Genome EUR population); C denotes Combined Annotation Dependent Depletion (CADD) scaled score [51].

Previous studies of gene inactivation in mouse models [33, 34] and mutation analysis of *TBX1* in phenocopies of 22q11.2DS but without a deletion in human patients [35, 36] provide strong evidence for contribution to CHD from *TBX1* (T-box 1), a transcription factor gene located in the 22q11.2 deletion region. To elucidate how *TBX1* target genes may be implicated in the CHD WES data set, we utilized data from gene expression microarrays to analyze human homologs of genes differentially expressed in microdissected cardiac progenitor tissues expressed between *Tbx1* knock-out mouse embryo and normal embryo at three different embryonic days–E8.5, E9.5, and E10.5 [37]. At each time point a gene was identified as a differentially expressed gene (DEG) if it had at least one probe with a nominal *P*-value for differential expression smaller than 0.05 and a fold change greater than 1.5. We also collected strictly non-differentially expressed genes (non-DEGs) as genes whose probes all had a nominal *P*-value greater than 0.5 and a fold change smaller than 1.5. At the aforementioned three mouse embryonic time points, there were 110, 93, and 78 DEGs and 1,591, 2,213, and 2,423 non-DEGs, respectively, that had a IGSP score in integrated scoring. Human homologs of mouse DEGs induced by *Tbx1* knock-out tend to have higher IGSP scores at E9.5 and E10.5 (*P* = 0.023 and 5.5E-4, respectively; **Fig 3B**). In contrast, the difference between *Tbx1*-induced DEGs and non-DEGs was not detected by association scores from the burden test (**Fig 3B**).

**Fig 4:**
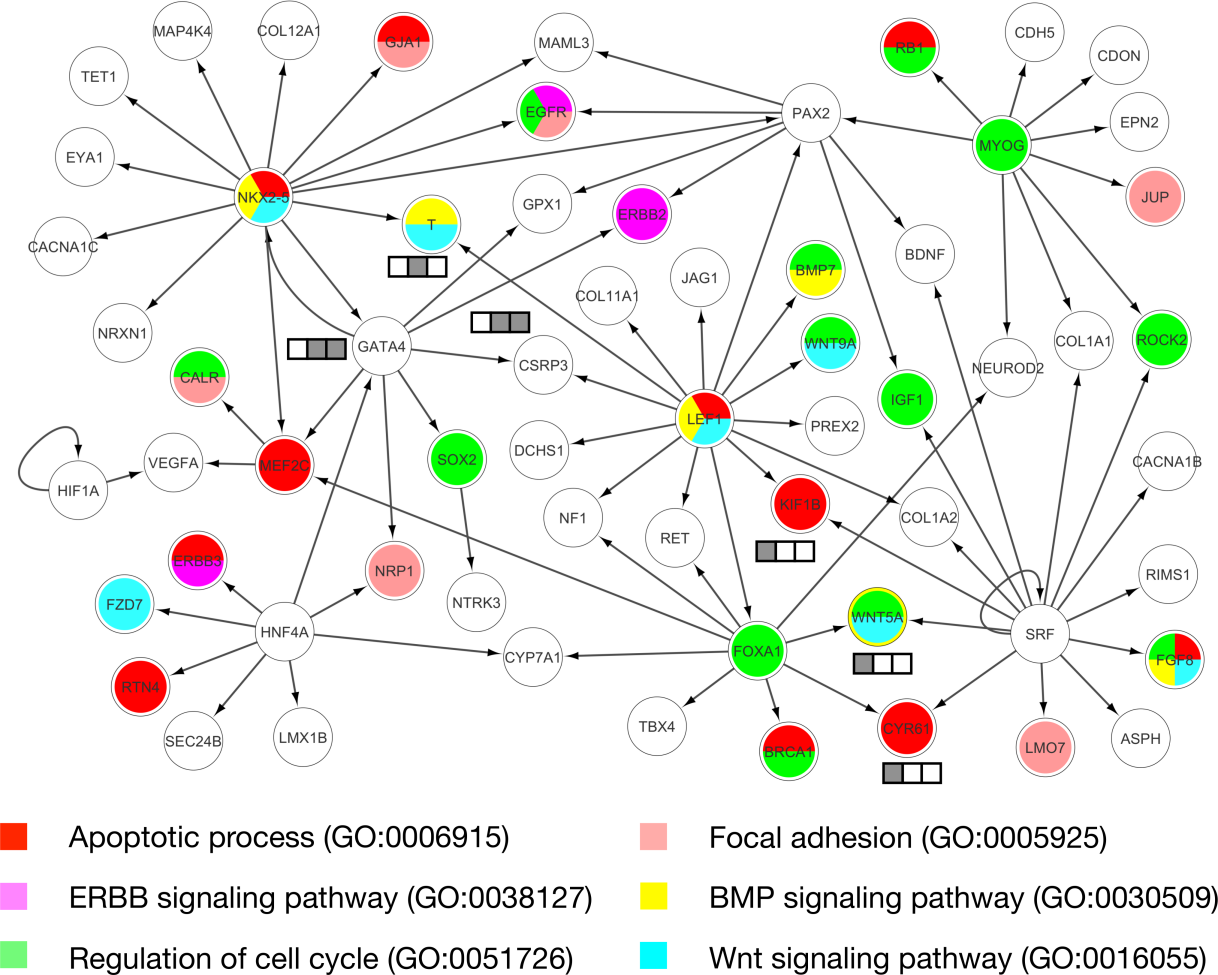
Transcriptional regulatory network derived from IGSP high scoring genes. The box denotes genes perturbed by *TBX1* haploinsufficiency implicated in the mouse model. The color denotes the implicated embryonic time points: E8.5, E9.5, and E10.5 from left to right.

Gene products function in biological pathways. Uncovering pathways of risk genes of CHD could help to unravel the disease pathogenesis. Functional and pathway enrichment analyses of IGSP high scoring genes revealed canonical pathways associated with CHD risk (**Fig 3A** and **S6 Table**). Connecting such high scoring genes based on the characteristics of the CHD could potentially reveal novel underlying risk pathways. Transcriptional regulation is known to play a major role in heart development, and abnormalities in several transcription factor (TF) pathways are closely linked to CHD risk [38]. By leveraging the prior knowledge of interactions between TFs and their target genes, we sought to uncover the TF pathways underlying risk of syndromic CHD in 22q11.2DS. Based on putative TF-target interactions from TRRUST [39] and GSEA (the gene set of transcription factor targets) [40], we constructed a regulatory network of TFs and their targets from among the 200 top IGSP high scoring genes for CHD in 22q11.2DS. We identified a subnetwork enriched with 63 of these top 200 high scoring genes (**Fig 4**). This network includes multiple key CHD TF risk genes (*NKX2-5*, *GATA4*, *MEF2C*, and *SRF* [38]) and reveals their downstream pathways for CHD development. For example, TF targets in this subnetwork are involved in multiple biological processes important for heart development, including apoptotic process, ERBB signaling pathway, cell cycle regulation, focal adhesion, BMP signaling pathway, and WNT signaling pathway [41]. Among 63 genes in the network, VEGFA has been suggested as a modifier gene of CHD in 22q11.2DS [42]. Other potential modifier genes of CHD in 22q11.2DS found in the network are genes perturbed by *TBX1* haploinsufficiency implicated in the mouse model (*GATA4*, *T Brachyury*, *CSRP3*, *KIF1B*, *CYR61*, and *WNT5A*). The key CHD TF risk genes and potential modifier genes of CHD in 22q11.2DS are well organized in a transcriptional regulatory network, revealing the potential mechanisms of CHD risk modification in 22q11.2DS and also supporting, from the methodological point of view, the effectiveness of integrated scoring of IGSP.

### The required sampling steps and convergence time of IGSP

Computational feasibility is an important factor for risk gene prioritization from a practical perspective. Using the CHD WES data in the case study and the SCZ simulated data as examples, IGSP reached convergence within about several thousand sampling steps, which took several minutes in general (**S8 Table**). A setting with larger *x* corresponded to a larger number of possible combinations of risk genes (i.e. the number of states in Markov chain) (assume *x*< 50) and thus might require more sampling steps to reach convergence. Overall, IGSP is computationally efficient and is computationally feasible to conduct intensive negative control analysis.

### Practical consideration: The choices of parameters

In IGSP, the parameter *x* is used to determine the number of sampling risk genes in a stochastic process to infer risk-gene likelihood. A practical question is how to select *x* when using IGSP to study a disease. Our simulation showed that IGSP performed better when *x* was set close to the real value (**S4 Fig**) and the performance was largely unchanged when *x* was set within a reasonable range of its real value. A minimal number of sampling risk genes (e.g., 30 genes) is required for IGSP to detect and utilize network and phenotype properties of possible risk genes. In most simulation cases, IGSP performed equally well with 30 sampling risk genes (minimal *x*) or *x* close to the real value (**S14 Fig**). While a sample size of 30 genes in our approach can effectively characterize the statistical properties of possible risk genes, selecting *x* corresponding to the real value has the advantage that final scores can also be interpreted as the probabilities of risk genes. For some diseases, *x* can be calculated based on the widely accepted estimated number of risk genes. For other diseases, it may be empirically predicted from the sequencing data (**S15 Fig**).

The parameters *a* and *b* determine the scaling range of association signals in a process of signal adjustment to integrate association signals with network and phenotype evidence. Iterative signal adjustment in a scaling range makes it possible for genes with genotype evidence to stand out from association signals of false positives in the process of signal adjustment. The scaling range should be kept small with a lower bound (*a*) greater than 0 to avoid loss of association signals. For example, when (*a*, *b*) = (0.1, 1), all genes with *P*-values < 0.5 can compete with one another, including genes with *P*-values < 1E-6 (According to Eq 1, the highest possible integrated scores for genes with a *P*-value of 0.5 and the lowest possible integrated scores for genes with a *P*-value of 1E-6 are roughly the same). This setting of (*a*, *b*) allows genes with even tiny genotype evidence to compete with genes with genome-wide significant disease associations (stand out from potential false positives) and thus provides a proper scaling range that can be applied to all sequencing data. We also tested values of *a* and *b* specially tuned for the sequencing data: *a* = 0.9999999 (≈ 1) and *b* = (the highest association score in the sequencing dataset /–log_10_(0.5))– 1. Under this setting all genes with *P*-values lower than 0.5 can compete with genes with the lowest *P*-values in the dataset. The performance with this special setting is similar to or slightly better than that with the generalized setting (*a*, *b*) = (0.1, 1) in our simulation (**S14 Fig**). In summary, users can start their analysis with the generalized setting (*a*, *b*) = (0.1, 1) as default and may tune these parameters for potentially better performance.

## Discussion

In this study, we address the problem of insufficient statistical power in association sequencing studies through data integration, a method which has been successfully employed previously for risk gene prioritization [43, 44]. Instead of solely relying on association signals, our IGSP approach integrates this information with gene network and phenotype data to allow optimal detection of putatively bona fide disease association signals of rare variants at the gene level and thus maximize the likelihood of uncovering weak (effect size) but real disease risk genes within a dataset.

Integration of orthogonal data provides great potentials to improve power of risk gene prioritization in genetic studies. It is a challenge, however, to design a data integration method with proper model specification. The reliance on known risk genes or known disease phenotypes in prevalent integration methods raises potential concern of model misspecification. First, the prior knowledge of diseases regarding their risk genes and phenotypes is likely biased toward well studied pathways and most observable phenotypes. Furthermore, because diseases could have different subtypes, previously identified disease risk genes or phenotypes might pertain to specific disease subtypes and do not hold for diseases under investigation. Under either circumstance, the model may be misspecified which results in power loss. Based on an integration strategy without relying on prior disease knowledge, our method thus reduces risk of model misspecification. The fundamental feature of our model design is that the likelihood of risk genes is evaluated based on not only their association signals but their network and phenotype relationship with other possible risk genes. Compared with the risk gene prioritization method simply based on individual association signals of genes alone, IGSP gains additional predictive power by leveraging the power of association signals of other possible risk genes. The effect of IGSP is thus constrained by the constructed network and phenotype relationship among genes. In our case study, there are genes with relative strong association signals but ranked after hundreds, such as CD109 and ZNF142, due to weak network and phenotype evidence. Among those genes we didn’t find any known CHD risk genes. However, the possibilities that certain true risk genes with relatively strong association signals are scored low by our method due to incomplete knowledge of network and phenotype relationship among genes cannot be excluded.

Our approach prioritizes risk genes through integrated analysis of gene association signals. The rare variant association test and the rare variants selected for the association test can have a significant impact on the test *P*-values and thus directly affect the performance of IGSP. Currently, however, we do not have well-established criteria for both choices, which depend on the genetic architecture of the disease under investigation. On the other hand, functional prediction of variants has been frequently applied to improve the power of association tests by weighting or focusing on variants that are predicted to be deleterious [45]. We used a stringent selection of rare variants and the burden test of disease association for two reasons. First, a stringent threshold on the Combined Annotation Dependent Depletion (CADD) score retains only rare variants that are predicted to be highly deleterious to gene function and thus more likely to be causal. Under such circumstances, it has been shown that a burden test is more powerful if most variants under the test are causal [46, 47]. Second, by focusing on highly deleterious variants and with the simple null hypothesis of burden tests that only compares the collective variant burden, it is relatively straightforward to interpret gene association *P*-values with convincing genetic evidence (compared with SKAT [48], for example). The disadvantage of selecting most deleterious variants is a loss of information about less-deleterious causal variants, which may diminish the opportunities of identifying strong gene association. In our IGSP approach, however, the quality of genotype-based scoring depends on the strength of association signals from coding variants in risk genes relative to these in non-risks genes. The absolute value of variant association strength does not play a role.

Simulations showed that our IGSP approach can significantly improve risk gene prioritization for rare variant association studies under different configurations of association test results. In real applications, assessing the scoring result needs additional consideration. For example, the statistical power of rare variant association tests can be extremely poor such that applying IGSP may not lead to meaningful improvement of risk gene prioritization. In addition, IGSP will only work for polygenic diseases with data support from the co-function network and MP phenotypes. One way to evaluate the result is gene-set analysis. The high scoring genes of syndromic CHD in 22q11.2DS were overrepresented in GO terms related to heart development. Furthermore, the mouse homologs of high-scoring genes were enriched in the downstream pathways of *Tbx1* (**Fig 3**), which supports the hypothesis that haploinsufficiency of *TBX1* contributes to the increased CHD susceptibility of 22q11.2DS patients. Moreover, top-ranking genes were scored significantly higher with actual association signals than with null signals (**S13E Fig**), which suggests that genes sharing network and phenotype similarities provide stronger rare variant association signals in a given cohort. This negative control feature built into IGSP is another way to assess the IGSP gene scoring results. When investigating a novel trait, the negative control analysis is especially useful since the result evaluation based on gene-set analysis may not be viable.

In our simulation and case study, we focused on genes that can be scored with both co-function gene network and mouse knockout phenotype information. Our simulations showed that mouse phenotypes provide a powerful feature to discriminate genes for human disease risk. The application of MGI phenotype annotation is limited, however, by its relatively low coverage of genes: currently only about half of human protein-coding genes have mouse orthologs with phenotype information in the MGI database. The network scoring, on the other hand, covers greatly more protein-coding genes in the human genome. Amongst 12,196 genes with association signals in the CHD study, 11,840 could be scored with network information. GO term enrichment analysis indicates that, compared with scoring by the association signal alone, IGSP with only network integration is effective but not as powerful as a full integration that also includes mouse phenotype scoring (**S9 & S10 Tables**). Nevertheless, top high-scoring genes from IGSP with network integration alone could be used to compensate for the gene coverage deficiency of using both network and phenotype.

In addition to key CHD risk genes, IGSP identified several putative risk genes not found in the previous association study of the same sequencing data that used a candidate gene approach [18]. Some of these new putative risk genes have been linked to CHD risk in previous studies, such as *NPHP4* [49] (nephronophthisis-4) and *NRP1* [50] (neuropilin 1). Most of these putative risk genes have weak *P*-values and come from diverse pathways. Without integrating other information such as gene network and phenotypes, it is extremely challenging to bring them to light, and being able to do so more optimally represents the main benefit of our method. By focusing on high-scoring genes without a rare predicted deleterious variant in control, we curated a list of potential modifier genes and variants for CHD in 22q11.2DS for future experimental validation (**S11 Table**). On the other hand, it is important to highlight differences between the published study [18] and our new study. First, and possibly most importantly, two studies used different criteria to select rare deleterious variants. The previous study [18] examined a wider range of deleterious rare variants, aiming to find modifiers predicted to be damaging when combined with the deletion. In this study, we focused on the most deleterious rare variants to allow generation of the clearest disease association signals. Risk genes implicated by the previous study did not have particularly stronger association signals in our study possibly due to that many chromatin modifier variants are not extremely deleterious. Second, the previous study only incorporated evidence for the presence of rare predicted deleterious variants, whereas we took advantage of additional bioinformatic methods for risk gene discovery. Indeed, because there are likely many CHD risk and/or protective genes within the cohort, different approaches, such as these are needed to gain a fuller understanding of the roles of such genes in CHD of 22q11.2DS.

In summary, we developed IGSP, a rare-variant based integrated approach to prioritize risk genes in sequencing-based association studies. The design of the method addresses the issue of insufficient statistical power and can significantly improve the identification of disease risk genes implicated by rare variants with marginal association signals. By expediting such discoveries, it can shed light on the roles of rare variants in human complex diseases.

## Methods

The workflow of IGSP can be divided into two parts (**S16 Fig**): gene scoring based on genotype, network, and phenotype information and a model-based score integration, followed by a Markov chain Monte Carlo (MCMC) algorithm to approximate risk gene probabilities as final scores.

### Genotype-based scoring

Our method starts with the genotype-based scoring of genes for their disease association using a rare variant association test (**S16 Fig**). Given the full set of variants called in the WGS or WES data from case and control samples, we first select rare variants whose alternative allele frequencies are less than 1% in both the 1000 Genomes Project Europe samples and the study cohort. Among such rare variants, we then use Combined Annotation Dependent Depletion (CADD) [51], which scores the deleteriousness of variants by combining multiple types of functional annotation, to select predicted deleterious variants. The cutoff of CADD scaled scores on deleteriousness is suggested to be between 10 and 20. We applied a stringent cutoff, 20, that keeps top 1% deleteriousness of SNVs in humans [51]. For downstream analysis, a set of rare variants with alternative allele frequencies < 1% and CADD scaled scores ≥ 20 are selected. We aggregated these rare variants into sets corresponding to protein-coding genes, and then used the kernel-regression burden test [47] to assess the disease association of rare variants at the gene level. The nominal *P*-values of association tests are converted to genotype-based scores by taking minus log10.

### Phenotype-based scoring

Genes involved in the same biological process are more likely to influence similar phenotypes [52] and thus tend to be associated with the same or similar diseases **(Fig 1B and S2 Fig)**. This characteristic is captured by the phenotype-based scoring of genes in our method. We extracted the phenotype features of genes using the mouse/human orthology with phenotype annotations from the Mouse Genome Informatics (MGI, http://www.informatics.jax.org, data downloaded on April 19, 2017) (**S16 Fig**). 9,198 human homologs of mouse genes have at least one MP term in this MGI data set. We consider 9,570 MP terms including 8,865 directly annotated MP terms (for the 9,198 human homologs) and 705 their ancestor MP terms along the paths of the 'is a' relationship in the MP hierarchy structure. For the 9,198 human homologs associated with 9,570 MP terms, we first built a binary matrix that encoded their association (1 if present and 0 otherwise). We then computed the principal components of genes in the feature space of the phenotype terms and used the eigenvalues of the *n* principal components as Eigenfeatures to characterize the phenotype features of all human genes. These genes were divided into two classes: disease risk genes and non-risk genes (the rest). Given the binary column vector **v** of risk gene status, we use logistic regression to regress the class label on the phenotype eigenvalues (*x*_1_, *x*_2_, …*x*_*n*_) of genes and obtain their regression coefficients (*β*_0_, *β*_1_, *β*_2_, …*β*_*n*_) with the logit function:

$$\mathrm{logit}(\mu )=\beta_{0}+\beta_{1}x_{1}+\beta_{2}x_{2}+{\ldots \;\beta }_{n}x_{n}$$

Every gene has a probability (*μ*) based on the trained logistic model. The probabilities are the raw phenotype gene scores (**S17 Fig**). We used the same normalization method for network-based scoring to transform raw phenotype into normalized scores with range of [0, 1] (see below).

### Network-based scoring

Gene networks describe complex functional relationships among genes in which genes causing the same or similar diseases tend to lie close to one another regarding function [53, 54] **(Fig 1B and S1 Fig)**. This topological propensity is captured by the network-based scoring of genes in our method. For this scoring, several different networks of either protein-protein interactions [55] or gene functional linkages [56] can be used. Currently, we use a recently published co-function network [17], in which two genes are connected if they are predicted to share specific functions. We made this selection because this network not only covers a high percentage (~94%) of human genes but also, more importantly, consists of only gene-gene co-functional connections predicted from genomic high-throughput data [17] and thus avoids the ascertainment bias toward well-studied genes.

Given a gene network, network-based scoring measured the connectivity propensity of possible risk genes to a scoring gene. Here we measured the connectivity propensity of possible risk genes based on a transition matrix that considered the degrees of possible risk genes. The way of measuring the connectivity propensity simply based on the number of connections worked as well (See Results section). Given the gene network *G* = (*V*, *E*) with its ensemble of *n* nodes, *V*, and edges, *E*, we built its corresponding adjacency matrix **A** with *a*_*ij*_ = *w*_*ij*_ if *i* ↔ *j ϵ E* or 0 otherwise. *w*_*ij*_ is the weight of the edge between nodes *i* and *j*. We normalized **A** by columns and obtain the transition matrix **N**. Given the binary column vector **v** of risk gene status, the raw network-based scores were calculated as (**S18 Fig**):

$$r^{\left(N\right)}=N∙v.$$

These raw scores were normalized to get the network-based scores in the range of [0, 1] by calculating the rate of risk genes with raw network scores lower than the raw network score (denoted by normalization *f*_*n*_ function):

$$s^{\left(N\right)}=f_{n}(r^{\left(N\right)})$$

This normalization method is the same for the normalization of raw phenotype scores. The normalization of raw scores is necessary to transform different types of network and phenotype evidence (i.e., raw network/phenotype scores) into a unified range that meets our model specification. We applied a specialized normalization method (f_n_) that utilized the information of sampled risk genes; it considers the distribution of raw network and phenotype scores of sampled risk genes as the baseline in assessing/converting raw network and phenotype scores. Other normalization methods that can scale raw scores from 0 to 1 may also work as well (See Results).

### Score integration

We integrate the aforementioned genotype-, network-, and phenotype-based gene scores according to the following formula:

$$s=s^{\left(A\right)}∙[I+b∙s^{\left(N\right)}∙s^{\left(P\right)}]$$

$$I=\left\{ \begin{array}{l} \begin{array}{cc} 1 & \mathrm{if}\;g\;\mathrm{is}\;a\;\mathrm{risk}\;\mathrm{gene} \end{array}\\ \begin{array}{cc} a & \mathrm{otherwise} \end{array} \end{array}\;,\right.\;(0<a<1)$$ (1)

*s*^(*A*)^,*s*^(*N*)^, and *s*^(*P*)^ represent the association, network and phenotype of a gene, *g*, respectively. The indicator variable *I* has two effects on score integration. First, it establishes a lower-bound on the scaling coefficient and thus prevents domination of the final score by the gene network and phenotype components. Second, it diminishes signals from genes that have a low probability to be risk genes. According to the Eq 1, the integrated score *s* of a gene is essentially its disease association signal scaled by a multiplication coefficient based on network and phenotype evidence. The value of the coefficient ranges between 0.1 and 2. This range of scaling was fine-tuned to optimize the method performance by simulation (*a* = 0.1 and *b* = 1). If the scaling range is too small, there is little room for improvement to risk gene prioritization. On the other hand, if the scaling range is too large, there is a loss of association signals, which will also lead to poor estimation.

By design of Eq 1, IGSP essentially assigns to each gene a range for association signal adjustment based and centered on its original disease association signal (**S19 Fig**). Adjusted signals are integrated scores vary within the corresponding ranges depending on their network and phenotype evidence. Given association signals, our scoring model decides a multivariate probability distribution of integrated scores within the ranges. Our gene scoring is based on this multivariate probability distribution of integrated scores.

### Probability derivation and why MCMC

Given *m* scoring genes, assume that *x*% of scoring genes are risk genes, there are about *n* = round (*m* × *x*%) risk genes. Let *d* denote a combination of risk genes, *d* ∈ *D*. The number of possible *d* is: $|D|=\left(\begin{array}{c} m\\ n \end{array}\right)$. Given association signals ***s***^(*A*)^, the probability of a gene being a risk gene is:

$$P(g|s^{\left(A\right)})=\sum_{d\in D_{g}}P(d|s^{\left(A\right)})$$ (2)

$$=\sum_{d\in D_{g}}\sum_{s_{i}}P(d|s_{i})\times P(s_{i}|s^{\left(A\right)})$$ (3)

where *D*_*g*_ is the subset of *D* that includes gene *g* in the combination. It should be noted that there are only a limit number of outcomes of integrated scores since each combination of risk genes determines an outcome of integrated scores (Eq 1) while there are $\left(\begin{array}{c} m\\ n \end{array}\right)$ combinations of risk genes. Therefore, we can derive Eq 3 since each combination of integrated scores can be considered as a disjoint event, and the sum of their probabilities is equal to 1. Since integrated score *s* evaluates the likelihood of being a risk gene in our model, the probability *P*(*d*|***s***_*i*_) in Eq 3 is equivalent to the probability of obtaining *d* through weighted sampling n out of m genes without replacement, using ***s***_*i*_ as weight. However, the challenge is to calculate *P*(***s***|***s***^(*A*)^) in Eq 3. According to our integrated model (Eq 1), *P*(***s***|***s***^(*A*)^) can be derived as follows:

$$P(s|s^{\left(A\right)})=P(I+b∙s^{\left(N\right)}\circ s^{\left(P\right)}=s⌀s^{\left(A\right)})$$ (4)

$$=\sum_{d\in Q}P(d|s^{\left(A\right)})$$ (5)

where ∘ and ⌀ denotes component-wise multiplication and component-wise division, respectively. The probability *P*(***s***|***s***^(*A*)^) is essentially the probability that the vector of scaling factors (***I*** + *b* ∙ ***s***^(*N*)^ ∘ ***s***^(*P*)^) is equal to ***s***⌀***s***^(*A*)^. As scaling factors are determined by *d* and the prior knowledge of network and phenotype, if *Q* denotes the set of possible *d* of which the corresponding vector of scaling factors is equal to ***s***⌀***s***^(*A*)^, we can derive Eq 5. Unfortunately, it cannot be directly calculated since it forms an infinite recursive loop (Eq 2).

### Final score calculation

To tackle the challenge of deriving risk gene probabilities, instead of calculating the probability *P*(***s***|***s***^(*A*)^), we develop an MCMC-based algorithm to directly sample the probability function $\sum_{s_{i}}P(d|s_{i})\times P(s_{i}|s^{\left(A\right)})$ in Eq 3 while approximating risk gene probabilities of all genes, concurrently, according to Eq 3 as their final scores (**S20** & **S21 Figs**):

1. *Initialization*
  1.1 Start with random *x*% of genes as sampled risk genes
2. *Repeat until convergence*
  2.1 Score every gene based on sampled risk genes according to the Eq (1)
  2.2 Randomly select *x*% of genes as new sampled risk genes with sampling probabilities proportional to their gene scores
  2.3 Calculate the sampling rate of every gene

The algorithm constructs a Markov chain (**S20 Fig**) and samples the probabilities by simulating a random walk on the Markov chain to generate final scores. It essentially approximates the risk gene probability of a gene by calculating the probabilities of visiting states that imbed a combination of risk genes including the gene. It starts to calculate sampling rates after a 'burn-in' period of 1,000 iterations and records the rates every 1,000 iterations. Convergence is deemed reached if the average difference of the sampling rates of all genes between consecutive records is less than 0.001.

### Data simulation and method evaluation

We simulated disease the association signals of genes and systematically evaluated and compared the performance of IGSP and the widely-used rare variant association test in disease risk gene prioritization. For a thorough performance evaluation, we used the 8,959 human protein-coding genes for which both network and phenotype information was available to us in our data simulation. Given a disease, to simulate the association signals, we first divided these genes into two categories: disease risk genes and non-risk genes. The former were collected from disease gene resources such as MelaCards [57]. Disease association signals of disease risk genes and non-risk genes were simulated separately. For non-risk genes, we simulated their gene association signals with a *P-*value uniformly distributed between 0 and 1 [58]. For risk genes, on the other hand, the distribution of their association *P-*values should be skewed toward 0, with the degree of skew depending on the power of the study. We thus simulated association signals of disease risk genes by assigning them smaller *P*-values (**S4 Table**). In each configuration of simulation, a different proportion of disease risk genes were assigned smaller *P*-values, representing studies with different statistical power. The proportion and the statistical power were positively correlated.

### WES data for CHD of 22q11DS

The WES data that we reanalyzed have been described in detail in the original association study of CHD in 22q11.2DS [18]. Briefly, the study cohort consists of 184 22q11.2DS patients. Among them, 90 patients having CHD (mostly tetralogy of Fallot) were cases, and the remaining 94 having a normal heart and aortic arch were controls. 411,618 variants (SNVs and indels) were identified by WES of this cohort. Among the 398,808 variants that passed quality control (variants with missing genotype rate > 10% were removed), we retained in coding sequence 61,220 rare variants (alternative allele frequencies < 1% based on EUR samples in the 1000 Genomes Project and the study cohort) with predicted deleterious alternative alleles (CADD score >= 20). We conducted burden tests to obtain association *P*-values of genes that had at least one rare predicted deleterious SNV in the cohort. Burden tests for genes on chromosome X were adjusted for sex by including this variable as covariates in the analysis.

### Software and data availability

IGSP is freely available for academic use as a web application at http://zdzlab.einstein.yu.edu/1/igsp.html. The web application requires only a gene list with corresponding association *P*-values. The source codes of the IGSP method and obtaining gene association *P*-values from the CHD sequencing data are provided at Zenodo (10.5281/zenodo.1034362 and 10.5281/zenodo.1034177, respectively).

## Supporting information

S1 Fig The network property of disease risk genes. Red arrows point to the number of connections among risk genes for the disease in the co-function network [17]. A null distribution is constructed by counting the number of connections among random genes (10000 iterations) with the same number of disease risk genes in the network. The risk genes of different diseases are listed in **S1 Table**. (A) Breast cancer. (B) Schizophrenia. (C) Tetralogy of Fallot. (D) Systemic lupus erythematosus. (E) Type 2 Diabetes.
(DOCX)

S2 Fig The phenotype property of disease risk genes. There is a tendency that disease risk genes tend to cluster on top principal components of MP phenotypes. The first principal component is excluded from consideration since it mainly characterizes the number of annotated MP terms (correlation coefficient = 0.918), which is not biological meaningful. The risk genes of different diseases are listed in **S1 Table**. (A) Disease risk genes on second, third, and forth principal components. (B) Disease risk genes on principal component 2 to 5. (C) Disease risk genes on principal component 6 to 9.
(DOCX)

S3 Fig Genotype-based scoring has no relationship with co-function network degree and the number of associated MP terms. Using the CHD case-control WES data in our case study, we collected 1,000 sets of null gene association signals. Each null set was obtained from the burden test used in our method by permuting the original disease status label. We then tested whether there’s a relationship between genotype-based scoring and the co-function network degree or the number of associated MP terms. The relationship between genotype-based scoring and the co-function network degree was evaluated based on genes with both association scores and network degrees. The relationship between genotype-based scoring and the number of associated MP terms was evaluated based on genes with both association scores and phenotype scores (i.e. genes with at least one associated MP terms). (A) Correlation coefficient. The median correlation coefficients were 0.0067 and 0.0082 between the association score and the co-function network degree ('G and N') and between the association score and the number of associated MP terms ('G and P'), respectively. (B) Correlation *P*-value. The median *P*-values were 0.469 and 0.523 for the correlation between the association score and the co-function network degree and the correlation between the association score and the number of associated MP terms, respectively.
(DOCX)

S4 Fig The performance of IGSP with different *x*. The number of risk genes in the top 50 high scoring genes (y axis) is used to evaluate the performance. The setup of the simulation: integrated scoring with both network and phenotype, “Moderate” association signal strength, *a* (0.1), *b* (1), principal components in phenotype scoring (PC 2 and 3). (A) 147 CHD genes (B) 193 SCZ genes.
(DOCX)

S5 Fig The performance of IGSP with different *a*. The number of risk genes in the top 50 high scoring genes (y axis) is used to evaluate the performance. The setup of the simulation: integrated scoring with both network and phenotype, “Moderate” association signal strength, *x* (2), *b* (1), and principal components in phenotype scoring (PC 2 and 3). (A) 147 CHD genes (B) 193 SCZ genes.
(DOCX)

S6 Fig The performance of IGSP with different *b*. The number of risk genes in the top 50 high scoring genes (y axis) is used to evaluate the performance. The setup of the simulation: integrated scoring with both network and phenotype, “Moderate” association signal strength, *x* (2), *a* (0.1), and principal components in phenotype scoring (PC 2 and 3). (A) 147 CHD genes (B) 193 SCZ genes.
(DOCX)

S7 Fig The performance of IGSP with different top principal components in phenotype scoring. The number of risk genes in the top 50 high scoring genes (y axis) is used to evaluate the performance. The setup of the simulation: integrated scoring with phenotype information, “Moderate” association signal strength, *x* (2), *a* (0.1), and b (1). (A) 147 CHD genes (B) 193 SCZ genes.
(DOCX)

S8 Fig The distribution of network and phenotype scores. The distribution of scores was constructed based on the network or phenotype scores of IGSP-scoring genes at the 3000^th^ iteration of a trial in CHD simulation. 'S' denotes sampled risk genes while 'B' denotes the other background genes at that iteration. The setup of the simulation: integrated scoring with both network and phenotype, 'Moderate' association signal strength, *x* = 2, *a* = 0.1, *b* = 1, and principal components in phenotype scoring (PC 2 and 3). (A) The raw network and phenotype scores. (B) The network and phenotype scores after normalization.
(DOCX)

S9 Fig Normalization of network and phenotype raw scores is a necessary step for IGSP. The result showed that IGSP using raw network and phenotype scores without normalization barely improved upon gene association signals. 'Gen + Net' and 'Gen + Phe' represent IGSP with only integration of network features and phenotype features, respectively. The parameters used in this simulation were as follows: *x* = 2, *a* = 0.1, *b* = 1, and principal components in phenotype scoring (PC 2 and 3). (A) CHD. 147 CHD genes from Sifrim et al [26] were used as the risk genes. (B) Schizophrenia. 193 putative schizophrenia genes from MalaCards [57] were used as the risk genes.
(DOCX)

S10 Fig IGSP works with different normalization methods for raw score normalization. The dashed line represents IGSP results using a different raw-score normalization method: (score–min (scores)) / max(scores)–min(scores)). 'Gen + Net' and 'Gen + Phe' represent IGSP with only integration of network features and phenotype features, respectively. The parameters used in this simulation were as follows: *x* = 2, *a* = 0.1, *b* = 1, and principal components in phenotype scoring (PC 2 and 3). (A) CHD. 147 CHD genes from Sifrim et al [26] were used as the risk genes. (B) Schizophrenia. 193 putative schizophrenia genes from MalaCards [57] were used as the risk genes.
(DOCX)

S11 Fig The performance of IGSP based on different types of network connectivity in network-based scoring. 'Gen + Net' represents IGSP with only integration of network features. We compared the performance of using two different types of network connectivity (between a scoring gene and possible risk genes) in network-based scoring. C1 represents the network connectivity based on a transition matrix (considering network degree). C2 represents the network connectivity based on an adjacency matrix (without considering network degree). The parameters used in this simulation were as follows: *x* = 2, *a* = 0.1, *b* = 1, and principal components in phenotype scoring (PC 2 and 3). (A) CHD. 147 CHD genes from Sifrim et al [26] were used as the risk genes. (B) Schizophrenia. 193 putative schizophrenia genes from MalaCards [57] were used as the risk genes.
(DOCX)

S12 Fig The performance evaluation of using different integrated models. The performance of using our current integrated model (Eq 1) was compared with that of using four other different integrated models; full integration of network and phenotype features was applied in performance evaluation. The parameters used in this simulation were as follows: *x* = 2, *a* = 0.1, and principal components in phenotype scoring (PC 2 and 3). *b* was set as 1 for the current integrated model but as an indicator variable defined below. (A) Definition of four alternative integrated models. In these models, the weight of network and phenotype components, *b*, is set as an indicator variable dependent on risk gene status. (B) CHD. 147 CHD genes from Sifrim et al [26] were used as the risk genes. (C) Schizophrenia. 193 putative schizophrenia genes from MalaCards [57] were used as the risk genes.
(DOCX)

S13 Fig Distribution of top IGSP scores given association signals in null as negative control. Association signals in null are gene association signals of which stronger signals do not tend to aggregate at risk genes. Association signals in null in simulation (A, B, C and D) are obtained through randomly assigning simulated gene association signals of all genes, including risk and non-risk genes, to themselves. Association signals in null in a real application (E) are obtained through permuting the original disease status label and randomly assigning the resultant disease association signals to genes. The scores of top 100 high scoring genes in IGSP are shown in the figures. 100 trials of IGSP given association signals in null are conducted and the error bars represent standard deviation. The parameter setup of IGSP for simulation in A, B, C and D and the real application in E: integrated scoring with both network and phenotype, *x* (2), *a* (0.1), *b* (1), and principal components in phenotype scoring (PC 2 and 3). (A) Simulation with 147 CHD genes with a setup of “Very weak” association signal strength. (B) Simulation with 147 CHD genes with a setup of “Weak” association signal strength. (C) Simulation with 147 CHD genes with a setup of “Moderate” association signal strength. (D) Simulation with 147 CHD genes with a setup of “Strong” association signal strength. (E) A real application in the case study of CHD.
(DOCX)

S14 Fig The performance of IGSP with 30 sampling risk genes and a specialized scaling range. The performance of IGSP using a minimal number of sampling risk genes and a specialized scaling range is compared with that of IGSP using the optimized setting of *x*, *a*, and *b*. Full integration of network and phenotype features was applied in performance evaluation. The parameters used in this simulation were shown in figures and as follows: principal components in phenotype scoring (PC 2 and 3). 'Minimal' represents *x* approximating 30 sampling risk genes; 'Optimized' represents (*a*, *b*) = (0.1, 1); 'Specialized' represents a setting of *a* and *b* in which *a* is set as 0.9999999 (≈ 1) and *b* is set as (the highest association score in the sequencing dataset /–log_10_(0.5))– 1. (A) CHD. 147 CHD genes from Sifrim et al [26] were used as the risk genes. (B) Schizophrenia. 193 putative schizophrenia genes from MalaCards [57] were used as the risk genes.
(DOCX)

S15 Fig Prediction of *x* from the CHD sequencing data. Genes with stronger association signals (i.e., with smaller *P*-values) are more likely to be risk genes. This method predicts *x* based on the difference between the strength of disease association and null association against the corresponding difference when all genes have robust disease association and thus are risk genes. Association signals of CHD sequencing data were obtained from the burden test used in our method. Null association signals were obtained from the burden test used in our method by permuting the original disease status label. We predicted a range of *x* based on two different metrics in measuring gene association strength. (A)–log_10_*P*. For 5,987 scoring genes, the sum of disease association and the average sum of null association for 1,000 sets of null association signals were 2512.0 and 2380.1, respectively. The (minimum) sum of robust disease association was calculated based on a scenario when each scoring gene has a *P*-value surviving multiple correction: (–log_10_(0.05/5987)×5987) = 30403.2. We predicted *x* = 0.43 percent of scoring genes associated with the disease in this sequencing data based on a linear interpolation. Compared with the other metric in measuring association strength, this metric can better estimate the number of risk genes with strong association but may underestimate the number of risk genes with weak association. (B) 1–*P*. For 5,987 scoring genes, the sum of disease association and the average sum of null association for 1,000 sets of null association signals were 3098.2 and 2994.8, respectively. The sum of robust disease association was calculated based on a scenario when each scoring gene has a *P*-value surviving multiple correction: (1–0.05/5987) × 5987 ≈ 5987. We predicted *x* = 3.4 percent of scoring genes associated with the disease in this sequencing data based on a linear interpolation.
(DOCX)

S16 Fig The workflow of IGSP. The workflow of IGSP can be divided into two parts: The scoring model of risk genes and calculation of final gene scores. Given association signals ***s***^(*A*)^, the scoring model will determine a multivariate probability distribution of integrated scores (***s***) (bold denotes vectors). In final score calculation, we applied Markov chain Monte Carlo (MCMC) in our algorithm to approximate risk gene probabilities as final gene scores according to the multivariate probability distribution of integrated scores.
(DOCX)

S17 Fig Phenotype-based scoring. Principal components from 9,198 human homologs and 9,570 MP terms are used to characterize the phenotype features of each gene. Genes with a higher propensity to share phenotype similarities with risk genes have higher phenotype scores. The combination of risk genes is the same latent variable shown in network-based scoring (**S18 Fig**).
(DOCX)

S18 Fig Network-based scoring. Genes with a higher propensity to be connected to risk genes have higher network scores. A and B can be risk genes themselves and their network scores are calculated in the same manner. The risk gene vector depends on the combination of risk genes (*d*), which is a latent variable in our model.
(DOCX)

S19 Fig Integrated scores as adjusted signals in IGSP. G1 is a risk gene, while G2 and G3 are two non-risk genes that have a stronger association signal than G1. (A) Gene association signals. Gene association signals are obtained through gene association tests on the investigated sequencing data. In the figure, a red cross represents a risk gene, while a grey dot represents a non-risk gene. Association signals of risk genes are buried in noise. (B) Signal adjustment by linear scaling. IGSP integrates association signals with network and phenotype evidence by multiplying a scaling coefficient to original gene association signals. The scaling coefficient of each gene incorporates its network and phenotype information, as shown in Eq 1. Every gene has the same scaling range. (C) The adjustment ranges of different genes. While the scaling ranges of all genes are the same, the adjustment range of each gene depends on the magnitude of its original signal. The final score of each gene is calculated as its risk gene probability based on the multivariate probability distribution of adjusted signals within the adjustment ranges determined by Eq 1.
(DOCX)

S20 FigThe underlying Markov chain of IGSP.Each combination of risk gene determines a state. The number of states in this Markov chain is the number of possible combination of risk genes. Given association signals, a combination of risk genes determines integrated gene scores according to Eq 1; the transition probability determining progression to the next state is equal to the weighted sampling n from m genes without replacement, using ***s*** as weight. Instead of calculating the transition probability, our MCMC-based algorithm carries out state transition using the MATLAB “datasample” function with integrated scores ***s*** as weight. The Markov chain is ergodic (irreducible and aperiodic) and hence has only a single equilibrium distribution. When equilibrium is reached, the probability of visiting a state (*d*) approximates $\sum_{s_{i}}P(d|s_{i})\times P(s_{i}|s^{\left(A\right)})$ in Eq 3.(DOCX)

S21 Fig The probability landscape and MCMC sampling of IGSP. The upper plot shows the probability landscape of different combinations of risk genes, *d*, given association gene signals ***s***^(*A*)^. In the upper plot, each point on the plan represents a combination of risk genes. The redness in the bottom plot represents the frequency of visiting the corresponding states (see **S20 Fig**).
(DOCX)

S1 Table Risk genes of 5 human diseases from OMIM. (DOCX)

S2 Table 147 putative CHD genes that can be scored by network and phenotype. Those genes were obtained from Sifrim et al [26].
(DOCX)

S3 Table 193 putative schizophrenia genes that can be scored by network and phenotype. Those genes were obtained from Malacards [57].
(DOCX)

S4 Table The setup of *P*-values of risk genes in simulation. (DOCX)

S5 Table Top 200 genes based on IGSP integrated scoring for the case-control WES study of CHD in 22q11.2DS. (DOCX)

S6 Table Enriched biological process GO terms for top 50 genes based on IGSP integrated scoring for CHD. This result is for 5987 genes in the CHD dataset with association signals of rare predicted deleterious variants that can be scored by network and phenotype.
(DOCX)

S7 Table Top 10 biological process GO terms for top 50 genes based on association *P*-values from burden test for CHD. This result is for 5987 genes in the CHD dataset with association signals of rare predicted deleterious variants that can be scored by network and phenotype.
(DOCX)

S8 Table The required sampling steps and convergence time of IGSP. (DOCX)

S9 Table Enriched biological process GO terms for top 100 genes based on IGSP network-based scoring for CHD. This result is for 11840 genes in the CHD dataset with association signals of rare predicted deleterious variants that can be scored by network.
(DOCX)

S10 Table Top 10 biological process GO terms for top 100 genes based on association *P*-values from burden test for CHD. This result is for 11840 genes in the CHD dataset with association signals of rare predicted deleterious variants that can be scored by network.
(DOCX)

S11 Table Curated genetic modifier genes and variants of CHD in 22q11.2DS for future validation. (DOCX)
