# Supplementary figures and images for: Integrated rare variant-based risk gene prioritization in disease case-control sequencing studies
Source: PLoS Genet. 2017 Dec 27;13(12):e1007142. doi: 10.1371/journal.pgen.1007142 (PMC5760082; doi:10.1371/journal.pgen.1007142)

| A  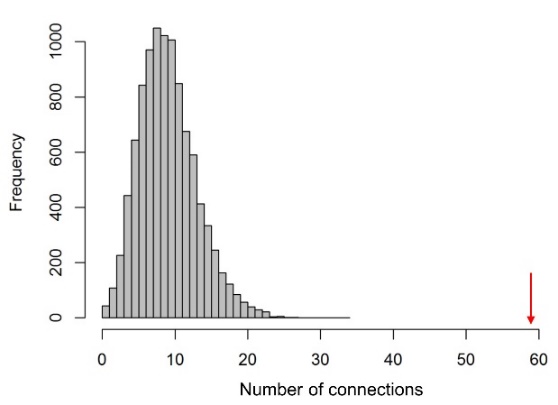 | B  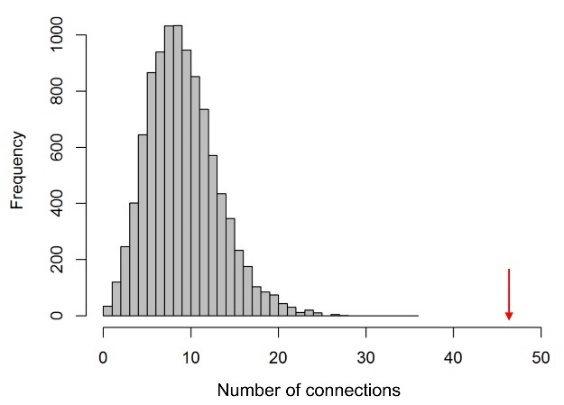 |
| --- | --- |
| C  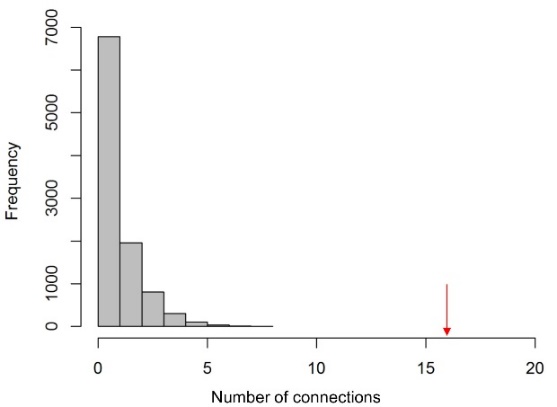 | D  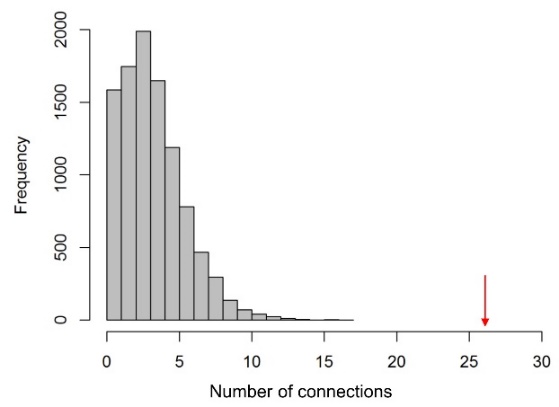 |
| E  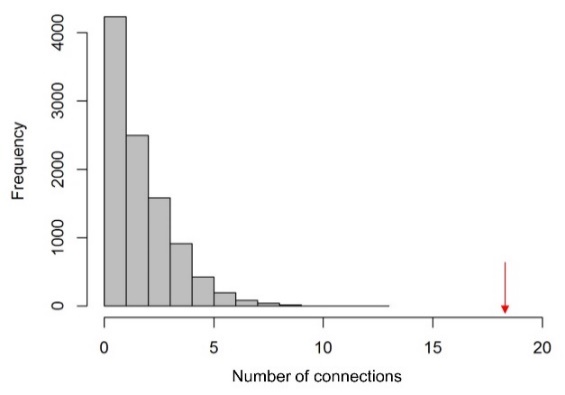 |  |

**S1 Fig. The network property of disease risk genes.**

Supplement: S1 Fig — Red arrows point to the number of connections among risk genes for the disease in the co-function network [17]. A null distribution is constructed by counting the number of connections among random genes (10000 iterations) with the same number of disease risk genes in the network. The risk genes of different diseases are listed in S1 Table. (A) Breast cancer. (B) Schizophrenia. (C) Tetralogy of Fallot. (D) Systemic lupus erythematosus. (E) Type 2 Diabetes. (DOCX) [file pgen.1007142.s001.docx]

| A  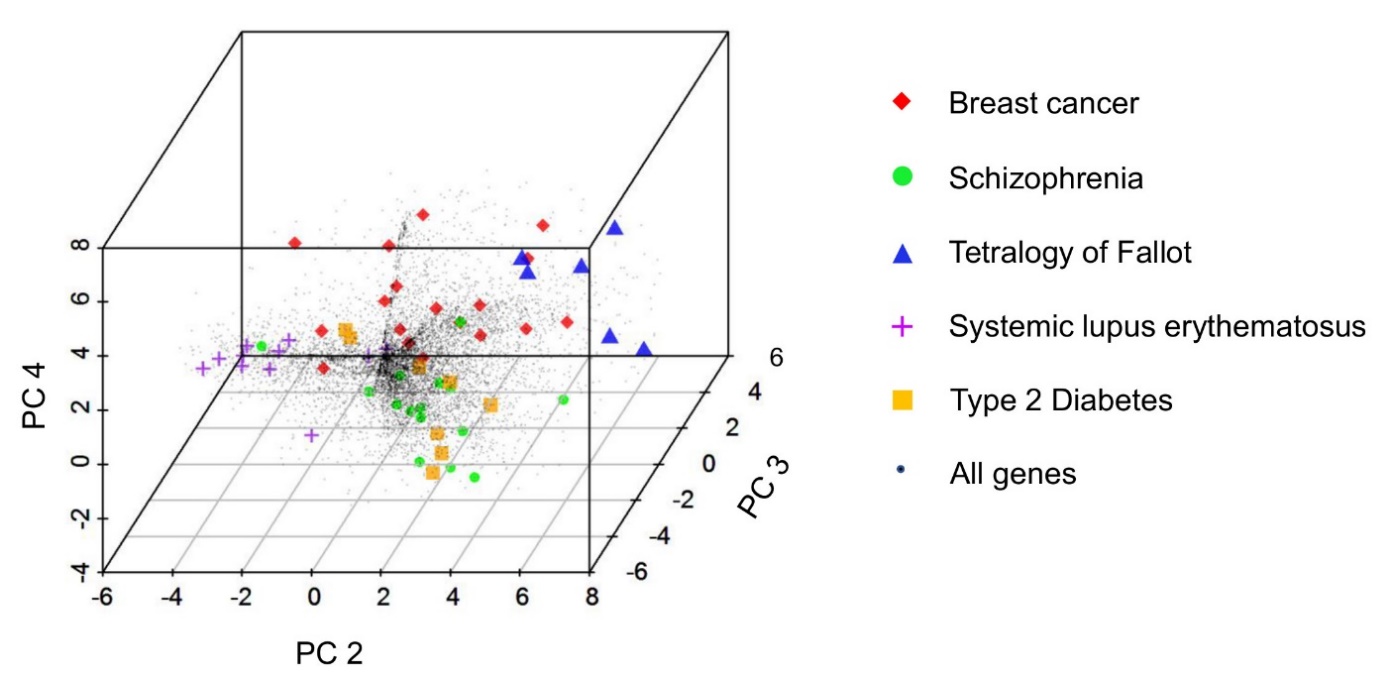 |
| --- |
| B  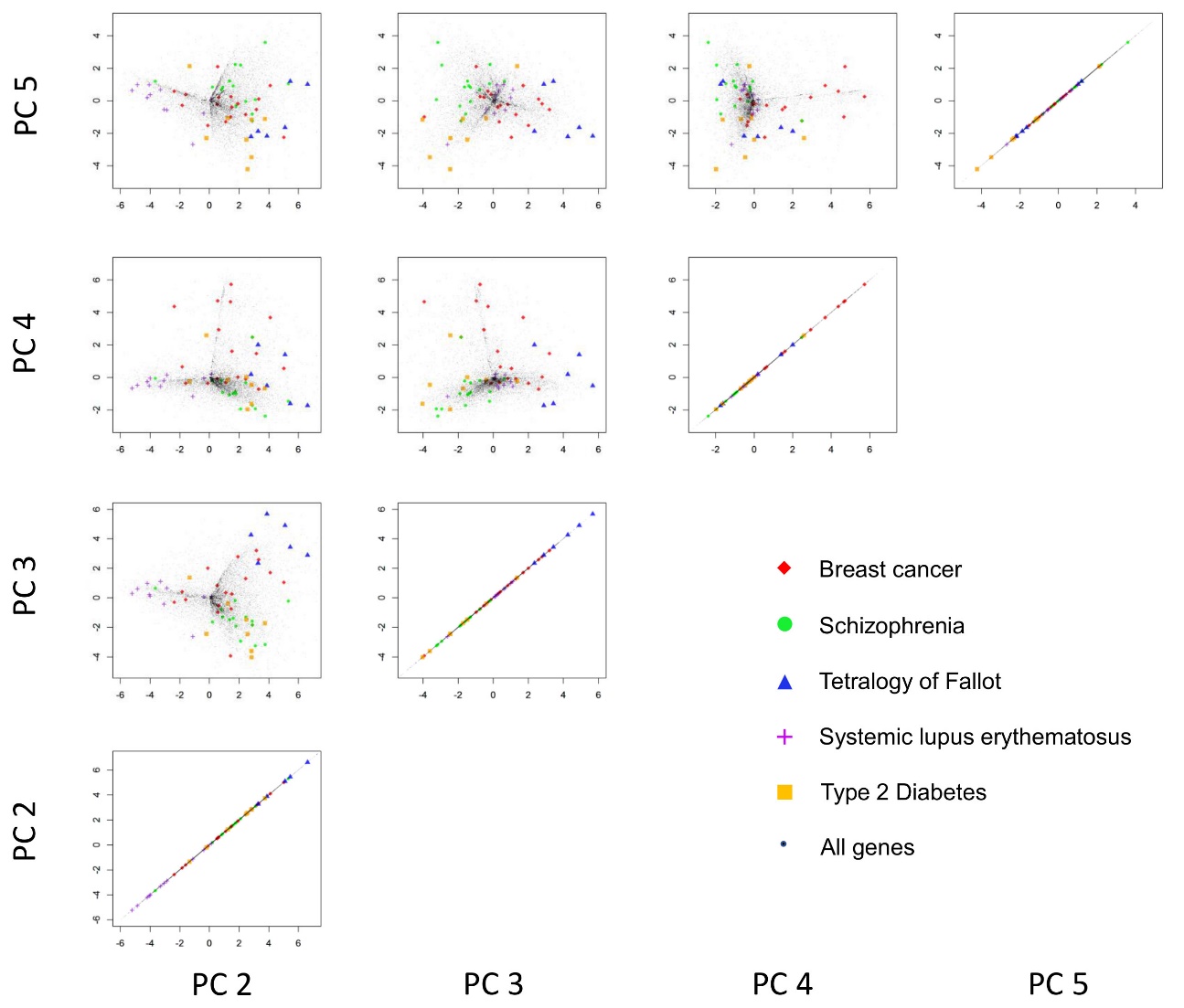 |
| C  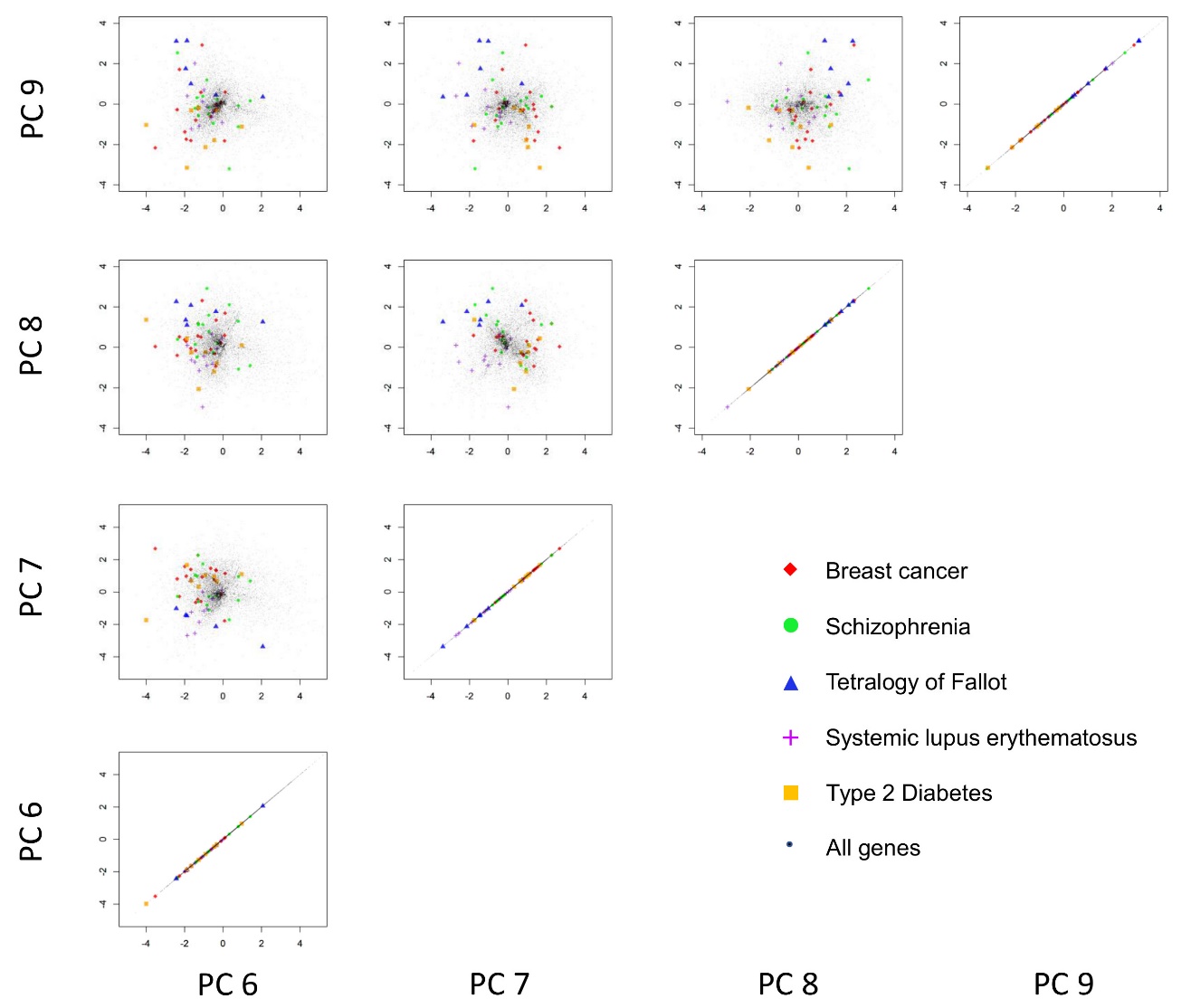 |

**S2 Fig. The phenotype property of disease risk genes.**

Supplement: S2 Fig — There is a tendency that disease risk genes tend to cluster on top principal components of MP phenotypes. The first principal component is excluded from consideration since it mainly characterizes the number of annotated MP terms (correlation coefficient = 0.918), which is not biological meaningful. The risk genes of different diseases are listed in S1 Table. (A) Disease risk genes on second, third, and forth principal components. (B) Disease risk genes on principal component 2 to 5. (C) Disease risk genes on principal component 6 to 9. (DOCX) [file pgen.1007142.s002.docx]

| A  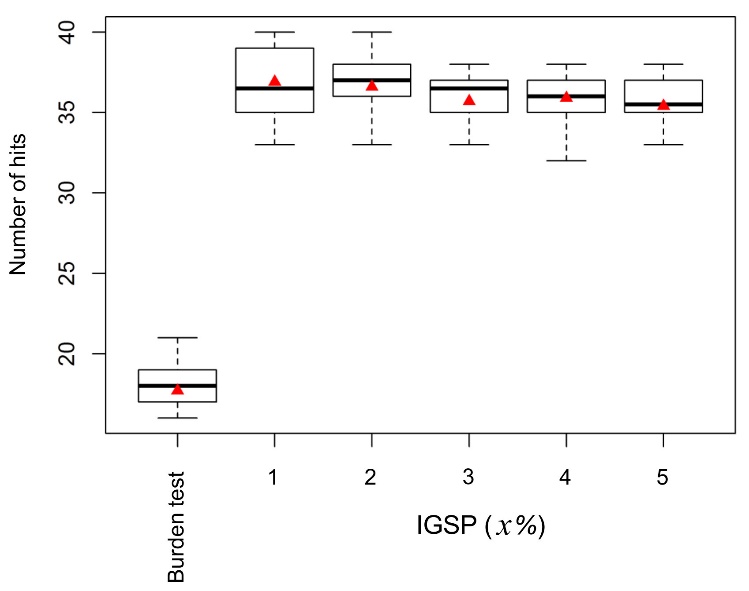 |
| --- |
| B  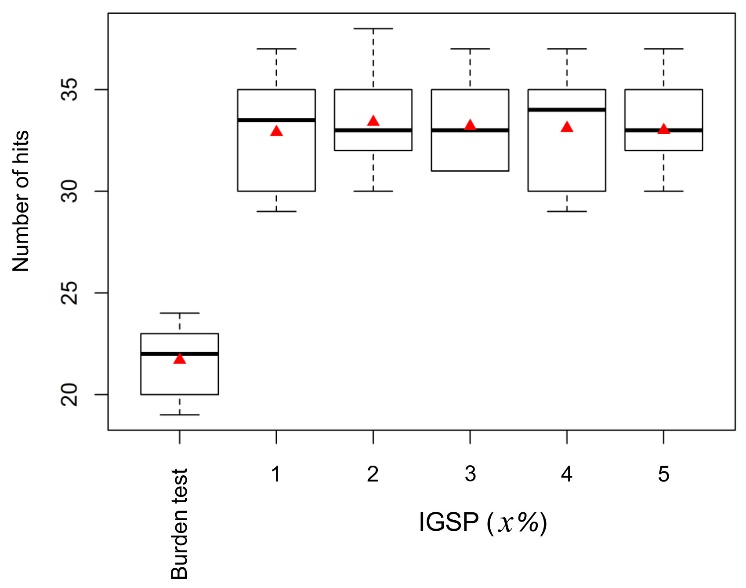 |

**S4 Fig. The performance of IGSP with different *x*.**

Supplement: S4 Fig — The number of risk genes in the top 50 high scoring genes (y axis) is used to evaluate the performance. The setup of the simulation: integrated scoring with both network and phenotype, “Moderate” association signal strength, a (0.1), b (1), principal components in phenotype scoring (PC 2 and 3). (A) 147 CHD genes (B) 193 SCZ genes. (DOCX) [file pgen.1007142.s004.docx]

| A  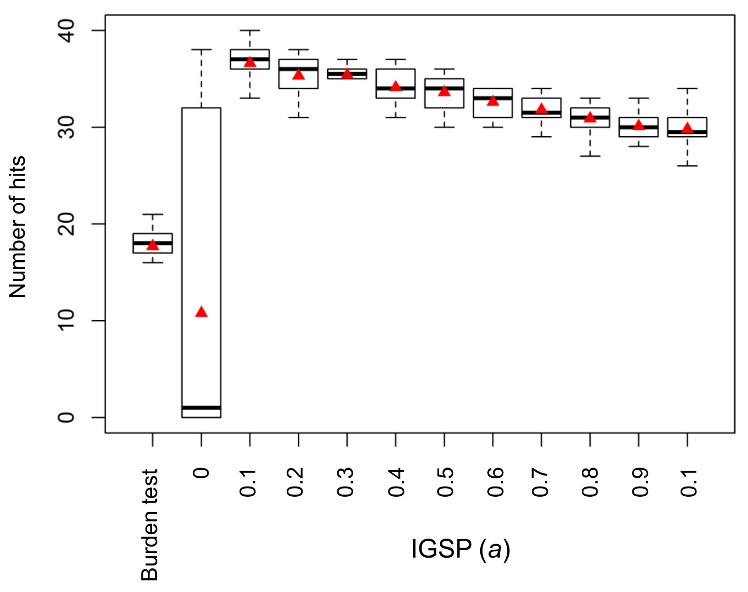 |
| --- |
| B  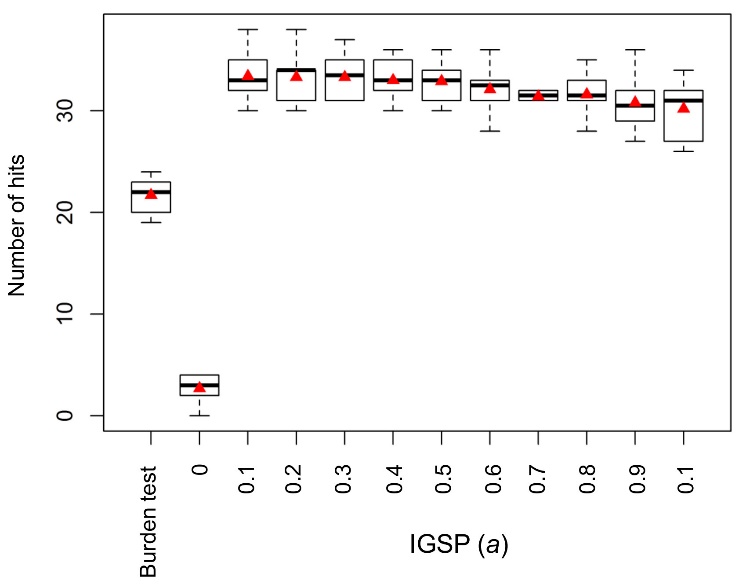 |

**S5 Fig. The performance of IGSP with different *a*.**

Supplement: S5 Fig — The number of risk genes in the top 50 high scoring genes (y axis) is used to evaluate the performance. The setup of the simulation: integrated scoring with both network and phenotype, “Moderate” association signal strength, x (2), b (1), and principal components in phenotype scoring (PC 2 and 3). (A) 147 CHD genes (B) 193 SCZ genes. (DOCX) [file pgen.1007142.s005.docx]

| A  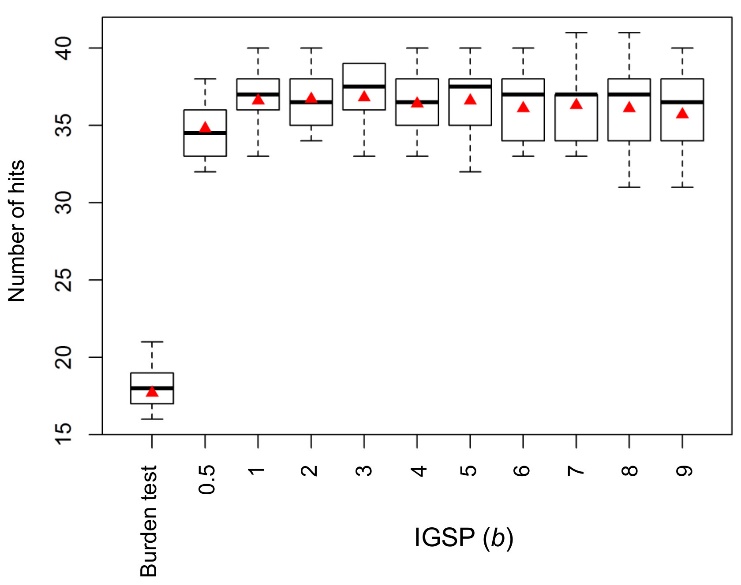 |
| --- |
| B  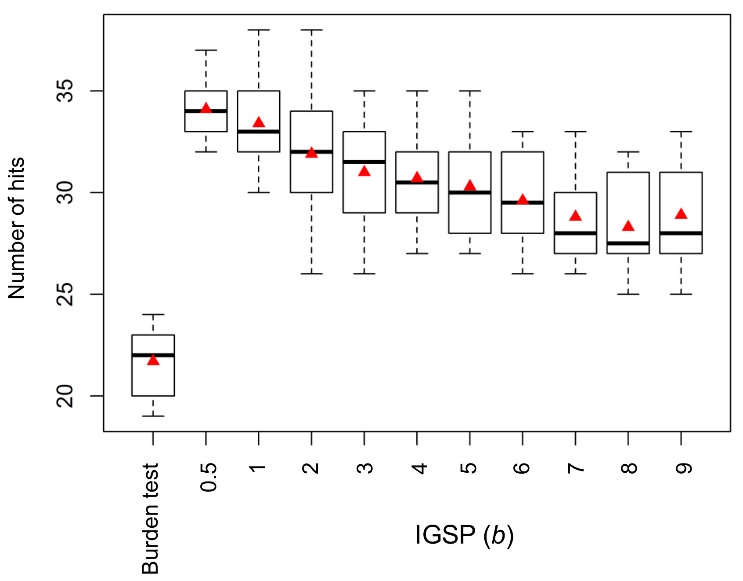 |

**S6 Fig. The performance of IGSP with different *b*.**

Supplement: S6 Fig — The number of risk genes in the top 50 high scoring genes (y axis) is used to evaluate the performance. The setup of the simulation: integrated scoring with both network and phenotype, “Moderate” association signal strength, x (2), a (0.1), and principal components in phenotype scoring (PC 2 and 3). (A) 147 CHD genes (B) 193 SCZ genes. (DOCX) [file pgen.1007142.s006.docx]

| A  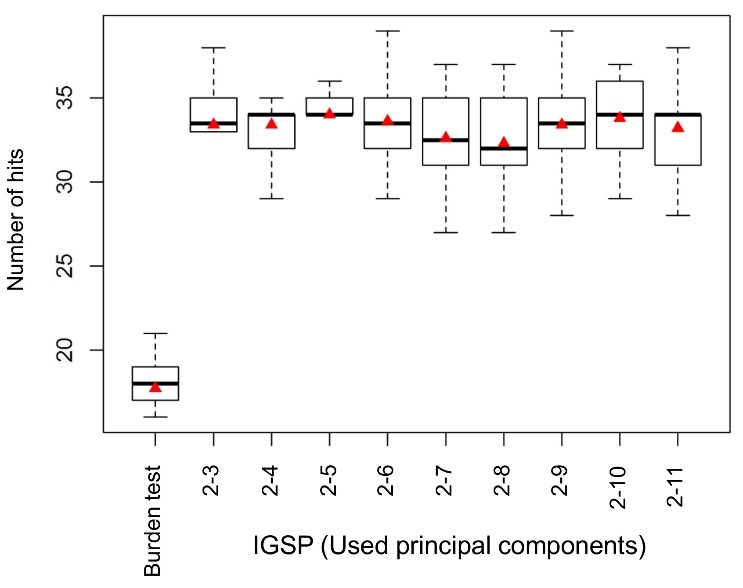 |
| --- |
| B  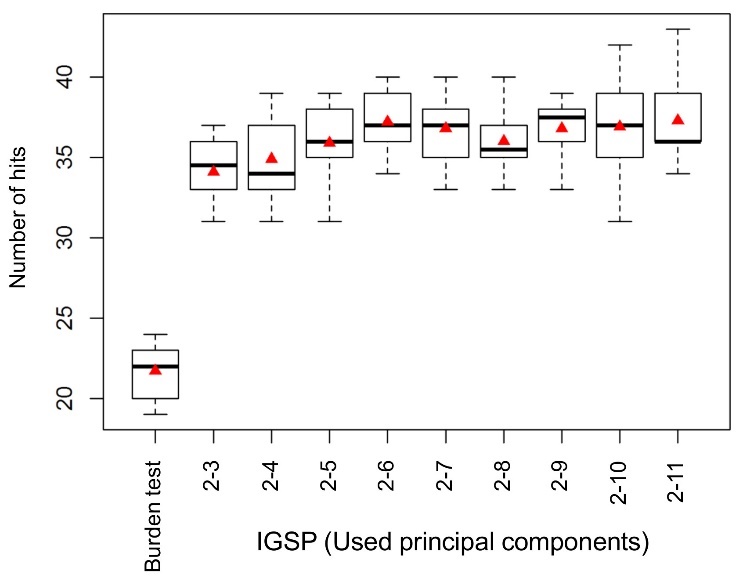 |

**S7 Fig. The performance of IGSP with different top principal components in phenotype scoring.**

Supplement: S7 Fig — The number of risk genes in the top 50 high scoring genes (y axis) is used to evaluate the performance. The setup of the simulation: integrated scoring with phenotype information, “Moderate” association signal strength, x (2), a (0.1), and b (1). (A) 147 CHD genes (B) 193 SCZ genes. (DOCX) [file pgen.1007142.s007.docx]

| A  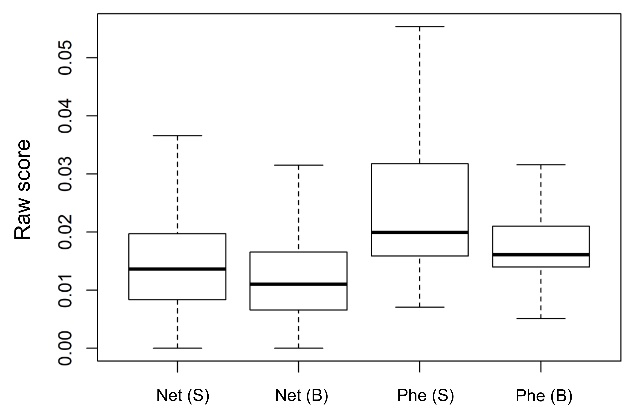 |
| --- |
| B  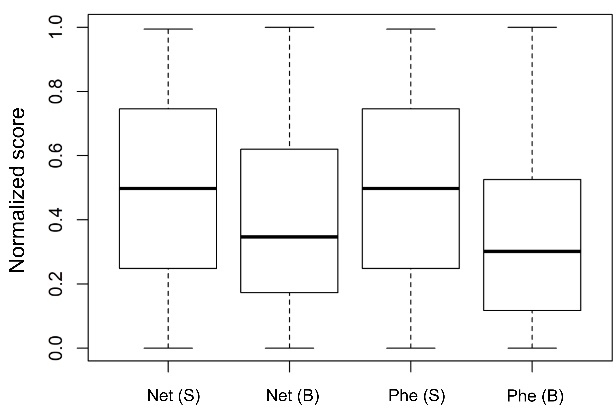 |

**S8 Fig. The distribution of network and phenotype scores.**

Supplement: S8 Fig — The distribution of scores was constructed based on the network or phenotype scores of IGSP-scoring genes at the 3000th iteration of a trial in CHD simulation. 'S' denotes sampled risk genes while 'B' denotes the other background genes at that iteration. The setup of the simulation: integrated scoring with both network and phenotype, 'Moderate' association signal strength, x = 2, a = 0.1, b = 1, and principal components in phenotype scoring (PC 2 and 3). (A) The raw network and phenotype scores. (B) The network and phenotype scores after normalization. (DOCX) [file pgen.1007142.s008.docx]

| A  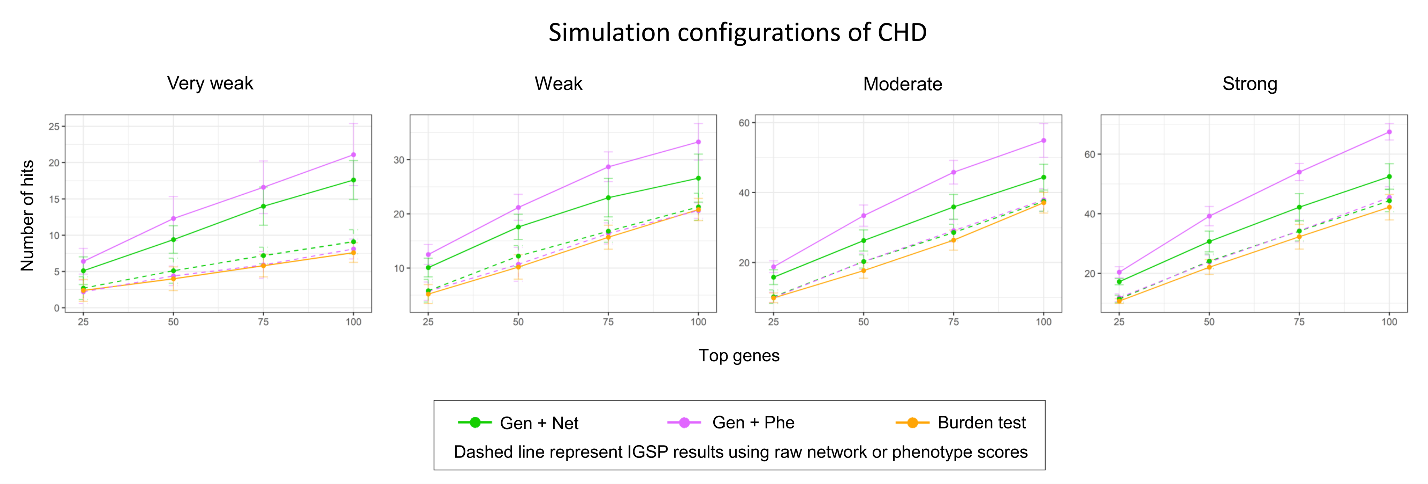 |
| --- |
| B  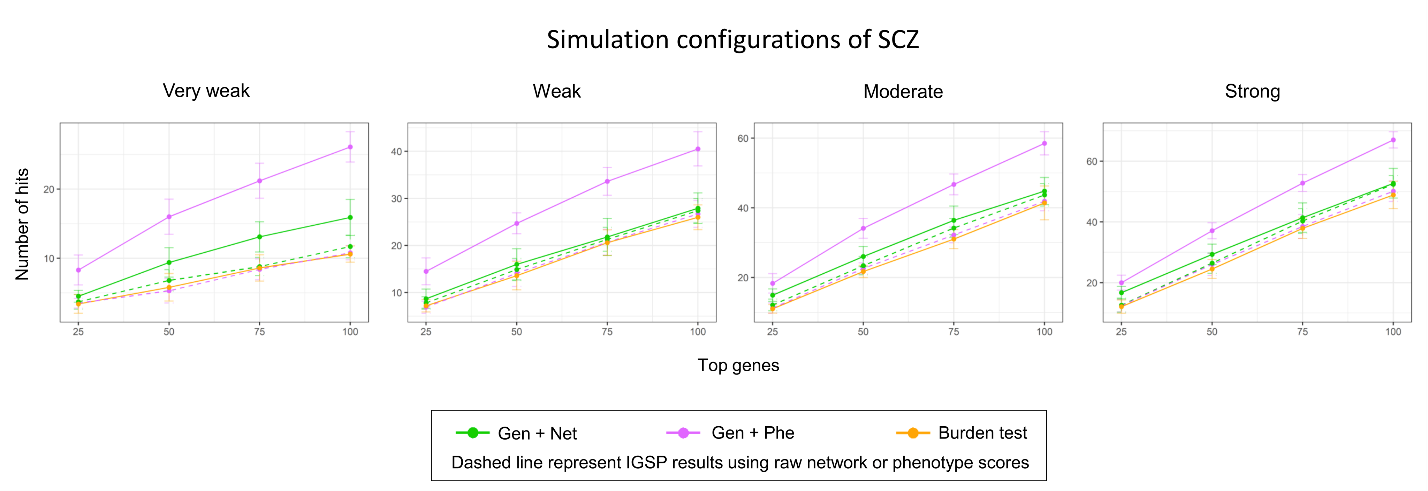 |

**S9 Fig. Normalization of network and phenotype raw scores is a necessary step for IGSP.**

Supplement: S9 Fig — The result showed that IGSP using raw network and phenotype scores without normalization barely improved upon gene association signals. 'Gen + Net' and 'Gen + Phe' represent IGSP with only integration of network features and phenotype features, respectively. The parameters used in this simulation were as follows: x = 2, a = 0.1, b = 1, and principal components in phenotype scoring (PC 2 and 3). (A) CHD. 147 CHD genes from Sifrim et al [26] were used as the risk genes. (B) Schizophrenia. 193 putative schizophrenia genes from MalaCards [57] were used as the risk genes. (DOCX) [file pgen.1007142.s009.docx]

| A  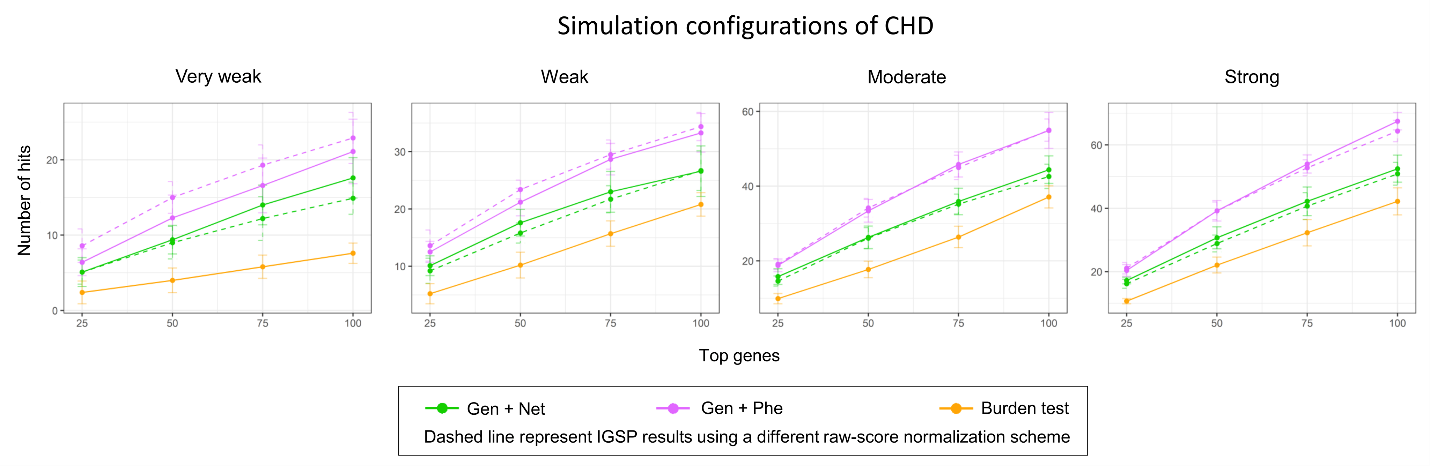 |
| --- |
| B  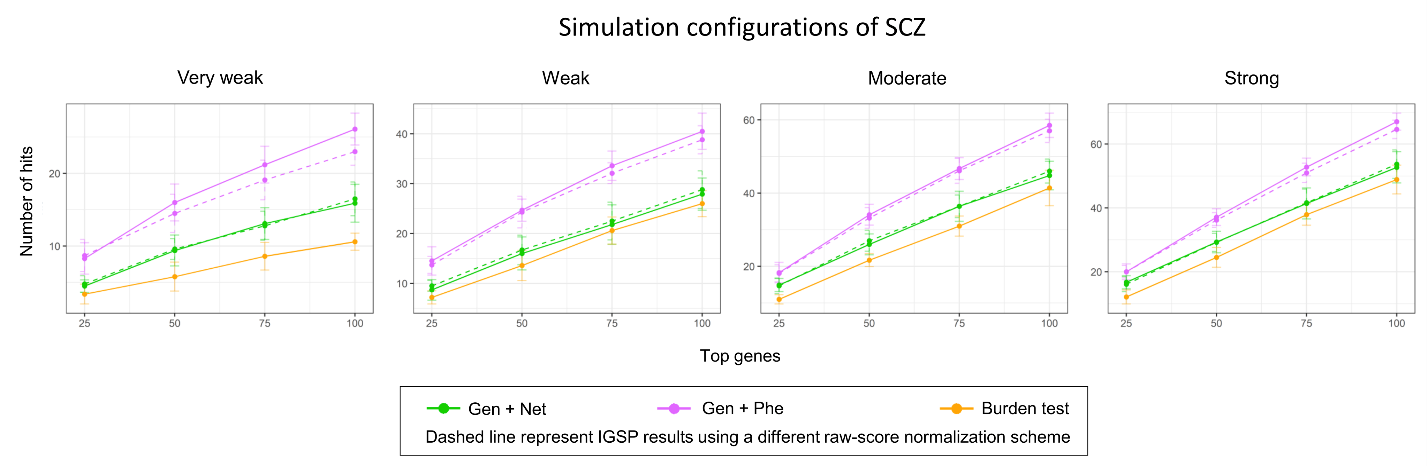 |

**S10 Fig. IGSP works with different normalization methods for raw score normalization.**

Supplement: S10 Fig — The dashed line represents IGSP results using a different raw-score normalization method: (score–min (scores)) / max(scores)–min(scores)). 'Gen + Net' and 'Gen + Phe' represent IGSP with only integration of network features and phenotype features, respectively. The parameters used in this simulation were as follows: x = 2, a = 0.1, b = 1, and principal components in phenotype scoring (PC 2 and 3). (A) CHD. 147 CHD genes from Sifrim et al [26] were used as the risk genes. (B) Schizophrenia. 193 putative schizophrenia genes from MalaCards [57] were used as the risk genes. (DOCX) [file pgen.1007142.s010.docx]

| A  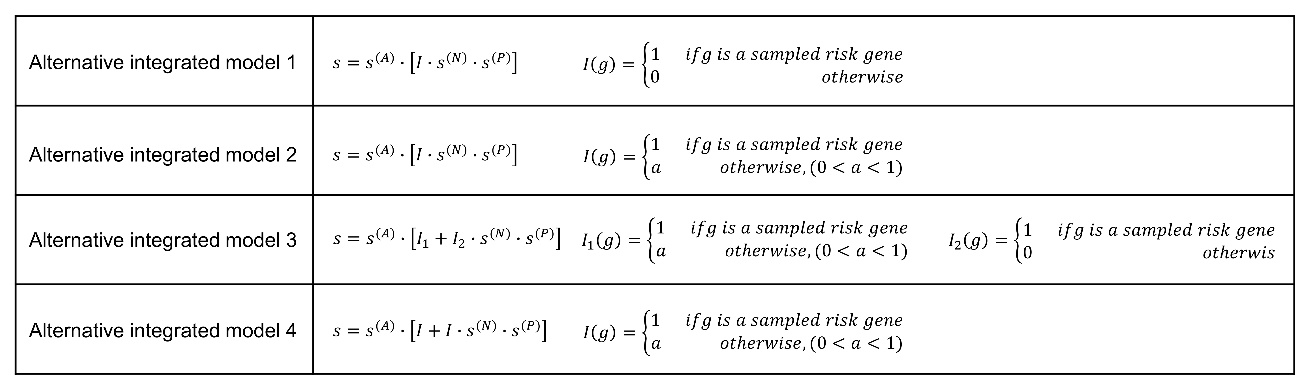 |
| --- |
| B  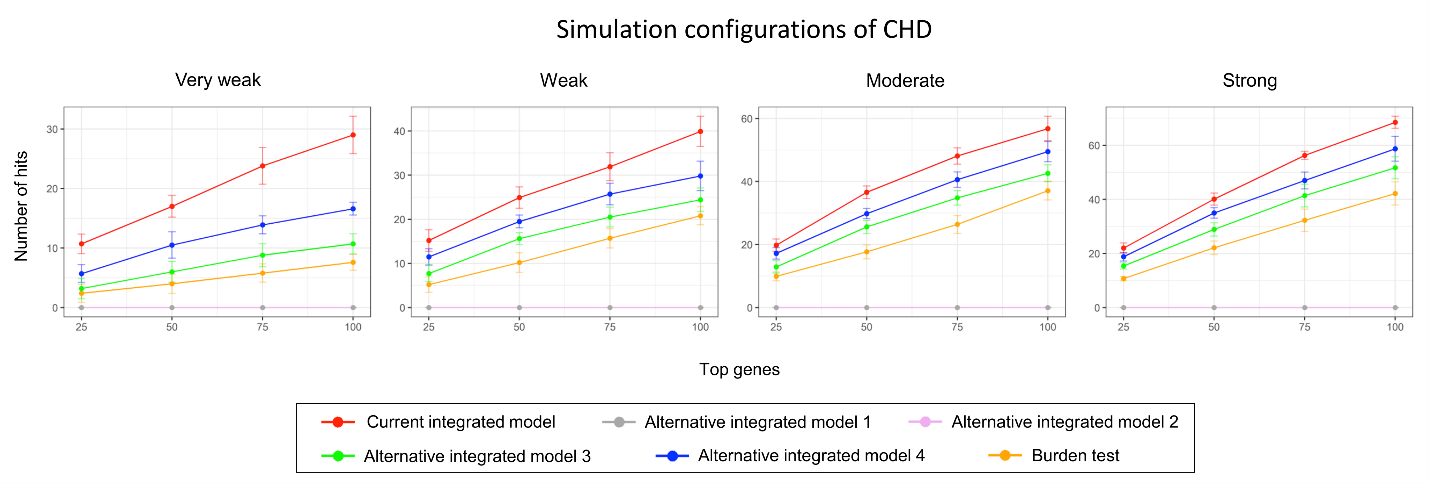 |
| C  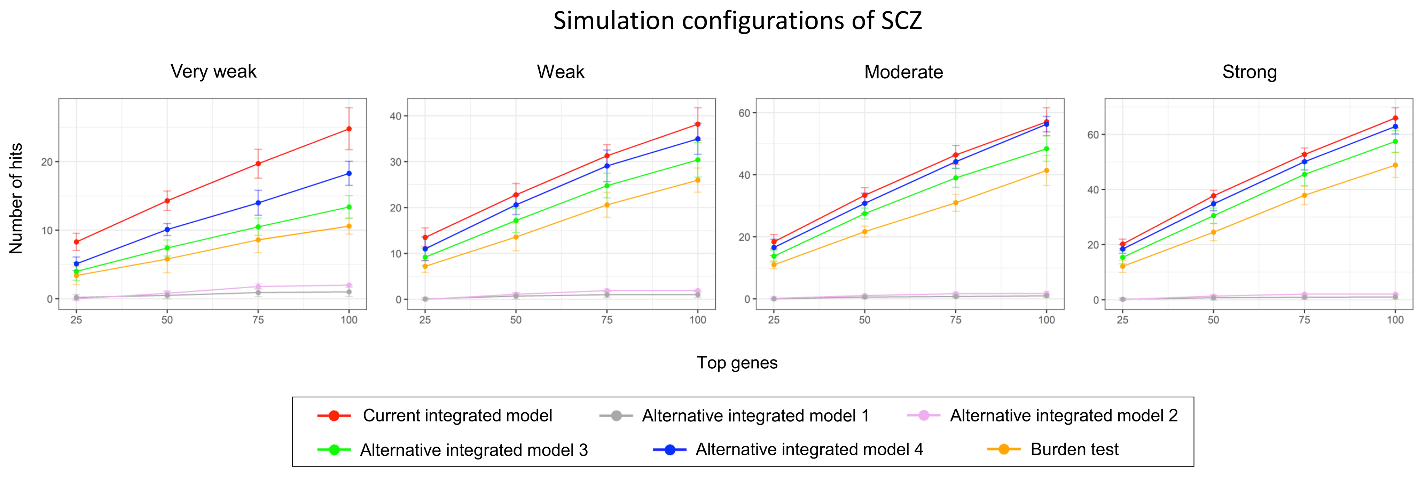 |

**S12 Fig. The performance evaluation of using different integrated models.**

Supplement: S12 Fig — The performance of using our current integrated model (Eq 1) was compared with that of using four other different integrated models; full integration of network and phenotype features was applied in performance evaluation. The parameters used in this simulation were as follows: x = 2, a = 0.1, and principal components in phenotype scoring (PC 2 and 3). b was set as 1 for the current integrated model but as an indicator variable defined below. (A) Definition of four alternative integrated models. In these models, the weight of network and phenotype components, b, is set as an indicator variable dependent on risk gene status. (B) CHD. 147 CHD genes from Sifrim et al [26] were used as the risk genes. (C) Schizophrenia. 193 putative schizophrenia genes from MalaCards [57] were used as the risk genes. (DOCX) [file pgen.1007142.s012.docx]

| A  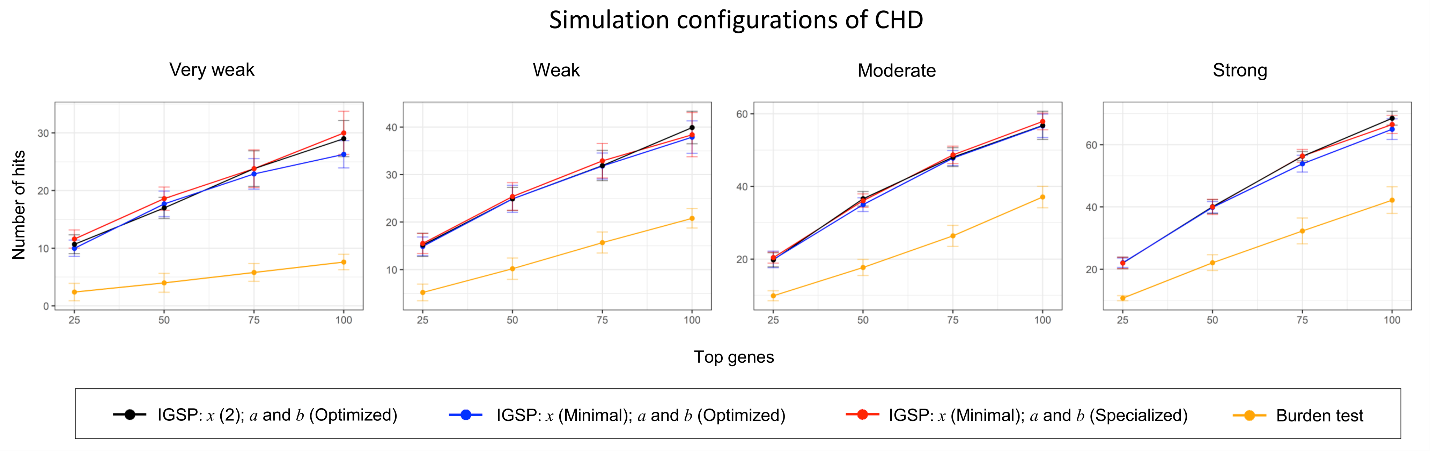 |
| --- |
| B  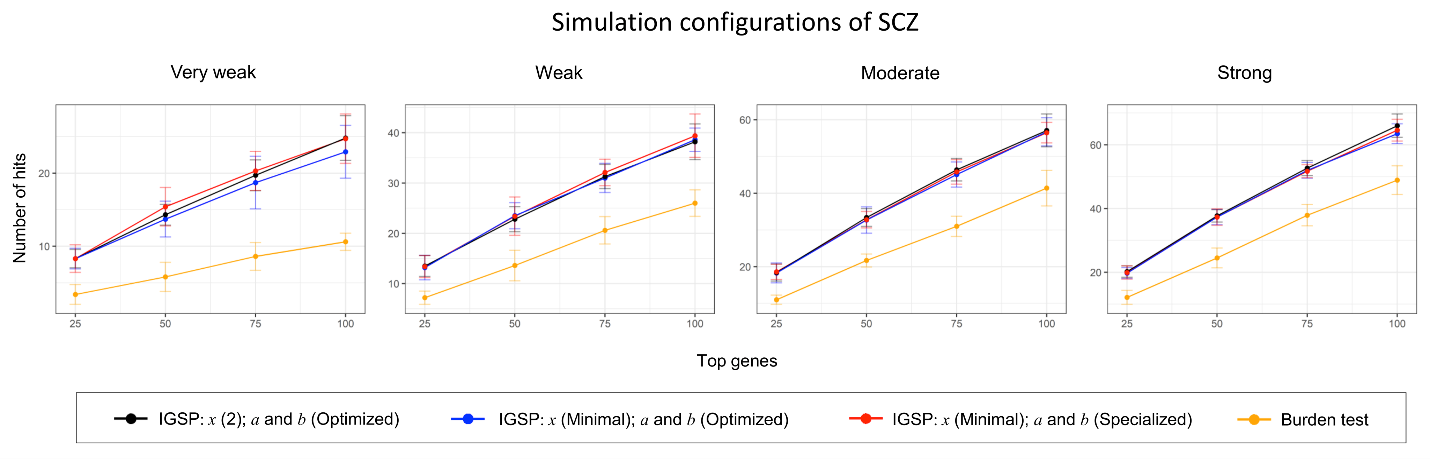 |

**S14 Fig. The performance of IGSP with 30 sampling risk genes and a specialized scaling range.**

Supplement: S14 Fig — The performance of IGSP using a minimal number of sampling risk genes and a specialized scaling range is compared with that of IGSP using the optimized setting of x, a, and b. Full integration of network and phenotype features was applied in performance evaluation. The parameters used in this simulation were shown in figures and as follows: principal components in phenotype scoring (PC 2 and 3). 'Minimal' represents x approximating 30 sampling risk genes; 'Optimized' represents (a, b) = (0.1, 1); 'Specialized' represents a setting of a and b in which a is set as 0.9999999 (≈ 1) and b is set as (the highest association score in the sequencing dataset /–log10(0.5))– 1. (A) CHD. 147 CHD genes from Sifrim et al [26] were used as the risk genes. (B) Schizophrenia. 193 putative schizophrenia genes from MalaCards [57] were used as the risk genes. (DOCX) [file pgen.1007142.s014.docx]

| 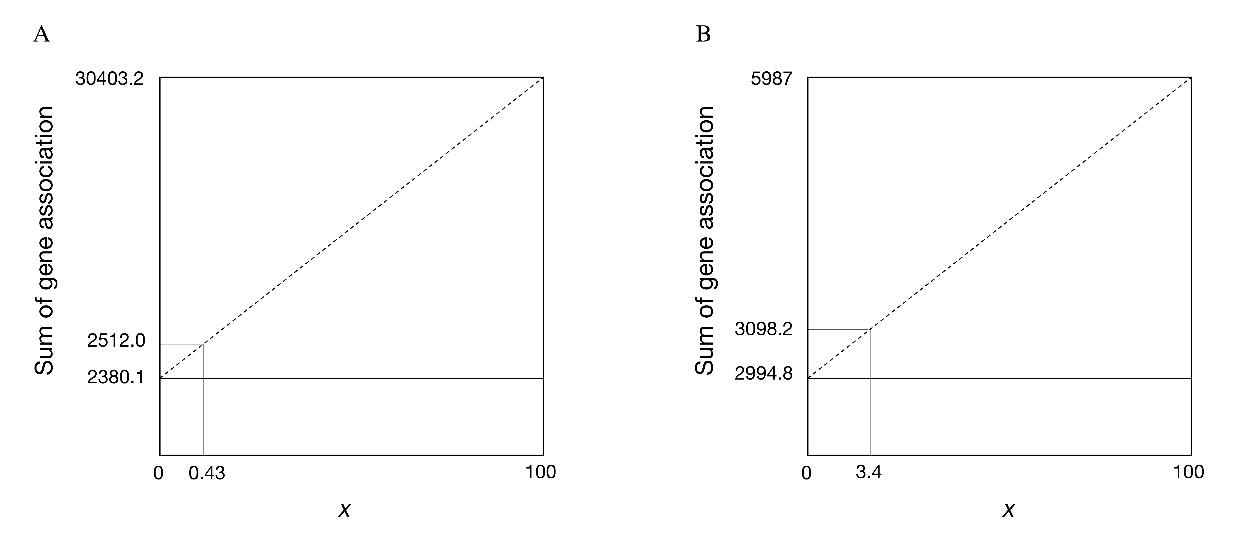 |  |
| --- | --- |

**S15 Fig. Prediction of *x* from the CHD sequencing data.**

Supplement: S15 Fig — Genes with stronger association signals (i.e., with smaller P-values) are more likely to be risk genes. This method predicts x based on the difference between the strength of disease association and null association against the corresponding difference when all genes have robust disease association and thus are risk genes. Association signals of CHD sequencing data were obtained from the burden test used in our method. Null association signals were obtained from the burden test used in our method by permuting the original disease status label. We predicted a range of x based on two different metrics in measuring gene association strength. (A)–log10P. For 5,987 scoring genes, the sum of disease association and the average sum of null association for 1,000 sets of null association signals were 2512.0 and 2380.1, respectively. The (minimum) sum of robust disease association was calculated based on a scenario when each scoring gene has a P-value surviving multiple correction: (–log10(0.05/5987)×5987) = 30403.2. We predicted x = 0.43 percent of scoring genes associated with the disease in this sequencing data based on a linear interpolation. Compared with the other metric in measuring association strength, this metric can better estimate the number of risk genes with strong association but may underestimate the number of risk genes with weak association. (B) 1–P. For 5,987 scoring genes, the sum of disease association and the average sum of null association for 1,000 sets of null association signals were 3098.2 and 2994.8, respectively. The sum of robust disease association was calculated based on a scenario when each scoring gene has a P-value surviving multiple correction: (1–0.05/5987) × 5987 ≈ 5987. We predicted x = 3.4 percent of scoring genes associated with the disease in this sequencing data based on a linear interpolation. (DOCX) [file pgen.1007142.s015.docx]

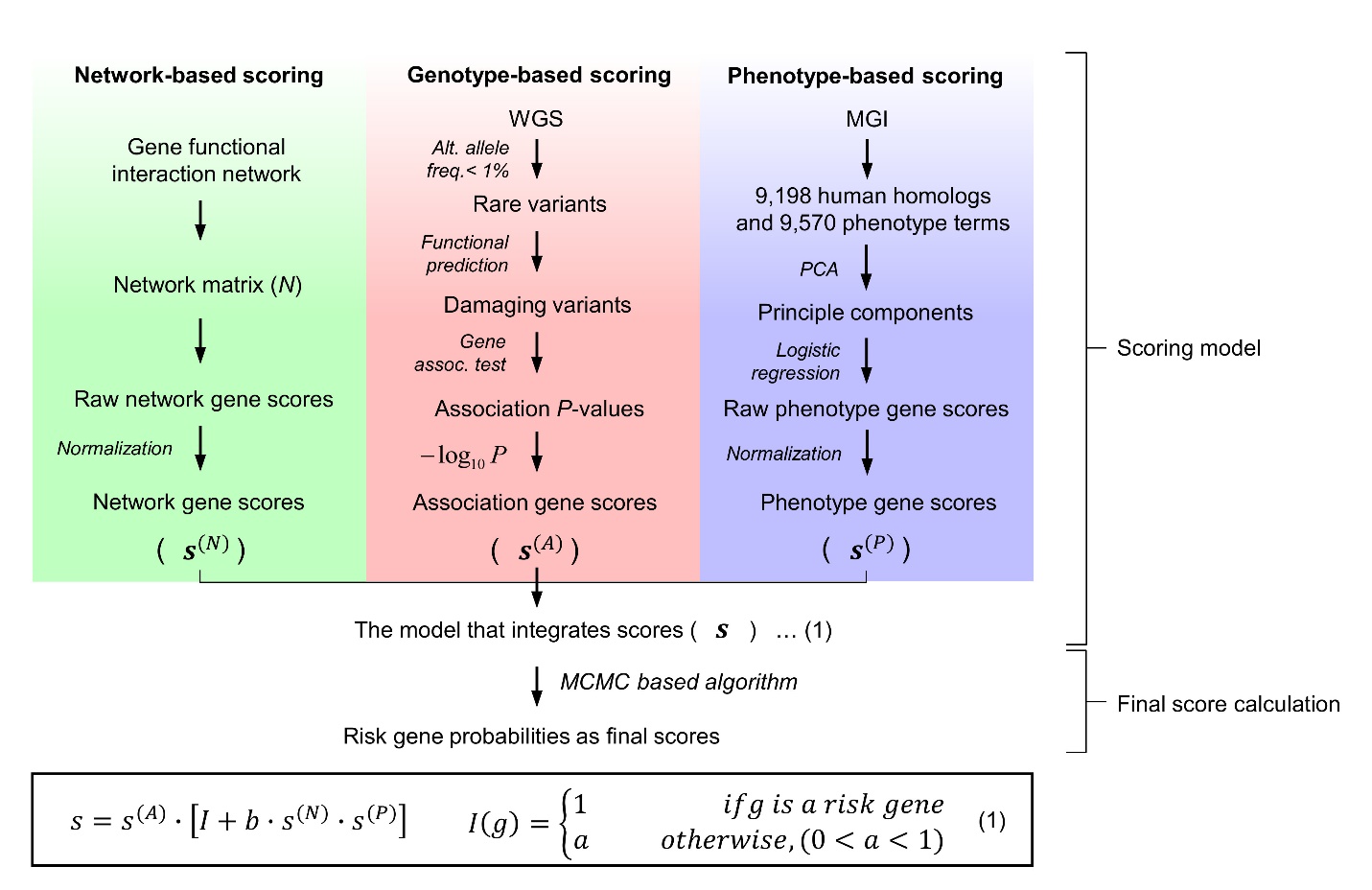


**Figure S16. The workflow of IGSP.**

Supplement: S16 Fig — The workflow of IGSP can be divided into two parts: The scoring model of risk genes and calculation of final gene scores. Given association signals s(A), the scoring model will determine a multivariate probability distribution of integrated scores (s) (bold denotes vectors). In final score calculation, we applied Markov chain Monte Carlo (MCMC) in our algorithm to approximate risk gene probabilities as final gene scores according to the multivariate probability distribution of integrated scores. (DOCX) [file pgen.1007142.s016.docx]

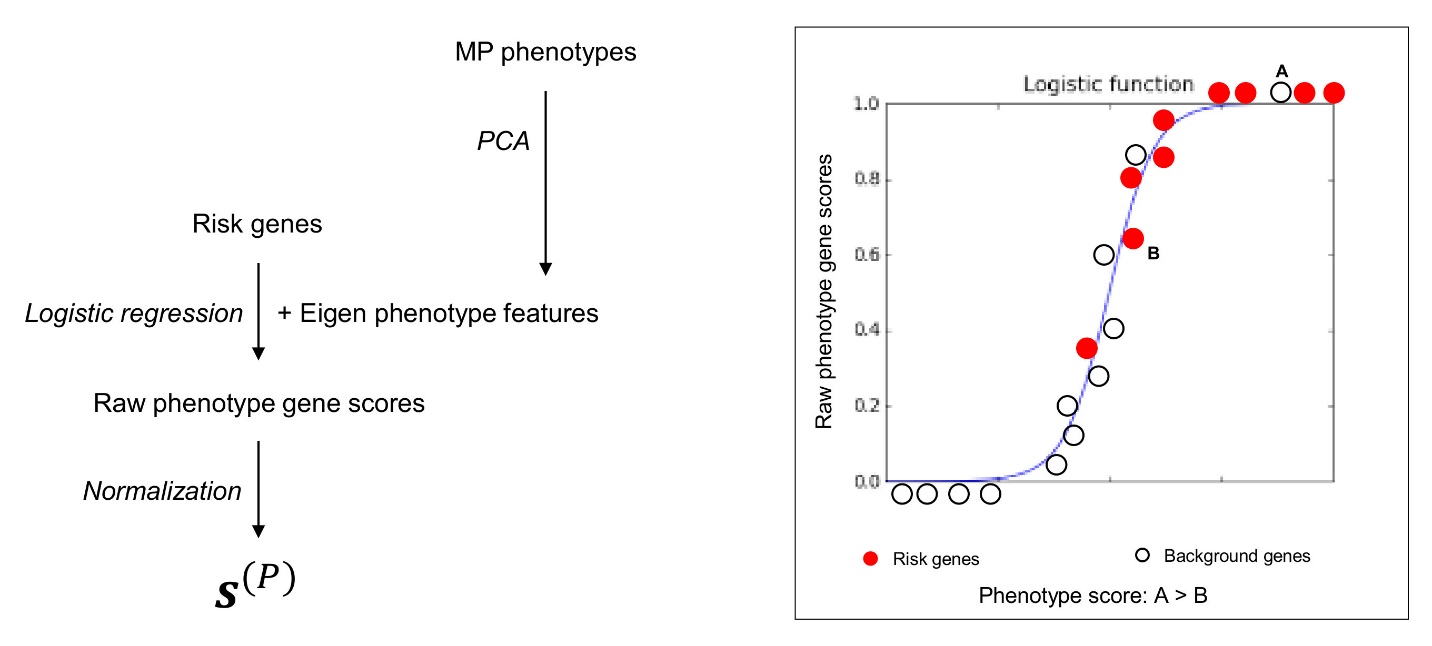


**S17 Fig. Phenotype-based scoring.**

Supplement: S17 Fig — Principal components from 9,198 human homologs and 9,570 MP terms are used to characterize the phenotype features of each gene. Genes with a higher propensity to share phenotype similarities with risk genes have higher phenotype scores. The combination of risk genes is the same latent variable shown in network-based scoring (S18 Fig). (DOCX) [file pgen.1007142.s017.docx]

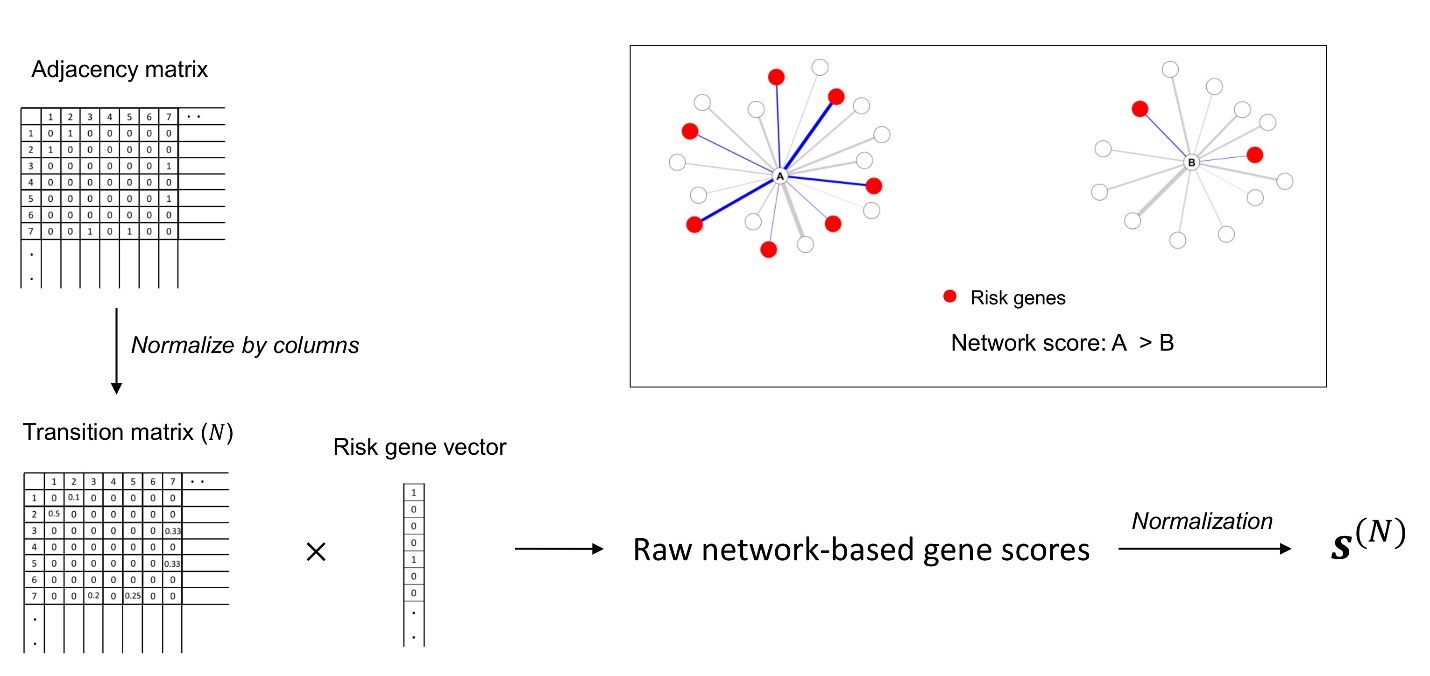


**S18 Fig. Network-based scoring.**

Supplement: S18 Fig — Genes with a higher propensity to be connected to risk genes have higher network scores. A and B can be risk genes themselves and their network scores are calculated in the same manner. The risk gene vector depends on the combination of risk genes (d), which is a latent variable in our model. (DOCX) [file pgen.1007142.s018.docx]

| A  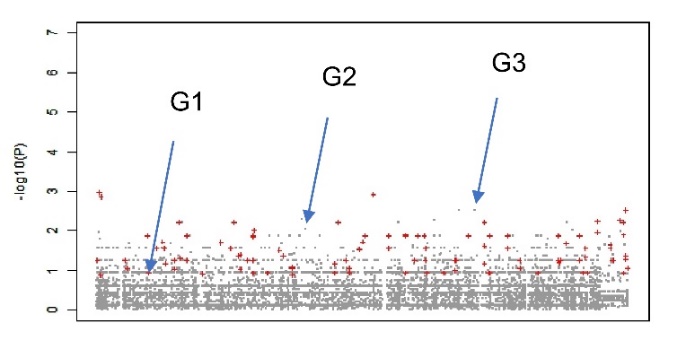 |
| --- |
| B  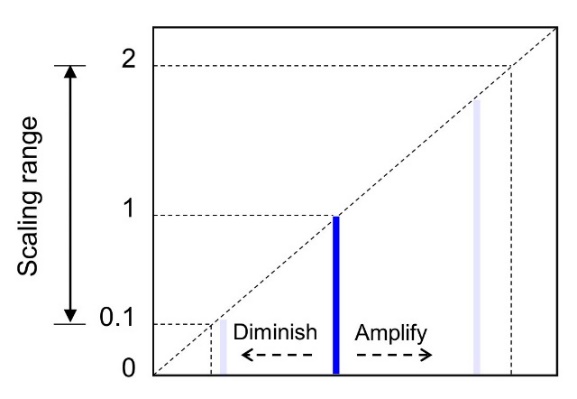 |
| C  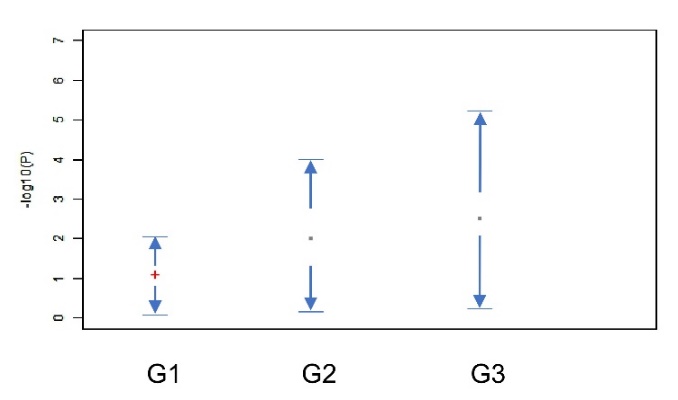 |

**S19 Fig. Integrated scores as adjusted signals in IGSP.**

Supplement: S19 Fig — G1 is a risk gene, while G2 and G3 are two non-risk genes that have a stronger association signal than G1. (A) Gene association signals. Gene association signals are obtained through gene association tests on the investigated sequencing data. In the figure, a red cross represents a risk gene, while a grey dot represents a non-risk gene. Association signals of risk genes are buried in noise. (B) Signal adjustment by linear scaling. IGSP integrates association signals with network and phenotype evidence by multiplying a scaling coefficient to original gene association signals. The scaling coefficient of each gene incorporates its network and phenotype information, as shown in Eq 1. Every gene has the same scaling range. (C) The adjustment ranges of different genes. While the scaling ranges of all genes are the same, the adjustment range of each gene depends on the magnitude of its original signal. The final score of each gene is calculated as its risk gene probability based on the multivariate probability distribution of adjusted signals within the adjustment ranges determined by Eq 1. (DOCX) [file pgen.1007142.s019.docx]

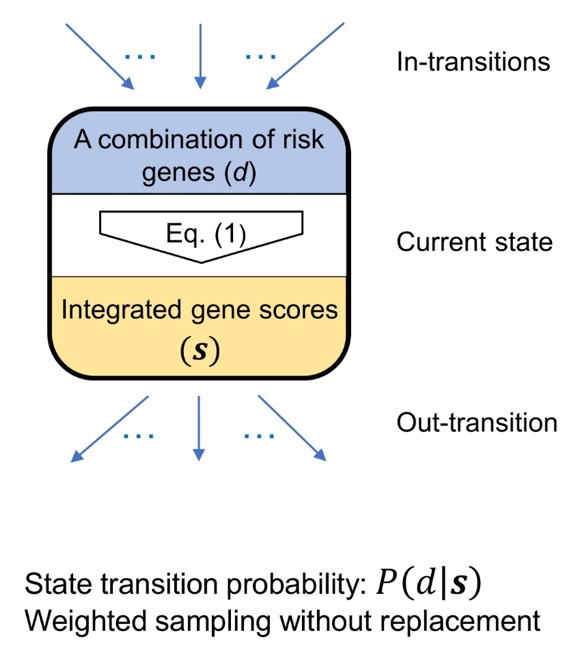


**S20 Fig. The underlying Markov chain of IGSP.**

Supplement: S20 Fig — Each combination of risk gene determines a state. The number of states in this Markov chain is the number of possible combination of risk genes. Given association signals, a combination of risk genes determines integrated gene scores according to Eq 1; the transition probability determining progression to the next state is equal to the weighted sampling n from m genes without replacement, using s as weight. Instead of calculating the transition probability, our MCMC-based algorithm carries out state transition using the MATLAB “datasample” function with integrated scores s as weight. The Markov chain is ergodic (irreducible and aperiodic) and hence has only a single equilibrium distribution. When equilibrium is reached, the probability of visiting a state (d) approximates ∑siP(d|si)×P(si|s(A)) in Eq 3. (DOCX) [file pgen.1007142.s020.docx]

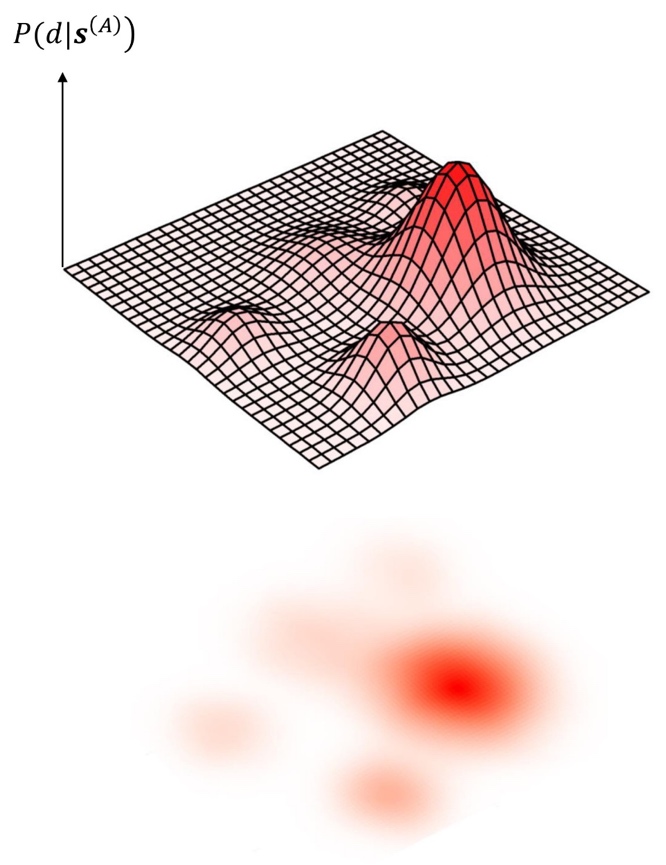


**S21 Fig. The probability landscape and MCMC sampling of IGSP.**

Supplement: S21 Fig — The upper plot shows the probability landscape of different combinations of risk genes, d, given association gene signals s(A). In the upper plot, each point on the plan represents a combination of risk genes. The redness in the bottom plot represents the frequency of visiting the corresponding states (see S20 Fig). (DOCX) [file pgen.1007142.s021.docx]
