# Supplementary material for: Integrated rare variant-based risk gene prioritization in disease case-control sequencing studies
Source: PLoS Genet. 2017 Dec 27;13(12):e1007142. doi: 10.1371/journal.pgen.1007142 (PMC5760082; doi:10.1371/journal.pgen.1007142)
Supplement: S3 Fig — Using the CHD case-control WES data in our case study, we collected 1,000 sets of null gene association signals. Each null set was obtained from the burden test used in our method by permuting the original disease status label. We then tested whether there’s a relationship between genotype-based scoring and the co-function network degree or the number of associated MP terms. The relationship between genotype-based scoring and the co-function network degree was evaluated based on genes with both association scores and network degrees. The relationship between genotype-based scoring and the number of associated MP terms was evaluated based on genes with both association scores and phenotype scores (i.e. genes with at least one associated MP terms). (A) Correlation coefficient. The median correlation coefficients were 0.0067 and 0.0082 between the association score and the co-function network degree ('G and N') and between the association score and the number of associated MP terms ('G and P'), respectively. (B) Correlation P-value. The median P-values were 0.469 and 0.523 for the correlation between the association score and the co-function network degree and the correlation between the association score and the number of associated MP terms, respectively. (DOCX) [file pgen.1007142.s003.docx]

| A  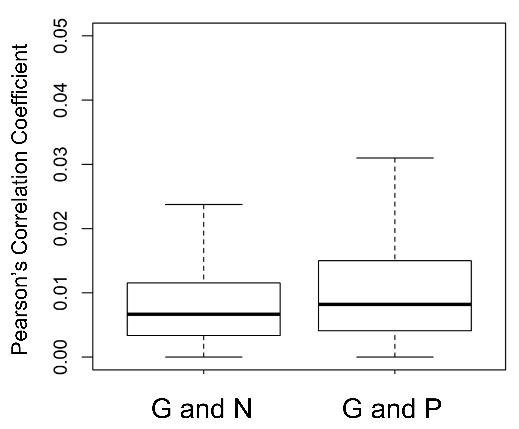 | B  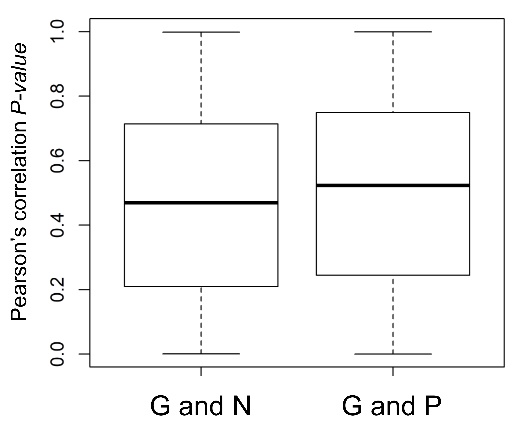 |
| --- | --- |

**S3 Fig.** **Genotype-based scoring** **has no relationship with co-function network degree and the number of associated MP terms.**
