# Supplementary material for: Integrated rare variant-based risk gene prioritization in disease case-control sequencing studies
Source: PLoS Genet. 2017 Dec 27;13(12):e1007142. doi: 10.1371/journal.pgen.1007142 (PMC5760082; doi:10.1371/journal.pgen.1007142)
Supplement: S11 Fig — 'Gen + Net' represents IGSP with only integration of network features. We compared the performance of using two different types of network connectivity (between a scoring gene and possible risk genes) in network-based scoring. C1 represents the network connectivity based on a transition matrix (considering network degree). C2 represents the network connectivity based on an adjacency matrix (without considering network degree). The parameters used in this simulation were as follows: x = 2, a = 0.1, b = 1, and principal components in phenotype scoring (PC 2 and 3). (A) CHD. 147 CHD genes from Sifrim et al [26] were used as the risk genes. (B) Schizophrenia. 193 putative schizophrenia genes from MalaCards [57] were used as the risk genes. (DOCX) [file pgen.1007142.s011.docx]

| A  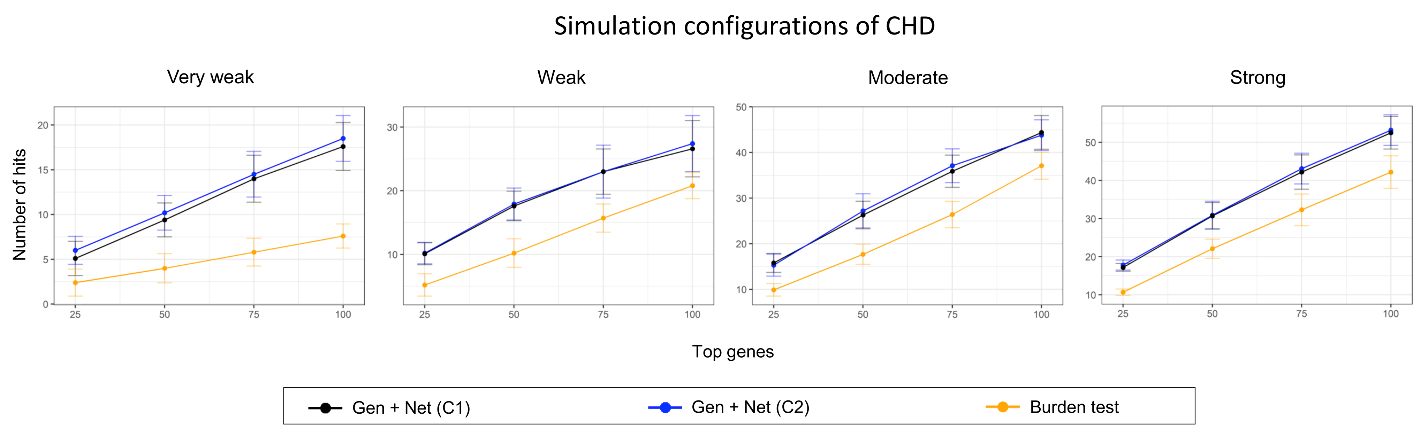 |
| --- |
| B  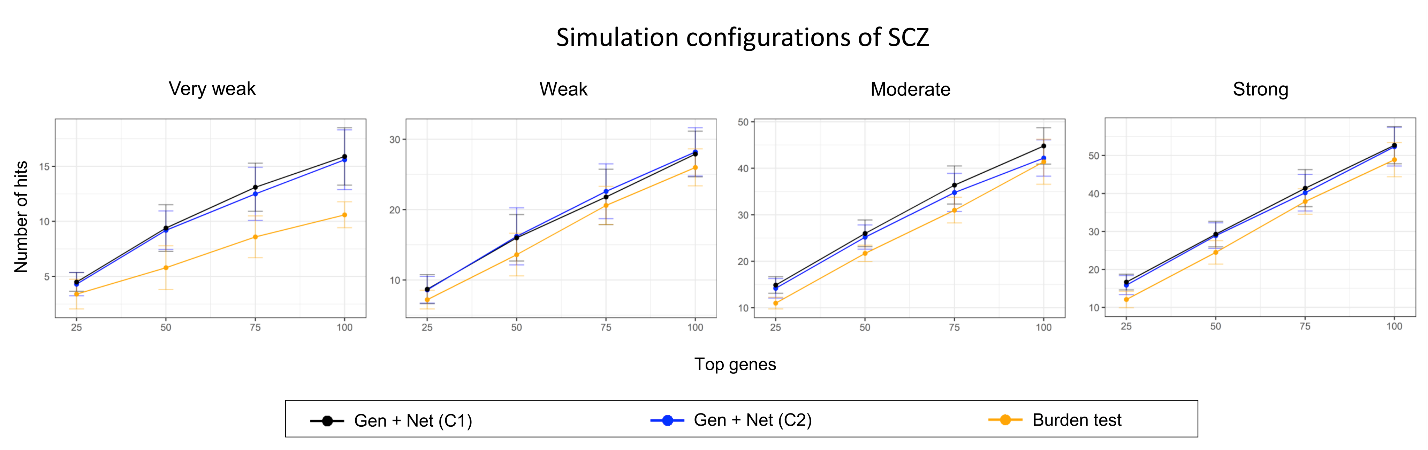 |

**S11 Fig. The performance of IGSP based on different types of network connectivity in network-based scoring.**
