# Supplementary material for: Integrated rare variant-based risk gene prioritization in disease case-control sequencing studies
Source: PLoS Genet. 2017 Dec 27;13(12):e1007142. doi: 10.1371/journal.pgen.1007142 (PMC5760082; doi:10.1371/journal.pgen.1007142)
Supplement: S13 Fig — Association signals in null are gene association signals of which stronger signals do not tend to aggregate at risk genes. Association signals in null in simulation (A, B, C and D) are obtained through randomly assigning simulated gene association signals of all genes, including risk and non-risk genes, to themselves. Association signals in null in a real application (E) are obtained through permuting the original disease status label and randomly assigning the resultant disease association signals to genes. The scores of top 100 high scoring genes in IGSP are shown in the figures. 100 trials of IGSP given association signals in null are conducted and the error bars represent standard deviation. The parameter setup of IGSP for simulation in A, B, C and D and the real application in E: integrated scoring with both network and phenotype, x (2), a (0.1), b (1), and principal components in phenotype scoring (PC 2 and 3). (A) Simulation with 147 CHD genes with a setup of “Very weak” association signal strength. (B) Simulation with 147 CHD genes with a setup of “Weak” association signal strength. (C) Simulation with 147 CHD genes with a setup of “Moderate” association signal strength. (D) Simulation with 147 CHD genes with a setup of “Strong” association signal strength. (E) A real application in the case study of CHD. (DOCX) [file pgen.1007142.s013.docx]

| A  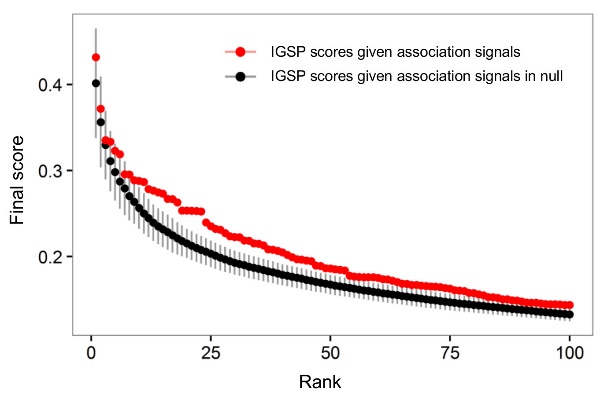 | B  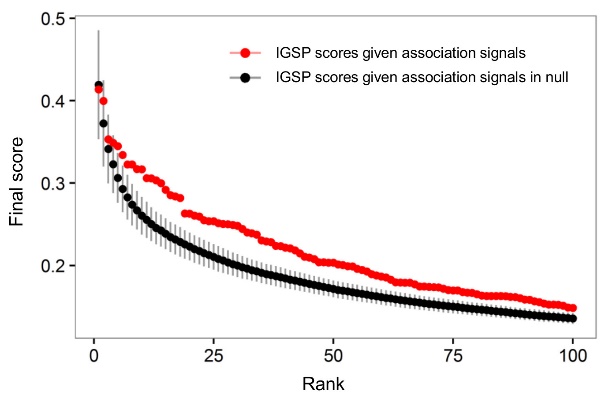 |
| --- | --- |
| C  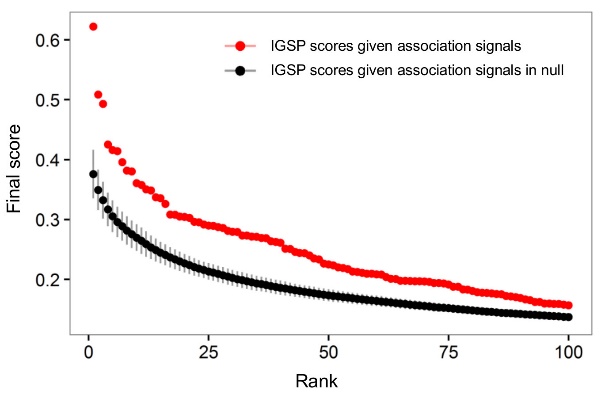 | D  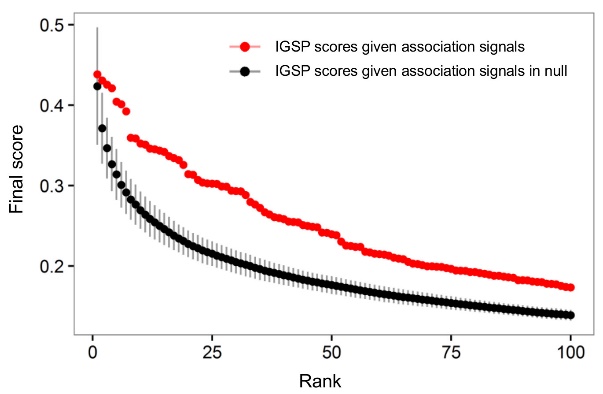 |
| E  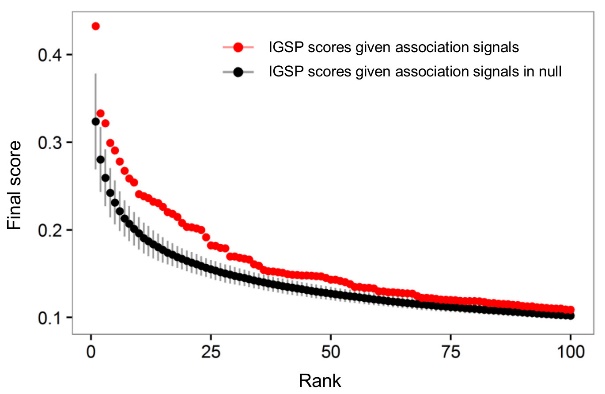 |  |

**S13 Fig. Distribution of top IGSP scores given association signals in null as negative control.**
