# Supplementary material for: Integrated rare variant-based risk gene prioritization in disease case-control sequencing studies
Source: PLoS Genet. 2017 Dec 27;13(12):e1007142. doi: 10.1371/journal.pgen.1007142 (PMC5760082; doi:10.1371/journal.pgen.1007142)
Supplement: S1 Table — (DOCX) [file pgen.1007142.s022.docx]

| **S1 Table. Risk genes of 5 human diseases from OMIM.** | |
| --- | --- |
| Disease | Risk genes |
| Breast cancer | *PPM1D, RB1CC1, KRAS, AKT1, CHEK2, TSG101, BACH1, BARD1, PIK3CA, PHB, HMMR, NQO2, TP53, BRIP1, ATM, ESR1, XRCC3, PALB2, CDS1* |
| Schizophrenia | *SLC1A1, COMT, DAO, RTN4R, DISC1, AKT1, SHANK3, CHI3L1, NRXN1, HTR2A, CHRNA7, DRD3, NRG1, MTHFR, PRODH, DAOA, APOL2, APOL4, PRODH2* |
| Tetralogy of Fallot | *GATA4, JAG1, TBX1, ZFPM2, GATA6, NKX2-5, GDF1* |
| Systemic lupus erythematosus | *PTPN22, ITGAM, DNASE1, TLR5, PDCD1, DNASE1L3, STAT4, TREX1, CTLA4, IRF5, CR2, FCGR2B* |
| Type 2 Diabetes | *AKT2, TCF7L2, PAX4, KCNJ11, PPARG, GPD2, TCF4, PDX1, MTNR1B* |

Note: Underlined genes do not have a MP annotation.
