# Supplementary material for: Integrated rare variant-based risk gene prioritization in disease case-control sequencing studies
Source: PLoS Genet. 2017 Dec 27;13(12):e1007142. doi: 10.1371/journal.pgen.1007142 (PMC5760082; doi:10.1371/journal.pgen.1007142)
Supplement: S2 Table — Those genes were obtained from Sifrim et al [26]. (DOCX) [file pgen.1007142.s023.docx]

| **S2 Table. 147 putative CHD genes that can be scored by network and phenotype**. | | | | | |
| --- | --- | --- | --- | --- | --- |
| Ensembl ID | Gene symbol | Ensembl ID | Gene symbol | Ensembl ID | Gene symbol |
| ENSG00000122863 | *CHST3* | ENSG00000008441 | *NFIX* | ENSG00000080503 | *SMARCA2* |
| ENSG00000174705 | *SH3PXD2B* | ENSG00000106799 | *TGFBR1* | ENSG00000139219 | *COL2A1* |
| ENSG00000125398 | *SOX9* | ENSG00000171316 | *CHD7* | ENSG00000108557 | *RAI1* |
| ENSG00000166813 | *KIF7* | ENSG00000179915 | *NRXN1* | ENSG00000105429 | *MEGF8* |
| ENSG00000092054 | *MYH7* | ENSG00000176842 | *IRX5* | ENSG00000113971 | *NPHP3* |
| ENSG00000133703 | *KRAS* | ENSG00000075624 | *ACTB* | ENSG00000004399 | *PLXND1* |
| ENSG00000092621 | *PHGDH* | ENSG00000159459 | *UBR1* | ENSG00000213341 | *CHUK* |
| ENSG00000110395 | *CBL* | ENSG00000157764 | *BRAF* | ENSG00000136574 | *GATA4* |
| ENSG00000077092 | *RARB* | ENSG00000156925 | *ZIC3* | ENSG00000068024 | *HDAC4* |
| ENSG00000164190 | *NIPBL* | ENSG00000147257 | *GPC3* | ENSG00000106571 | *GLI3* |
| ENSG00000125863 | *MKKS* | ENSG00000162337 | *LRP5* | ENSG00000143622 | *RIT1* |
| ENSG00000011143 | *MKS1* | ENSG00000100393 | *EP300* | ENSG00000174775 | *HRAS* |
| ENSG00000127980 | *PEX1* | ENSG00000114251 | *WNT5A* | ENSG00000173040 | *EVC2* |
| ENSG00000163453 | *IGFBP7* | ENSG00000008710 | *PKD1* | ENSG00000101384 | *JAG1* |
| ENSG00000038295 | *TLL1* | ENSG00000185551 | *NR2F2* | ENSG00000198003 | *CCDC151* |
| ENSG00000183337 | *BCOR* | ENSG00000069431 | *ABCC9* | ENSG00000141519 | *CCDC40* |
| ENSG00000164751 | *PEX2* | ENSG00000114739 | *ACVR2B* | ENSG00000138435 | *CHRNA1* |
| ENSG00000256061 | *DYX1C1* | ENSG00000163513 | *TGFBR2* | ENSG00000013573 | *DDX11* |
| ENSG00000181449 | *SOX2* | ENSG00000108821 | *COL1A1* | ENSG00000184634 | *MED12* |
| ENSG00000101126 | *ADNP* | ENSG00000159251 | *ACTC1* | ENSG00000145075 | *CCDC39* |
| ENSG00000124356 | *STAMBP* | ENSG00000089225 | *TBX5* | ENSG00000179295 | *PTPN11* |
| ENSG00000196712 | *NF1* | ENSG00000198707 | *CEP290* | ENSG00000115904 | *SOS1* |
| ENSG00000169032 | *MAP2K1* | ENSG00000122691 | *TWIST1* | ENSG00000197496 | *SLC2A10* |
| ENSG00000171456 | *ASXL1* | ENSG00000166949 | *SMAD3* | ENSG00000147050 | *KDM6A* |
| ENSG00000165671 | *NSD1* | ENSG00000112210 | *RAB23* | ENSG00000073584 | *SMARCE1* |
| ENSG00000156650 | *KAT6B* | ENSG00000148400 | *NOTCH1* | ENSG00000112367 | *FIG4* |
| ENSG00000127616 | *SMARCA4* | ENSG00000101871 | *MID1* | ENSG00000141448 | *GATA6* |
| ENSG00000131196 | *NFATC1* | ENSG00000072840 | *EVC* | ENSG00000106692 | *FKTN* |
| ENSG00000183287 | *CCBE1* | ENSG00000215193 | *PEX26* | ENSG00000137868 | *STRA6* |
| ENSG00000141646 | *SMAD4* | ENSG00000176692 | *FOXC2* | ENSG00000167548 | *KMT2D* |
| ENSG00000164442 | *CITED2* | ENSG00000172893 | *DHCR7* | ENSG00000152217 | *SETBP1* |
| ENSG00000168267 | *PTF1A* | ENSG00000103241 | *FOXF1* | ENSG00000134250 | *NOTCH2* |
| ENSG00000156574 | *NODAL* | ENSG00000108883 | *EFTUD2* | ENSG00000137601 | *NEK1* |
| ENSG00000126934 | *MAP2K2* | ENSG00000175387 | *SMAD2* | ENSG00000138829 | *FBN2* |
| ENSG00000066468 | *FGFR2* | ENSG00000169554 | *ZEB2* | ENSG00000111341 | *MGP* |
| ENSG00000157933 | *SKI* | ENSG00000183072 | *NKX2-5* | ENSG00000180053 | *NKX2-6* |
| ENSG00000143768 | *LEFTY2* | ENSG00000102974 | *CTCF* | ENSG00000139197 | *PEX5* |
| ENSG00000169105 | *CHST14* | ENSG00000157911 | *PEX10* | ENSG00000107796 | *ACTA2* |
| ENSG00000135111 | *TBX3* | ENSG00000213281 | *NRAS* | ENSG00000169071 | *ROR2* |
| ENSG00000055208 | *TAB2* | ENSG00000140092 | *FBLN5* | ENSG00000101115 | *SALL4* |
| ENSG00000168282 | *MGAT2* | ENSG00000181090 | *EHMT1* | ENSG00000136698 | *CFC1* |
| ENSG00000133392 | *MYH11* | ENSG00000251322 | *SHANK3* | ENSG00000118762 | *PKD2* |
| ENSG00000168542 | *COL3A1* | ENSG00000169946 | *ZFPM2* | ENSG00000103449 | *SALL1* |
| ENSG00000184058 | *TBX1* | ENSG00000140718 | *FTO* | ENSG00000117713 | *ARID1A* |
| ENSG00000081479 | *LRP2* | ENSG00000184675 | *AMER1* | ENSG00000084073 | *ZMPSTE24* |
| ENSG00000152661 | *GJA1* | ENSG00000164532 | *TBX20* | ENSG00000136068 | *FLNB* |
| ENSG00000004961 | *HCCS* | ENSG00000108061 | *SHOC2* | ENSG00000099956 | *SMARCB1* |
| ENSG00000005339 | *CREBBP* | ENSG00000126458 | *RRAS* |  |  |
| ENSG00000168056 | *LTBP3* | ENSG00000054598 | *FOXC1* |  |  |
| ENSG00000171320 | *ESCO2* | ENSG00000008196 | *TFAP2B* |  |  |
|  | | | | | |
