# Supplementary material for: Integrated rare variant-based risk gene prioritization in disease case-control sequencing studies
Source: PLoS Genet. 2017 Dec 27;13(12):e1007142. doi: 10.1371/journal.pgen.1007142 (PMC5760082; doi:10.1371/journal.pgen.1007142)
Supplement: S3 Table — Those genes were obtained from Malacards [57]. (DOCX) [file pgen.1007142.s024.docx]

| **Table S3. 193 putative schizophrenia genes that can be scored by network and phenotype.** | | | | | |
| --- | --- | --- | --- | --- | --- |
| Ensembl ID | Gene symbol | Ensembl ID | Gene symbol | Ensembl ID | Gene symbol |
| ENSG00000083807 | *SLC27A5* | ENSG00000110436 | *SLC1A2* | ENSG00000273079 | *GRIN2B* |
| ENSG00000163288 | *GABRB1* | ENSG00000178127 | *NDUFV2* | ENSG00000149557 | *FEZ1* |
| ENSG00000078018 | *MAP2* | ENSG00000133048 | *CHI3L1* | ENSG00000155511 | *GRIA1* |
| ENSG00000105866 | *SP4* | ENSG00000168539 | *CHRM1* | ENSG00000113645 | *WWC1* |
| ENSG00000132639 | *SNAP25* | ENSG00000179915 | *NRXN1* | ENSG00000163873 | *GRIK3* |
| ENSG00000103546 | *SLC6A2* | ENSG00000160716 | *CHRNB2* | ENSG00000069696 | *DRD4* |
| ENSG00000169567 | *HINT1* | ENSG00000101180 | *HRH3* | ENSG00000167720 | *SRR* |
| ENSG00000135312 | *HTR1B* | ENSG00000108576 | *SLC6A4* | ENSG00000165646 | *SLC18A2* |
| ENSG00000162951 | *LRRTM1* | ENSG00000171533 | *MAP6* | ENSG00000133636 | *NTS* |
| ENSG00000092964 | *DPYSL2* | ENSG00000116032 | *GRIN3B* | ENSG00000143603 | *KCNN3* |
| ENSG00000104327 | *CALB1* | ENSG00000154146 | *NRGN* | ENSG00000107130 | *NCS1* |
| ENSG00000176697 | *BDNF* | ENSG00000117152 | *RGS4* | ENSG00000172179 | *PRL* |
| ENSG00000185737 | *NRG3* | ENSG00000166206 | *GABRB3* | ENSG00000189221 | *MAOA* |
| ENSG00000076356 | *PLXNA2* | ENSG00000089250 | *NOS1* | ENSG00000128271 | *ADORA2A* |
| ENSG00000147246 | *HTR2C* | ENSG00000174871 | *CNIH2* | ENSG00000100033 | *PRODH* |
| ENSG00000179388 | *EGR3* | ENSG00000172137 | *CALB2* | ENSG00000163394 | *CCKAR* |
| ENSG00000120910 | *PPP3CC* | ENSG00000100151 | *PICK1* | ENSG00000106688 | *SLC1A1* |
| ENSG00000118432 | *CNR1* | ENSG00000147894 | *C9orf72* | ENSG00000169676 | *DRD5* |
| ENSG00000161610 | *HCRT* | ENSG00000175344 | *CHRNA7* | ENSG00000171189 | *GRIK1* |
| ENSG00000036565 | *SLC18A1* | ENSG00000186297 | *GABRA5* | ENSG00000139287 | *TPH2* |
| ENSG00000161509 | *GRIN2C* | ENSG00000135069 | *PSAT1* | ENSG00000124493 | *GRM4* |
| ENSG00000109158 | *GABRA4* | ENSG00000140557 | *ST8SIA2* | ENSG00000082458 | *DLG3* |
| ENSG00000105695 | *MAG* | ENSG00000142208 | *AKT1* | ENSG00000184634 | *MED12* |
| ENSG00000189056 | *RELN* | ENSG00000183454 | *GRIN2A* | ENSG00000069535 | *MAOB* |
| ENSG00000142319 | *SLC6A3* | ENSG00000145794 | *MEGF10* | ENSG00000140505 | *CYP1A2* |
| ENSG00000185666 | *SYN3* | ENSG00000166862 | *CACNG2* | ENSG00000149403 | *GRIK4* |
| ENSG00000164418 | *GRIK2* | ENSG00000120907 | *ADRA1A* | ENSG00000079215 | *SLC1A3* |
| ENSG00000174469 | *CNTNAP2* | ENSG00000157168 | *NRG1* | ENSG00000113327 | *GABRG2* |
| ENSG00000158748 | *HTR6* | ENSG00000104888 | *SLC17A7* | ENSG00000105737 | *GRIK5* |
| ENSG00000101204 | *CHRNA4* | ENSG00000151790 | *TDO2* | ENSG00000007372 | *PAX6* |
| ENSG00000106069 | *CHN2* | ENSG00000111602 | *TIMELESS* | ENSG00000152822 | *GRM1* |
| ENSG00000157103 | *SLC6A1* | ENSG00000165970 | *SLC6A5* | ENSG00000185652 | *NTF3* |
| ENSG00000122585 | *NPY* | ENSG00000151150 | *ANK3* | ENSG00000130816 | *DNMT1* |
| ENSG00000123454 | *DBH* | ENSG00000254647 | *INS* | ENSG00000091664 | *SLC17A6* |
| ENSG00000104290 | *FZD3* | ENSG00000141401 | *IMPA2* | ENSG00000130643 | *CALY* |
| ENSG00000047579 | *DTNBP1* | ENSG00000122966 | *CIT* | ENSG00000172020 | *GAP43* |
| ENSG00000166736 | *HTR3A* | ENSG00000162946 | *DISC1* | ENSG00000198822 | *GRM3* |
| ENSG00000204681 | *GABBR1* | ENSG00000177000 | *MTHFR* | ENSG00000128683 | *GAD1* |
| ENSG00000120251 | *GRIA2* | ENSG00000166603 | *MC4R* | ENSG00000113749 | *HRH2* |
| ENSG00000131771 | *PPP1R1B* | ENSG00000164082 | *GRM2* | ENSG00000102468 | *HTR2A* |
| ENSG00000148053 | *NTRK2* | ENSG00000151322 | *NPAS3* | ENSG00000099904 | *ZDHHC8* |
| ENSG00000146383 | *TAAR6* | ENSG00000102245 | *CD40LG* | ENSG00000040608 | *RTN4R* |
| ENSG00000153234 | *NR4A2* | ENSG00000179546 | *HTR1D* | ENSG00000110887 | *DAO* |
| ENSG00000007168 | *PAFAH1B1* | ENSG00000184845 | *DRD1* | ENSG00000180176 | *TH* |
| ENSG00000100427 | *MLC1* | ENSG00000170485 | *NPAS2* | ENSG00000182771 | *GRID1* |
| ENSG00000151067 | *CACNA1C* | ENSG00000137726 | *FXYD6* | ENSG00000132693 | *CRP* |
| ENSG00000134121 | *CHL1* | ENSG00000100385 | *IL2RB* | ENSG00000148680 | *HTR7* |
| ENSG00000093010 | *COMT* | ENSG00000135577 | *NMBR* | ENSG00000174437 | *ATP2A2* |
| ENSG00000178568 | *ERBB4* | ENSG00000115310 | *RTN4* | ENSG00000168038 | *ULK4* |
| ENSG00000176884 | *GRIN1* | ENSG00000086205 | *FOLH1* | ENSG00000160299 | *PCNT* |
| ENSG00000106018 | *VIPR2* | ENSG00000133794 | *ARNTL* | ENSG00000149295 | *DRD2* |
| ENSG00000100362 | *PVALB* | ENSG00000111664 | *GNB3* | ENSG00000152578 | *GRIA4* |
| ENSG00000136750 | *GAD2* | ENSG00000145920 | *CPLX2* | ENSG00000187094 | *CCK* |
| ENSG00000100528 | *CNIH1* | ENSG00000204301 | *NOTCH4* | ENSG00000157005 | *SST* |
| ENSG00000152332 | *UHMK1* | ENSG00000251322 | *SHANK3* | ENSG00000178394 | *HTR1A* |
| ENSG00000184058 | *TBX1* | ENSG00000129167 | *TPH1* | ENSG00000150867 | *PIP4K2A* |
| ENSG00000184588 | *PDE4B* | ENSG00000168959 | *GRM5* | ENSG00000125675 | *GRIA3* |
| ENSG00000082701 | *GSK3B* | ENSG00000189058 | *APOD* | ENSG00000116711 | *PLA2G4A* |
| ENSG00000173786 | *CNP* | ENSG00000168314 | *MOBP* | ENSG00000141404 | *GNAL* |
| ENSG00000151577 | *DRD3* | ENSG00000150594 | *ADRA2A* | ENSG00000249751 | *ECSCR* |
| ENSG00000075213 | *SEMA3A* | ENSG00000196517 | *SLC6A9* | ENSG00000078142 | *PIK3C3* |
| ENSG00000197971 | *MBP* | ENSG00000143416 | *SELENBP1* | ENSG00000112531 | *QKI* |
| ENSG00000184381 | *PLA2G6* | ENSG00000080503 | *SMARCA2* | ENSG00000132535 | *DLG4* |
| ENSG00000142192 | *APP* | ENSG00000099889 | *ARVCF* |  |  |
| ENSG00000163110 | *PDLIM5* | ENSG00000169836 | *TACR3* |  |  |
|  | | | | | |
