# Supplementary material for: Integrated rare variant-based risk gene prioritization in disease case-control sequencing studies
Source: PLoS Genet. 2017 Dec 27;13(12):e1007142. doi: 10.1371/journal.pgen.1007142 (PMC5760082; doi:10.1371/journal.pgen.1007142)
Supplement: S4 Table — (DOCX) [file pgen.1007142.s025.docx]

| **S4 Table. The setup of *P*-values of risk genes in simulation.** | | | | | |
| --- | --- | --- | --- | --- | --- |
| Association signal strength of risk genes^1^ | Risk genes^2^ | *P* <= 0.01^3^ | 0.01 < *P* <= 0.05^3^ | 0.05 < *P* <= 0.25^3^ | *P* > 0.25^3^ |
| Strong | Proportion | 0.4 | 0.5 | 0.05 | 0.05 |
|  | 147 CHD genes | 59 | 74 | 7 | 7 |
|  | 193 SCZ genes | 77 | 96 | 10 | 10 |
| Moderate | Proportion | 0.3 | 0.4 | 0.2 | 0.1 |
|  | 147 CHD genes | 44 | 59 | 29 | 15 |
|  | 193 SCZ genes | 58 | 77 | 39 | 19 |
| Weak | Proportion | 0.15 | 0.25 | 0.4 | 0.2 |
|  | 147 CHD genes | 22 | 37 | 59 | 29 |
|  | 193 SCZ genes | 29 | 48 | 77 | 39 |
| Very weak | Proportion | 0.05 | 0.2 | 0.5 | 0.25 |
|  | 147 CHD genes | 7 | 29 | 74 | 37 |
|  | 193 SCZ genes | 10 | 39 | 96 | 48 |
| Notes:   1. The labels “Strong”, “Moderate”, “Weak” and “Very Weak” define four simulation configurations of different association signal strength of risk genes on a relative rather than absolute basis. 2. Risk genes in simulation are putative risk genes from other resources (**S2** **Table** and **S3** **Table**). 3. Risk genes are randomly assigned a *P*-value in the specified *P*-value range. | | | | | |
