# Supplementary material for: Integrated rare variant-based risk gene prioritization in disease case-control sequencing studies
Source: PLoS Genet. 2017 Dec 27;13(12):e1007142. doi: 10.1371/journal.pgen.1007142 (PMC5760082; doi:10.1371/journal.pgen.1007142)
Supplement: S5 Table — (DOCX) [file pgen.1007142.s026.docx]

| **S5 Table. Top 200 genes based on IGSP integrated scoring for the case-control WES study of CHD in 22q11.2DS.** | | | | | | |
| --- | --- | --- | --- | --- | --- | --- |
| Rank | EID | Gene symbol | Final score | Association score | Average Net score | Average Phe score |
| 1 | ENSG00000066468 | *FGFR2* | 0.433 | 2.203 | 0.981 | 0.988 |
| 2 | ENSG00000111799 | *COL12A1* | 0.333 | 2.915 | 0.580 | 0.659 |
| 3 | ENSG00000155657 | *TTN* | 0.322 | 1.689 | 0.958 | 0.925 |
| 4 | ENSG00000083444 | *PLOD1* | 0.299 | 2.858 | 0.652 | 0.533 |
| 5 | ENSG00000081248 | *CACNA1S* | 0.291 | 1.714 | 0.814 | 0.935 |
| 6 | ENSG00000179915 | *NRXN1* | 0.278 | 2.203 | 0.843 | 0.640 |
| 7 | ENSG00000173402 | *DAG1* | 0.268 | 1.879 | 0.683 | 0.880 |
| 8 | ENSG00000157168 | *NRG1* | 0.259 | 1.483 | 0.932 | 0.916 |
| 9 | ENSG00000141736 | *ERBB2* | 0.254 | 1.324 | 0.913 | 0.959 |
| 10 | ENSG00000104313 | *EYA1* | 0.241 | 1.558 | 0.786 | 0.959 |
| 11 | ENSG00000103126 | *AXIN1* | 0.239 | 1.240 | 0.972 | 0.935 |
| 12 | ENSG00000107731 | *UNC5B* | 0.236 | 1.879 | 0.749 | 0.662 |
| 13 | ENSG00000131697 | *NPHP4* | 0.232 | 2.970 | 0.486 | 0.488 |
| 14 | ENSG00000077782 | *FGFR1* | 0.231 | 1.240 | 0.898 | 0.997 |
| 15 | ENSG00000172037 | *LAMB2* | 0.226 | 1.843 | 0.864 | 0.592 |
| 16 | ENSG00000053747 | *LAMA3* | 0.220 | 1.943 | 0.749 | 0.638 |
| 17 | ENSG00000183963 | *SMTN* | 0.218 | 2.529 | 0.527 | 0.542 |
| 18 | ENSG00000157766 | *ACAN* | 0.215 | 1.461 | 0.732 | 0.884 |
| 19 | ENSG00000020181 | *GPR124* | 0.208 | 1.302 | 0.827 | 0.836 |
| 20 | ENSG00000138802 | *SEC24B* | 0.204 | 1.558 | 0.686 | 0.816 |
| 21 | ENSG00000169071 | *ROR2* | 0.203 | 1.240 | 0.812 | 0.982 |
| 22 | ENSG00000165699 | *TSC1* | 0.202 | 1.054 | 0.946 | 0.965 |
| 23 | ENSG00000175054 | *ATR* | 0.200 | 1.367 | 0.711 | 0.883 |
| 24 | ENSG00000179218 | *CALR* | 0.192 | 1.240 | 0.929 | 0.776 |
| 25 | ENSG00000074047 | *GLI2* | 0.182 | 0.911 | 0.951 | 0.990 |
| 26 | ENSG00000062822 | *POLD1* | 0.182 | 1.884 | 0.652 | 0.569 |
| 27 | ENSG00000183072 | *NKX2-5* | 0.180 | 0.925 | 0.974 | 0.933 |
| 28 | ENSG00000130702 | *LAMA5* | 0.179 | 1.271 | 0.857 | 0.709 |
| 29 | ENSG00000143537 | *ADAM15* | 0.170 | 1.879 | 0.586 | 0.529 |
| 30 | ENSG00000101144 | *BMP7* | 0.170 | 0.925 | 0.945 | 0.914 |
| 31 | ENSG00000142871 | *CYR61* | 0.168 | 1.240 | 0.809 | 0.710 |
| 32 | ENSG00000138829 | *FBN2* | 0.167 | 1.168 | 0.875 | 0.715 |
| 33 | ENSG00000196712 | *NF1* | 0.166 | 0.925 | 0.979 | 0.891 |
| 34 | ENSG00000099250 | *NRP1* | 0.161 | 1.240 | 0.670 | 0.882 |
| 35 | ENSG00000125863 | *MKKS* | 0.159 | 1.054 | 0.887 | 0.832 |
| 36 | ENSG00000136574 | *GATA4* | 0.154 | 0.925 | 0.816 | 0.968 |
| 37 | ENSG00000058085 | *LAMC2* | 0.153 | 1.558 | 0.546 | 0.660 |
| 38 | ENSG00000173991 | *TCAP* | 0.153 | 1.558 | 0.875 | 0.419 |
| 39 | ENSG00000198626 | *RYR2* | 0.152 | 1.033 | 0.845 | 0.777 |
| 40 | ENSG00000146085 | *MUT* | 0.151 | 1.879 | 0.421 | 0.718 |
| 41 | ENSG00000112659 | *CUL9* | 0.150 | 1.714 | 0.580 | 0.523 |
| 42 | ENSG00000046889 | *PREX2* | 0.149 | 1.879 | 0.542 | 0.442 |
| 43 | ENSG00000120725 | *SIL1* | 0.149 | 2.203 | 0.355 | 0.514 |
| 44 | ENSG00000114948 | *ADAM23* | 0.148 | 1.558 | 0.617 | 0.596 |
| 45 | ENSG00000099139 | *PCSK5* | 0.148 | 0.925 | 0.849 | 0.946 |
| 46 | ENSG00000008710 | *PKD1* | 0.148 | 1.187 | 0.492 | 0.993 |
| 47 | ENSG00000196878 | *LAMB3* | 0.147 | 1.151 | 0.780 | 0.681 |
| 48 | ENSG00000065361 | *ERBB3* | 0.147 | 0.925 | 0.787 | 0.939 |
| 49 | ENSG00000136153 | *LMO7* | 0.145 | 1.879 | 0.400 | 0.657 |
| 50 | ENSG00000157087 | *ATP2B2* | 0.143 | 1.391 | 0.587 | 0.690 |
| 51 | ENSG00000139567 | *ACVRL1* | 0.143 | 0.925 | 0.918 | 0.850 |
| 52 | ENSG00000111199 | *TRPV4* | 0.142 | 1.558 | 0.936 | 0.403 |
| 53 | ENSG00000128052 | *KDR* | 0.141 | 0.881 | 0.939 | 0.812 |
| 54 | ENSG00000060718 | *COL11A1* | 0.138 | 1.112 | 0.702 | 0.777 |
| 55 | ENSG00000096433 | *ITPR3* | 0.135 | 1.524 | 0.759 | 0.460 |
| 56 | ENSG00000115310 | *RTN4* | 0.135 | 1.303 | 0.821 | 0.502 |
| 57 | ENSG00000167768 | *KRT1* | 0.134 | 1.240 | 0.797 | 0.540 |
| 58 | ENSG00000188906 | *LRRK2* | 0.134 | 1.151 | 0.989 | 0.489 |
| 59 | ENSG00000157212 | *PAXIP1* | 0.133 | 0.925 | 0.769 | 0.860 |
| 60 | ENSG00000179776 | *CDH5* | 0.130 | 1.240 | 0.747 | 0.551 |
| 61 | ENSG00000136944 | *LMX1B* | 0.130 | 0.925 | 0.734 | 0.918 |
| 62 | ENSG00000198844 | *ARHGEF15* | 0.129 | 1.558 | 0.495 | 0.585 |
| 63 | ENSG00000148498 | *PARD3* | 0.129 | 1.240 | 0.597 | 0.718 |
| 64 | ENSG00000064309 | *CDON* | 0.128 | 0.931 | 0.692 | 0.932 |
| 65 | ENSG00000143816 | *WNT9A* | 0.128 | 1.240 | 0.727 | 0.554 |
| 66 | ENSG00000167910 | *CYP7A1* | 0.128 | 1.240 | 0.686 | 0.544 |
| 67 | ENSG00000096717 | *SIRT1* | 0.127 | 0.743 | 0.935 | 0.835 |
| 68 | ENSG00000100644 | *HIF1A* | 0.125 | 0.743 | 0.936 | 0.810 |
| 69 | ENSG00000105664 | *COMP* | 0.123 | 1.240 | 0.805 | 0.451 |
| 70 | ENSG00000102858 | *MGRN1* | 0.123 | 1.054 | 0.608 | 0.806 |
| 71 | ENSG00000185359 | *HGS* | 0.122 | 0.925 | 0.637 | 0.936 |
| 72 | ENSG00000166341 | *DCHS1* | 0.121 | 1.105 | 0.525 | 0.823 |
| 73 | ENSG00000174842 | *GLMN* | 0.121 | 0.925 | 0.758 | 0.797 |
| 74 | ENSG00000125124 | *BBS2* | 0.120 | 0.925 | 0.888 | 0.633 |
| 75 | ENSG00000180318 | *ALX1* | 0.120 | 0.757 | 0.895 | 0.862 |
| 76 | ENSG00000196876 | *SCN8A* | 0.120 | 0.925 | 0.663 | 0.863 |
| 77 | ENSG00000120156 | *TEK* | 0.119 | 0.925 | 0.886 | 0.714 |
| 78 | ENSG00000198400 | *NTRK1* | 0.119 | 0.925 | 0.847 | 0.713 |
| 79 | ENSG00000180370 | *PAK2* | 0.119 | 0.925 | 0.834 | 0.620 |
| 80 | ENSG00000172071 | *EIF2AK3* | 0.119 | 0.925 | 0.921 | 0.591 |
| 81 | ENSG00000186868 | *MAPT* | 0.119 | 0.925 | 0.746 | 0.785 |
| 82 | ENSG00000154342 | *WNT3A* | 0.118 | 0.612 | 0.914 | 0.967 |
| 83 | ENSG00000132855 | *ANGPTL3* | 0.117 | 1.558 | 0.726 | 0.351 |
| 84 | ENSG00000189056 | *RELN* | 0.117 | 0.720 | 0.958 | 0.816 |
| 85 | ENSG00000030304 | *MUSK* | 0.116 | 0.988 | 0.645 | 0.756 |
| 86 | ENSG00000114251 | *WNT5A* | 0.116 | 0.612 | 0.988 | 0.948 |
| 87 | ENSG00000167880 | *EVPL* | 0.115 | 2.243 | 0.614 | 0.158 |
| 88 | ENSG00000156970 | *BUB1B* | 0.115 | 0.925 | 0.850 | 0.618 |
| 89 | ENSG00000196782 | *MAML3* | 0.114 | 1.240 | 0.418 | 0.761 |
| 90 | ENSG00000127831 | *VIL1* | 0.113 | 2.198 | 0.851 | 0.156 |
| 91 | ENSG00000138336 | *TET1* | 0.113 | 1.558 | 0.555 | 0.473 |
| 92 | ENSG00000075891 | *PAX2* | 0.113 | 0.612 | 0.953 | 0.901 |
| 93 | ENSG00000164828 | *SUN1* | 0.112 | 1.879 | 0.201 | 0.800 |
| 94 | ENSG00000148408 | *CACNA1B* | 0.112 | 1.176 | 0.730 | 0.485 |
| 95 | ENSG00000198707 | *CEP290* | 0.111 | 0.965 | 0.544 | 0.861 |
| 96 | ENSG00000080573 | *COL5A3* | 0.111 | 1.634 | 0.599 | 0.340 |
| 97 | ENSG00000167642 | *SPINT2* | 0.110 | 1.240 | 0.509 | 0.701 |
| 98 | ENSG00000146648 | *EGFR* | 0.110 | 0.612 | 0.972 | 0.975 |
| 99 | ENSG00000129514 | *FOXA1* | 0.109 | 1.240 | 0.960 | 0.371 |
| 100 | ENSG00000159184 | *HOXB13* | 0.109 | 0.925 | 0.853 | 0.612 |
| 101 | ENSG00000184500 | *PROS1* | 0.109 | 0.925 | 0.879 | 0.593 |
| 102 | ENSG00000139687 | *RB1* | 0.109 | 0.612 | 0.902 | 0.959 |
| 103 | ENSG00000017427 | *IGF1* | 0.109 | 0.612 | 0.984 | 0.889 |
| 104 | ENSG00000104738 | *MCM4* | 0.108 | 1.879 | 0.416 | 0.389 |
| 105 | ENSG00000103056 | *SMPD3* | 0.107 | 1.558 | 0.240 | 0.840 |
| 106 | ENSG00000183023 | *SLC8A1* | 0.107 | 0.925 | 0.590 | 0.881 |
| 107 | ENSG00000139618 | *BRCA2* | 0.107 | 0.803 | 0.742 | 0.753 |
| 108 | ENSG00000150938 | *CRIM1* | 0.106 | 1.240 | 0.474 | 0.638 |
| 109 | ENSG00000165731 | *RET* | 0.106 | 0.745 | 0.683 | 0.904 |
| 110 | ENSG00000125845 | *BMP2* | 0.106 | 0.612 | 0.918 | 0.909 |
| 111 | ENSG00000086991 | *NOX4* | 0.105 | 1.879 | 0.367 | 0.447 |
| 112 | ENSG00000129170 | *CSRP3* | 0.105 | 0.925 | 0.722 | 0.676 |
| 113 | ENSG00000164458 | *T* | 0.105 | 0.742 | 0.661 | 0.968 |
| 114 | ENSG00000181449 | *SOX2* | 0.105 | 0.612 | 0.860 | 0.961 |
| 115 | ENSG00000100324 | *TAB1* | 0.105 | 1.054 | 0.509 | 0.779 |
| 116 | ENSG00000112715 | *VEGFA* | 0.105 | 0.612 | 0.825 | 0.985 |
| 117 | ENSG00000163638 | *ADAMTS9* | 0.105 | 0.945 | 0.563 | 0.846 |
| 118 | ENSG00000144857 | *BOC* | 0.105 | 1.054 | 0.556 | 0.760 |
| 119 | ENSG00000151067 | *CACNA1C* | 0.105 | 0.748 | 0.818 | 0.834 |
| 120 | ENSG00000085276 | *MECOM* | 0.104 | 1.067 | 0.479 | 0.867 |
| 121 | ENSG00000179981 | *TSHZ1* | 0.104 | 1.240 | 0.333 | 0.843 |
| 122 | ENSG00000198363 | *ASPH* | 0.104 | 1.558 | 0.370 | 0.542 |
| 123 | ENSG00000141837 | *CACNA1A* | 0.103 | 0.862 | 0.655 | 0.752 |
| 124 | ENSG00000171532 | *NEUROD2* | 0.103 | 0.925 | 0.868 | 0.513 |
| 125 | ENSG00000142798 | *HSPG2* | 0.103 | 0.676 | 0.766 | 0.955 |
| 126 | ENSG00000107831 | *FGF8* | 0.103 | 0.612 | 0.807 | 0.985 |
| 127 | ENSG00000072134 | *EPN2* | 0.102 | 1.558 | 0.234 | 0.851 |
| 128 | ENSG00000112559 | *MDFI* | 0.102 | 0.612 | 0.905 | 0.830 |
| 129 | ENSG00000124216 | *SNAI1* | 0.102 | 0.612 | 0.777 | 0.935 |
| 130 | ENSG00000097046 | *CDC7* | 0.102 | 1.054 | 0.658 | 0.548 |
| 131 | ENSG00000145194 | *ECE2* | 0.101 | 0.988 | 0.647 | 0.657 |
| 132 | ENSG00000164692 | *COL1A2* | 0.101 | 0.618 | 0.943 | 0.731 |
| 133 | ENSG00000004975 | *DVL2* | 0.101 | 0.612 | 0.839 | 0.896 |
| 134 | ENSG00000140538 | *NTRK3* | 0.101 | 0.925 | 0.576 | 0.842 |
| 135 | ENSG00000180900 | *SCRIB* | 0.101 | 0.618 | 0.852 | 0.943 |
| 136 | ENSG00000138193 | *PLCE1* | 0.101 | 1.209 | 0.915 | 0.351 |
| 137 | ENSG00000121075 | *TBX4* | 0.101 | 0.925 | 0.564 | 0.882 |
| 138 | ENSG00000101384 | *JAG1* | 0.101 | 0.612 | 0.830 | 0.984 |
| 139 | ENSG00000174705 | *SH3PXD2B* | 0.100 | 1.240 | 0.339 | 0.829 |
| 140 | ENSG00000101439 | *CST3* | 0.100 | 0.925 | 0.765 | 0.596 |
| 141 | ENSG00000101076 | *HNF4A* | 0.100 | 0.743 | 0.793 | 0.704 |
| 142 | ENSG00000180772 | *AGTR2* | 0.100 | 0.724 | 0.827 | 0.728 |
| 143 | ENSG00000147601 | *TERF1* | 0.100 | 0.612 | 0.885 | 0.764 |
| 144 | ENSG00000134318 | *ROCK2* | 0.100 | 0.925 | 0.662 | 0.672 |
| 145 | ENSG00000155760 | *FZD7* | 0.099 | 0.612 | 0.941 | 0.878 |
| 146 | ENSG00000108821 | *COL1A1* | 0.099 | 0.612 | 0.966 | 0.752 |
| 147 | ENSG00000196455 | *PIK3R4* | 0.099 | 1.511 | 0.569 | 0.397 |
| 148 | ENSG00000233276 | *GPX1* | 0.099 | 0.925 | 0.882 | 0.562 |
| 149 | ENSG00000173801 | *JUP* | 0.099 | 0.562 | 0.983 | 0.884 |
| 150 | ENSG00000087085 | *ACHE* | 0.098 | 0.743 | 0.868 | 0.701 |
| 151 | ENSG00000107779 | *BMPR1A* | 0.097 | 0.612 | 0.832 | 0.930 |
| 152 | ENSG00000135476 | *ESPL1* | 0.097 | 0.881 | 0.577 | 0.747 |
| 153 | ENSG00000152661 | *GJA1* | 0.096 | 0.612 | 0.805 | 0.937 |
| 154 | ENSG00000138182 | *KIF20B* | 0.096 | 0.881 | 0.511 | 0.832 |
| 155 | ENSG00000081189 | *MEF2C* | 0.096 | 0.612 | 0.876 | 0.840 |
| 156 | ENSG00000140650 | *PMM2* | 0.095 | 1.240 | 0.332 | 0.777 |
| 157 | ENSG00000180176 | *TH* | 0.095 | 0.612 | 0.858 | 0.848 |
| 158 | ENSG00000125780 | *TGM3* | 0.094 | 0.934 | 0.771 | 0.535 |
| 159 | ENSG00000105270 | *CLIP3* | 0.094 | 0.925 | 0.780 | 0.543 |
| 160 | ENSG00000112297 | *AIM1* | 0.093 | 1.179 | 0.386 | 0.716 |
| 161 | ENSG00000122180 | *MYOG* | 0.093 | 0.743 | 0.792 | 0.731 |
| 162 | ENSG00000070018 | *LRP6* | 0.093 | 0.543 | 0.833 | 0.973 |
| 163 | ENSG00000167986 | *DDB1* | 0.093 | 0.925 | 0.547 | 0.773 |
| 164 | ENSG00000170370 | *EMX2* | 0.093 | 0.612 | 0.596 | 0.940 |
| 165 | ENSG00000012048 | *BRCA1* | 0.093 | 0.618 | 0.944 | 0.761 |
| 166 | ENSG00000079841 | *RIMS1* | 0.092 | 1.240 | 0.550 | 0.508 |
| 167 | ENSG00000070061 | *IKBKAP* | 0.092 | 1.105 | 0.293 | 0.980 |
| 168 | ENSG00000143801 | *PSEN2* | 0.092 | 0.925 | 0.576 | 0.793 |
| 169 | ENSG00000139292 | *LGR5* | 0.092 | 1.368 | 0.331 | 0.685 |
| 170 | ENSG00000039987 | *BEST2* | 0.091 | 2.203 | 0.515 | 0.146 |
| 171 | ENSG00000154118 | *JPH3* | 0.091 | 1.367 | 0.506 | 0.440 |
| 172 | ENSG00000100697 | *DICER1* | 0.091 | 0.925 | 0.822 | 0.520 |
| 173 | ENSG00000172867 | *KRT2* | 0.091 | 0.925 | 0.906 | 0.460 |
| 174 | ENSG00000206075 | *SERPINB5* | 0.091 | 1.558 | 0.297 | 0.565 |
| 175 | ENSG00000196584 | *XRCC2* | 0.091 | 0.612 | 0.753 | 0.905 |
| 176 | ENSG00000092295 | *TGM1* | 0.090 | 1.054 | 0.603 | 0.528 |
| 177 | ENSG00000071054 | *MAP4K4* | 0.090 | 0.925 | 0.668 | 0.601 |
| 178 | ENSG00000064300 | *NGFR* | 0.090 | 0.612 | 0.876 | 0.858 |
| 179 | ENSG00000085511 | *MAP3K4* | 0.090 | 0.925 | 0.670 | 0.645 |
| 180 | ENSG00000108602 | *ALDH3A1* | 0.089 | 1.879 | 0.497 | 0.248 |
| 181 | ENSG00000161638 | *ITGA5* | 0.089 | 0.618 | 0.875 | 0.849 |
| 182 | ENSG00000108773 | *KAT2A* | 0.089 | 0.612 | 0.661 | 0.978 |
| 183 | ENSG00000169032 | *MAP2K1* | 0.088 | 0.612 | 0.899 | 0.749 |
| 184 | ENSG00000010319 | *SEMA3G* | 0.088 | 2.001 | 0.550 | 0.148 |
| 185 | ENSG00000178999 | *AURKB* | 0.088 | 0.925 | 0.627 | 0.730 |
| 186 | ENSG00000133256 | *PDE6B* | 0.088 | 1.240 | 0.514 | 0.477 |
| 187 | ENSG00000054523 | *KIF1B* | 0.088 | 0.925 | 0.508 | 0.836 |
| 188 | ENSG00000112624 | *GLTSCR1L* | 0.087 | 1.558 | 0.258 | 0.593 |
| 189 | ENSG00000101680 | *LAMA1* | 0.087 | 0.793 | 0.642 | 0.888 |
| 190 | ENSG00000075275 | *CELSR1* | 0.086 | 0.571 | 0.802 | 0.886 |
| 191 | ENSG00000166164 | *BRD7* | 0.086 | 0.925 | 0.554 | 0.673 |
| 192 | ENSG00000112658 | *SRF* | 0.086 | 0.612 | 0.911 | 0.696 |
| 193 | ENSG00000170606 | *HSPA4* | 0.085 | 1.558 | 0.386 | 0.428 |
| 194 | ENSG00000117298 | *ECE1* | 0.084 | 0.612 | 0.790 | 0.896 |
| 195 | ENSG00000165240 | *ATP7A* | 0.084 | 0.455 | 0.957 | 0.930 |
| 196 | ENSG00000044115 | *CTNNA1* | 0.084 | 0.743 | 0.611 | 0.749 |
| 197 | ENSG00000138795 | *LEF1* | 0.084 | 0.612 | 0.917 | 0.610 |
| 198 | ENSG00000157890 | *MEGF11* | 0.084 | 1.879 | 0.277 | 0.299 |
| 199 | ENSG00000176697 | *BDNF* | 0.084 | 0.925 | 0.368 | 0.876 |
| 200 | ENSG00000057593 | *F7* | 0.084 | 0.612 | 0.952 | 0.667 |
