# Supplementary material for: Integrated rare variant-based risk gene prioritization in disease case-control sequencing studies
Source: PLoS Genet. 2017 Dec 27;13(12):e1007142. doi: 10.1371/journal.pgen.1007142 (PMC5760082; doi:10.1371/journal.pgen.1007142)
Supplement: S6 Table — This result is for 5987 genes in the CHD dataset with association signals of rare predicted deleterious variants that can be scored by network and phenotype. (DOCX) [file pgen.1007142.s027.docx]

| **S6 Table. Enriched biological process GO terms for top 50 genes based on IGSP integrated scoring for CHD.** | |
| --- | --- |
| GO term (David BP FAT) | *P** (Bonferroni) |
| GO:0007507 Heart development | 3.50E-18 |
| GO:0072358 Cardiovascular system development | 3.01E-16 |
| GO:0072359 Circulatory system development | 3.01E-16 |
| GO:0048646 Anatomical structure formation involved in morphogenesis | 3.35E-14 |
| GO:0009790 Embryo development | 1.27E-13 |
| GO:0048598 Embryonic morphogenesis | 6.08E-13 |
| GO:0009887 Organ morphogenesis | 4.87E-12 |
| GO:0048568 Embryonic organ development | 2.46E-11 |
| GO:0048729 Tissue morphogenesis | 4.56E-11 |
| GO:0048562 Embryonic organ morphogenesis | 5.72E-11 |
| GO:0048468 Cell development | 9.45E-10 |
| GO:0003007 Heart morphogenesis | 2.55E-09 |
| GO:0060429 Epithelium development | 3.15E-09 |
| GO:0048738 Cardiac muscle tissue development | 6.54E-09 |
| GO:0006928 Movement of cell or subcellular component | 1.07E-08 |
| GO:0002009 Morphogenesis of an epithelium | 1.27E-08 |
| GO:0014706 Striated muscle tissue development | 1.87E-08 |
| GO:0060537 Muscle tissue development | 3.39E-08 |
| GO:0007399 Nervous system development | 7.89E-08 |
| GO:0001944 Vasculature development | 1.19E-07 |
| GO:0007423 Sensory organ development | 1.33E-07 |
| GO:0090596 Sensory organ morphogenesis | 1.61E-07 |
| GO:0007417 Central nervous system development | 2.88E-07 |
| GO:0040011 Locomotion | 2.96E-07 |
| GO:0051270 Regulation of cellular component movement | 5.06E-07 |
| GO:0001568 Blood vessel development | 7.12E-07 |
| GO:0035295 Tube development | 7.80E-07 |
| GO:0022008 Neurogenesis | 9.01E-07 |
| GO:0060562 Epithelial tube morphogenesis | 1.54E-06 |
| GO:0048699 Generation of neurons | 2.44E-06 |
| GO:0030182 Neuron differentiation | 3.80E-06 |
| GO:0023051 Regulation of signaling | 3.90E-06 |
| GO:0035239 Tube morphogenesis | 5.40E-06 |
| GO:0043009 Chordate embryonic development | 8.86E-06 |
| GO:0009792 Embryo development ending in birth or egg hatching | 1.00E-05 |
| GO:0061061 Muscle structure development | 1.14E-05 |
| GO:0032989 Cellular component morphogenesis | 1.25E-05 |
| GO:0001501 Skeletal system development | 1.58E-05 |
| GO:0048514 Blood vessel morphogenesis | 1.62E-05 |
| GO:0010646 Regulation of cell communication | 1.67E-05 |
| GO:2000145 Regulation of cell motility | 1.70E-05 |
| GO:0042472 Inner ear morphogenesis | 1.79E-05 |
| GO:0048667 Cell morphogenesis involved in neuron differentiation | 1.82E-05 |
| GO:2000026 Regulation of multicellular organismal development | 1.89E-05 |
| GO:0000904 Cell morphogenesis involved in differentiation | 2.27E-05 |
| GO:0040012 Regulation of locomotion | 2.91E-05 |
| GO:0007166 Cell surface receptor signaling pathway | 4.33E-05 |
| GO:0045165 Cell fate commitment | 4.89E-05 |
| GO:0007389 Pattern specification process | 5.60E-05 |
| GO:0009966 Regulation of signal transduction | 6.72E-05 |
| GO:0042471 Ear morphogenesis | 6.89E-05 |
| GO:0007155 Cell adhesion | 1.01E-04 |
| GO:0022610 Biological adhesion | 1.07E-04 |
| GO:0001822 Kidney development | 1.10E-04 |
| GO:0009628 Response to abiotic stimulus | 1.51E-04 |
| GO:0048585 Negative regulation of response to stimulus | 1.56E-04 |
| GO:0072001 Renal system development | 1.81E-04 |
| GO:0009968 Negative regulation of signal transduction | 3.19E-04 |
| GO:0007167 Enzyme linked receptor protein signaling pathway | 4.38E-04 |
| GO:0030030 Cell projection organization | 5.04E-04 |
| GO:0048666 Neuron development | 5.05E-04 |
| GO:0001655 Urogenital system development | 5.16E-04 |
| GO:0003205 Cardiac chamber development | 5.72E-04 |
| GO:0060485 Mesenchyme development | 6.09E-04 |
| GO:0006935 Chemotaxis | 6.14E-04 |
| GO:0007420 Brain development | 6.19E-04 |
| GO:0042330 Taxis | 6.25E-04 |
| GO:0060445 Branching involved in salivary gland morphogenesis | 6.55E-04 |
| GO:0055007 Cardiac muscle cell differentiation | 7.11E-04 |
| GO:0030198 Extracellular matrix organization | 7.41E-04 |
| GO:0043062 Extracellular structure organization | 7.60E-04 |
| GO:0014065 Phosphatidylinositol 3-kinase signaling | 8.52E-04 |
| GO:0010648 Negative regulation of cell communication | 9.25E-04 |
| GO:0023057 Negative regulation of signaling | 9.66E-04 |
| GO:0060322 Head development | 1.07E-03 |
| GO:0007369 Gastrulation | 1.16E-03 |
| GO:0048839 Inner ear development | 1.46E-03 |
| GO:0007517 Muscle organ development | 1.60E-03 |
| GO:0071363 Cellular response to growth factor stimulus | 1.66E-03 |
| GO:0000902 Cell morphogenesis | 1.71E-03 |
| GO:0009967 Positive regulation of signal transduction | 1.79E-03 |
| GO:0048015 Phosphatidylinositol-mediated signaling | 1.83E-03 |
| GO:0055008 Cardiac muscle tissue morphogenesis | 2.03E-03 |
| GO:0048017 Inositol lipid-mediated signaling | 2.04E-03 |
| GO:0051094 Positive regulation of developmental process | 2.19E-03 |
| GO:0001704 Formation of primary germ layer | 2.27E-03 |
| GO:0003206 Cardiac chamber morphogenesis | 2.27E-03 |
| GO:0009605 Response to external stimulus | 2.37E-03 |
| GO:0070848 Response to growth factor | 2.42E-03 |
| GO:0035051 Cardiocyte differentiation | 2.76E-03 |
| GO:0030155 Regulation of cell adhesion | 2.78E-03 |
| GO:0009612 Response to mechanical stimulus | 3.31E-03 |
| GO:0043583 Ear development | 3.65E-03 |
| GO:0007422 Peripheral nervous system development | 4.00E-03 |
| GO:0001525 Angiogenesis | 4.35E-03 |
| GO:0007409 Axonogenesis | 4.35E-03 |
| GO:0048705 Skeletal system morphogenesis | 4.57E-03 |
| GO:0060415 Muscle tissue morphogenesis | 4.59E-03 |
| GO:0048812 Neuron projection morphogenesis | 4.85E-03 |
| GO:0045595 Regulation of cell differentiation | 5.07E-03 |
| GO:0048732 Gland development | 5.10E-03 |
| GO:0030334 Regulation of cell migration | 5.16E-03 |
| GO:0007435 Salivary gland morphogenesis | 5.38E-03 |
| GO:0010647 Positive regulation of cell communication | 5.57E-03 |
| GO:0023056 Positive regulation of signaling | 5.96E-03 |
| GO:0007411 Axon guidance | 6.60E-03 |
| GO:0030855 Epithelial cell differentiation | 6.70E-03 |
| GO:0097485 Neuron projection guidance | 6.80E-03 |
| GO:0048644 Muscle organ morphogenesis | 7.24E-03 |
| GO:0003002 Regionalization | 7.90E-03 |
| GO:0061564 Axon development | 8.02E-03 |
| GO:0035107 Appendage morphogenesis | 8.49E-03 |
| GO:0035108 Limb morphogenesis | 8.49E-03 |
| GO:0007431 Salivary gland development | 8.59E-03 |
| GO:0022603 Regulation of anatomical structure morphogenesis | 9.21E-03 |
| GO:0051240 Positive regulation of multicellular organismal process | 1.03E-02 |
| GO:0030900 Forebrain development | 1.56E-02 |
| GO:0040007 Growth | 1.59E-02 |
| GO:0042692 Muscle cell differentiation | 1.59E-02 |
| GO:0003279 Cardiac septum development | 1.77E-02 |
| GO:0048736 Appendage development | 1.88E-02 |
| GO:0060173 Limb development | 1.88E-02 |
| GO:0048858 Cell projection morphogenesis | 2.19E-02 |
| GO:0061138 Morphogenesis of a branching epithelium | 2.36E-02 |
| GO:0021761 Limbic system development | 2.38E-02 |
| GO:0030323 Respiratory tube development | 2.60E-02 |
| GO:0035272 Exocrine system development | 2.67E-02 |
| GO:0032990 Cell part morphogenesis | 2.76E-02 |
| GO:0031175 Neuron projection development | 2.76E-02 |
| GO:0042127 Regulation of cell proliferation | 2.77E-02 |
| GO:0051216 Cartilage development | 2.85E-02 |
| GO:2001239 Regulation of extrinsic apoptotic signaling pathway in absence of ligand | 3.12E-02 |
| GO:0000165 MAPK cascade | 3.61E-02 |
| GO:0008284 Positive regulation of cell proliferation | 3.94E-02 |
| GO:0001763 Morphogenesis of a branching structure | 4.07E-02 |
| GO:0022612 Gland morphogenesis | 4.22E-02 |
| GO:0008544 Epidermis development | 4.51E-02 |
| GO:0003231 Cardiac ventricle development | 4.77E-02 |
