# Supplementary material for: Integrated rare variant-based risk gene prioritization in disease case-control sequencing studies
Source: PLoS Genet. 2017 Dec 27;13(12):e1007142. doi: 10.1371/journal.pgen.1007142 (PMC5760082; doi:10.1371/journal.pgen.1007142)
Supplement: S7 Table — This result is for 5987 genes in the CHD dataset with association signals of rare predicted deleterious variants that can be scored by network and phenotype. (DOCX) [file pgen.1007142.s028.docx]

| **S7 Table. Top 10 biological process GO terms for top 50 genes based on association *P*-values from burden test for CHD.** | |
| --- | --- |
| GO term (David BP FAT) | *P** (Bonferroni) |
| GO:0048646 Anatomical structure formation involved in morphogenesis | 3.45E-02 |
| GO:0008544 Epidermis development | 3.49E-01 |
| GO:0032989 Cellular component morphogenesis | 4.65E-01 |
| GO:0071600 Otic vesicle morphogenesis | 4.94E-01 |
| GO:0030198 Extracellular matrix organization | 5.33E-01 |
| GO:0043062 Extracellular structure organization | 5.39E-01 |
| GO:0003007 Heart morphogenesis | 6.37E-01 |
| GO:0048667 Cell morphogenesis involved in neuron differentiation | 6.50E-01 |
| GO:0007423 Sensory organ development | 6.98E-01 |
| GO:0009628 Response to abiotic stimulus | 7.19E-01 |
