# Supplementary material for: Integrated rare variant-based risk gene prioritization in disease case-control sequencing studies
Source: PLoS Genet. 2017 Dec 27;13(12):e1007142. doi: 10.1371/journal.pgen.1007142 (PMC5760082; doi:10.1371/journal.pgen.1007142)
Supplement: S8 Table — (DOCX) [file pgen.1007142.s029.docx]

**S8 Table. The required sampling steps and convergence time of IGSP.**

| *x* (%) | Sampling steps | | Convergence Time | |
| --- | --- | --- | --- | --- |
|  | CHD case study | SCZ simulation | CHD case study | SCZ simulation |
| 1 | 4000 | 4000 | 3 mins | 6 mins |
| 2 | 5000 | 5000 | 4 mins | 8 mins |
| 3 | 6000 | 6000 | 5 mins | 9 mins |
| 4 | 7000 | 7000 | 6 mins | 11 mins |
| 5 | 7000 | 7000 | 6 mins | 11 mins |
| 6 | 8000 | 8000 | 7 mins | 14 mins |
| 7 | 9000 | 8000 | 9 mins | 14 mins |
| 8 | 9000 | 9000 | 9 mins | 16 mins |
| 9 | 10000 | 9000 | 10 mins | 16 mins |
| 10 | 10000 | 10000 | 10 mins | 18 mins |

The hardware platform to execute IGSP: MacBook Pro (CPU: 2.6 GHz, RAM: 16GB)
