# Supplementary material for: Integrated rare variant-based risk gene prioritization in disease case-control sequencing studies
Source: PLoS Genet. 2017 Dec 27;13(12):e1007142. doi: 10.1371/journal.pgen.1007142 (PMC5760082; doi:10.1371/journal.pgen.1007142)
Supplement: S9 Table — This result is for 11840 genes in the CHD dataset with association signals of rare predicted deleterious variants that can be scored by network. (DOCX) [file pgen.1007142.s030.docx]

| **S9 Table. Enriched biological process GO terms for top 100 genes based on IGSP network-based scoring for CHD.** | |
| --- | --- |
| GO term (David BP FAT) | *P** (Bonferroni) |
| GO:0072358 Cardiovascular system development | 1.69E-04 |
| GO:0072359 Circulatory system development | 1.69E-04 |
| GO:0048468 Cell development | 1.91E-04 |
| GO:0000904 Cell morphogenesis involved in differentiation | 6.00E-04 |
| GO:0032989 Cellular component morphogenesis | 2.82E-03 |
| GO:0022603 Regulation of anatomical structure morphogenesis | 4.31E-03 |
| GO:0048584 Positive regulation of response to stimulus | 4.63E-03 |
| GO:0009967 Positive regulation of signal transduction | 1.06E-02 |
| GO:0030182 Neuron differentiation | 1.19E-02 |
| GO:0010647 Positive regulation of cell communication | 1.29E-02 |
| GO:0023056 Positive regulation of signaling | 1.41E-02 |
| GO:0000902 Cell morphogenesis | 1.42E-02 |
| GO:0048646 Anatomical structure formation involved in morphogenesis | 1.70E-02 |
| GO:0007507 Heart development | 1.79E-02 |
| GO:0007155 Cell adhesion | 1.81E-02 |
| GO:0022610 Biological adhesion | 1.92E-02 |
| GO:2000026 Regulation of multicellular organismal development | 2.26E-02 |
| GO:0048015 Phosphatidylinositol-mediated signaling | 2.82E-02 |
| GO:0048729 Tissue morphogenesis | 3.07E-02 |
| GO:0048017 Inositol lipid-mediated signaling | 3.17E-02 |
| GO:1902533 Positive regulation of intracellular signal transduction | 4.00E-02 |
| GO:0035556 Intracellular signal transduction | 4.31E-02 |
| GO:0006928 Movement of cell or subcellular component | 4.65E-02 |
| GO:2000145 Regulation of cell motility | 4.65E-02 |
