# Supplementary material for: Integrated rare variant-based risk gene prioritization in disease case-control sequencing studies
Source: PLoS Genet. 2017 Dec 27;13(12):e1007142. doi: 10.1371/journal.pgen.1007142 (PMC5760082; doi:10.1371/journal.pgen.1007142)
Supplement: S10 Table — This result is for 11840 genes in the CHD dataset with association signals of rare predicted deleterious variants that can be scored by network. (DOCX) [file pgen.1007142.s031.docx]

| **S10 Table. Top 10 biological process GO terms for top 100 genes based on association *P*-values from burden test for CHD.** | |
| --- | --- |
| GO term (David BP FAT) | *P** (Bonferroni) |
| GO:0007155 Cell adhesion | 0.698367 |
| GO:0022610 Biological adhesion | 0.71466 |
| GO:0007010 Cytoskeleton organization | 0.898587 |
| GO:0048646 Anatomical structure formation involved in morphogenesis | 0.912335 |
| GO:0071600 Otic vesicle morphogenesis | 0.937885 |
| GO:0030198 Extracellular matrix organization | 0.990518 |
| GO:0043062 Extracellular structure organization | 0.99124 |
| GO:0071599 Otic vesicle development | 0.9983 |
| GO:0030030 Cell projection organization | 0.999684 |
| GO:0001704 Formation of primary germ layer | 0.999748 |
