# Supplementary material for: Integrated rare variant-based risk gene prioritization in disease case-control sequencing studies
Source: PLoS Genet. 2017 Dec 27;13(12):e1007142. doi: 10.1371/journal.pgen.1007142 (PMC5760082; doi:10.1371/journal.pgen.1007142)
Supplement: S11 Table — (DOCX) [file pgen.1007142.s032.docx]

| **S11 Table. Curated genetic modifier genes and variants of CHD in 22q11.2DS for future validation.** | | | | | | | | | | | |
| --- | --- | --- | --- | --- | --- | --- | --- | --- | --- | --- | --- |
| Gene | | | | | | Variant | | | | | |
| Name | B | *P* | Net | Phe | Rank | Coordinate (hg19) | Ref:Alt | B | Functional Class | AAF | C |
| ***FGFR2*** | 6:0 | 0.0063 | 0.981 | 0.988 | 1 | chr10:123247542 | T:C | 1:0 | Missense variant | 0.0027:NA | 26.0 |
|  |  |  |  |  |  | chr10:123256135 | G:A | 1:0 | Missense variant | 0.0027:0.001 | 34.0 |
|  |  |  |  |  |  | chr10:123279540 | C:T | 1:0 | Missense variant | 0.0027:NA | 35.0 |
|  |  |  |  |  |  | chr10:123325158 | G:A | 3:0 | Missense variant | 0.0082:0.004 | 22.4 |
| ***EYA1*** | 4:0 | 0.028 | 0.786 | 0.959 | 10 | chr8:72267083 | G:C | 3:0 | Missense variant | 0.0082:0.001 | 20.6 |
|  |  |  |  |  |  | chr8:72267127 | T:G | 1:0 | Missense variant | 0.0027:NA | 30.0 |
| ***AXIN1*** | 3:0 | 0.058 | 0.972 | 0.935 | 11 | chr16:364544 | C:T | 1:0 | Missense splice variant | 0.0027:NA | 25.7 |
|  |  |  |  |  |  | chr16:396911 | G:A | 2:0 | Missense variant | 0.0054:NA | 20.4 |
| ***FGFR1*** | 3:0 | 0.058 | 0.898 | 0.997 | 14 | chr8:38271243 | CCT:C | 1:0 | Frame shift variant | 0.0027:NA | 35.0 |
|  |  |  |  |  |  | chr8:38273467 | T:C | 1:0 | Missense variant | 0.0027:NA | 22.2 |
|  |  |  |  |  |  | chr8:38287327 | G:C | 1:0 | Missense variant | 0.0027:NA | 22.5 |
| ***ROR2*** | 3:0 | 0.058 | 0.812 | 0.982 | 21 | chr9:94487187 | C:T | 1:0 | Missense variant | 0.0027:0.005 | 20.2 |
|  |  |  |  |  |  | chr9:94519639 | G:T | 1:0 | Missense variant | 0.0027:NA | 23.8 |
|  |  |  |  |  |  | chr9:94538053 | C:A | 1:0 | Missense variant | 0.0027:NA | 24.8 |
| ***CALR*** | 3:0 | 0.058 | 0.929 | 0.776 | 24 | chr19:13051058 | A:G | 2:0 | Missense splice variant | 0.0054:NA | 22.9 |
|  |  |  |  |  |  | chr19:13054590 | G:A | 1:0 | Missense variant | 0.0027:NA | 22.3 |
| ***NKX2-5*** | 2:0 | 0.119 | 0.974 | 0.933 | 27 | chr5:172662026 | C:G | 2:0 | Missense variant | 0.0054:NA | 26.4 |
| ***BMP7*** | 2:0 | 0.119 | 0.945 | 0.914 | 30 | chr20:55777539 | G:A | 2:0 | Missense variant | 0.0054:0.001 | 29.0 |
| ***NF1*** | 2:0 | 0.119 | 0.979 | 0.891 | 33 | chr17:29677233 | C:T | 1:0 | Missense variant | 0.0027:<0.001 | 32.0 |
|  |  |  |  |  |  | chr17:29685600 | T:G | 1:0 | Missense variant | 0.0027:NA | 23.7 |
| ***GATA4*** | 2:0 | 0.119 | 0.816 | 0.968 | 36 | chr8:11612576 | C:A | 1:0 | Synonymous variant | 0.0027:NA | 20.5 |
|  |  |  |  |  |  | chr8:11615955 | G:A | 1:0 | Missense variant | 0.0027:NA | 25.1 |
| ***PCSK5*** | 2:0 | 0.119 | 0.849 | 0.946 | 45 | chr9:78506189 | G:A | 1:0 | Missense variant | 0.0027:0.001 | 26.0 |
|  |  |  |  |  |  | chr9:78965816 | G:A | 1:0 | Missense variant | 0.0027:0.001 | 23.5 |
| ***ERBB3*** | 2:0 | 0.119 | 0.787 | 0.939 | 48 | chr12:56477541 | C:T | 1:0 | Missense variant | 0.0027:0.002 | 23.0 |
|  |  |  |  |  |  | chr12:56487316 | C:T | 1:0 | Missense variant | 0.0027:NA | 33.0 |
| ***ACVRL1*** | 2:0 | 0.119 | 0.918 | 0.850 | 51 | chr12:52312870 | A:G | 1:0 | Missense variant | 0.0027:NA | 21.1 |
|  |  |  |  |  |  | chr12:52314610 | C:T | 1:0 | Missense variant | 0.0027:0.001 | 27.3 |
| ***PAXIP1*** | 2:0 | 0.119 | 0.769 | 0.860 | 59 | chr7:154760727 | G:A | 1:0 | Missense variant | 0.0027:NA | 22.9 |
|  |  |  |  |  |  | chr7:154767477 | C:T | 1:0 | Missense variant | 0.0027:0.003 | 23.0 |
| ***GLMN*** | 2:0 | 0.119 | 0.758 | 0.797 | 73 | chr1:92733663 | A:G | 1:0 | Missense splice variant | 0.0027:<0.001 | 28.6 |
|  |  |  |  |  |  | chr1:92754532 | C:A | 1:0 | Missense variant | 0.0027:<0.001 | 24.0 |
| ***WNT3A*** | 1:0 | 0.245 | 0.914 | 0.967 | 82 | chr1:228238465 | G:A | 1:0 | Missense variant | 0.0027:NA | 23.1 |
| ***WNT5A*** | 1:0 | 0.245 | 0.988 | 0.948 | 86 | chr3:55504326 | C:T | 1:0 | Missense variant | 0.0027:NA | 21.0 |
| ***PAX2*** | 1:0 | 0.245 | 0.953 | 0.901 | 92 | chr10:102584417 | C:T | 1:0 | Missense variant | 0.0027:<0.001 | 23.6 |
| ***EGFR*** | 1:0 | 0.245 | 0.972 | 0.975 | 98 | chr7:55259497 | A:G | 1:0 | Missense variant | 0.0027:NA | 30.0 |
| ***RB1*** | 1:0 | 0.245 | 0.902 | 0.959 | 102 | chr13:49050882 | G:A | 1:0 | Missense variant | 0.0027:<0.001 | 33.0 |
| ***IGF1*** | 1:0 | 0.245 | 0.984 | 0.889 | 103 | chr12:102813337 | C:T | 1:0 | Missense variant | 0.0027:0.001 | 24.1 |
| ***BMP2*** | 1:0 | 0.245 | 0.918 | 0.909 | 110 | chr20:6750950 | C:A | 1:0 | Missense variant | 0.0027:NA | 24.8 |
| ***SOX2*** | 1:0 | 0.245 | 0.860 | 0.961 | 114 | chr3:181430204 | G:T | 1:0 | Missense variant | 0.0027:NA | 24.3 |
| ***VEGFA*** | 1:0 | 0.245 | 0.825 | 0.985 | 116 | chr6:43738966 | G:T | 1:0 | Missense variant | 0.0027:NA | 26.8 |
| ***FGF8*** | 1:0 | 0.245 | 0.807 | 0.985 | 126 | chr10:103530240 | G:A | 1:0 | Missense variant | 0.0027:NA | 28.8 |
| ***MDFI*** | 1:0 | 0.245 | 0.905 | 0.830 | 128 | chr6:41617480 | G:A | 1:0 | Missense variant | 0.0027:<0.001 | 25.8 |
| ***SNAI1*** | 1:0 | 0.245 | 0.777 | 0.935 | 129 | chr20:48600475 | C:T | 1:0 | Missense variant | 0.0027:<0.001 | 21.3 |
| ***DVL2*** | 1:0 | 0.245 | 0.839 | 0.896 | 133 | chr17:7131299 | G:C | 1:0 | Missense variant | 0.0027:NA | 26.9 |
| ***JAG1*** | 1:0 | 0.245 | 0.830 | 0.984 | 138 | chr20:10625624 | C:T | 1:0 | Missense variant | 0.0027:0.002 | 24.1 |
| ***TERF1*** | 1:0 | 0.245 | 0.885 | 0.764 | 143 | chr8:73958364 | G:A | 1:0 | Missense variant | 0.0027:0.001 | 33.0 |
| ***FZD7*** | 1:0 | 0.245 | 0.941 | 0.878 | 145 | chr2:202899645 | T:C | 1:0 | Missense variant | 0.0027:NA | 23.3 |
| ***COL1A1*** | 1:0 | 0.245 | 0.966 | 0.752 | 146 | chr17:48274551 | G:A | 1:0 | Missense variant | 0.0027:NA | 25.3 |
| ***BMPR1A*** | 1:0 | 0.245 | 0.832 | 0.930 | 151 | chr10:88676948 | T:A | 1:0 | Missense variant | 0.0027:NA | 32.0 |
| ***GJA1*** | 1:0 | 0.245 | 0.805 | 0.937 | 153 | chr6:121769120 | G:A | 1:0 | Missense variant | 0.0027:NA | 25.3 |
| ***MEF2C*** | 1:0 | 0.245 | 0.876 | 0.840 | 155 | chr5:88027697 | C:T | 1:0 | Missense variant | 0.0027:NA | 25.2 |
| ***TH*** | 1:0 | 0.245 | 0.858 | 0.848 | 157 | chr11:2185482 | G:A | 1:0 | Missense variant | 0.0027:NA | 26.0 |
| ***XRCC2*** | 1:0 | 0.245 | 0.753 | 0.905 | 175 | chr7:152346008 | G:A | 1:0 | Missense variant | 0.0027:NA | 28.2 |
| ***NGFR*** | 1:0 | 0.245 | 0.876 | 0.858 | 178 | chr17:47590272 | C:T | 1:0 | Synonymous variant | 0.0027:0.003 | 20.3 |
| ***ECE1*** | 1:0 | 0.245 | 0.790 | 0.896 | 194 | chr1:21554495 | C:T | 1:0 | Synonymous variant | 0.0027:<0.001 | 20.9 |
| B denotes the ratio of the counts of rare predicted deleterious alleles in cases to that in controls; *P* denotes *P*-value from the burden test; Net and Phe represent the average network and phenotype scores, respectively; AAF denotes alternative allele frequency (the cohort of sequencing data: 1000 Genome EUR population); C denotes Combined Annotation Dependent Depletion (CADD) scaled score. | | | | | | | | | | | |
